# Roles of RRM2 and RRM2B in pyrimidine stress responses and differentiation of acute myeloid leukemia cells

**DOI:** 10.1038/s41420-026-03105-y

**Published:** 2026-04-24

**Authors:** Alojzija Brcic, Hrvoje Lalic, Tomislav Smoljo, Klara Bardač, Vilma Dembitz, Romana Penker, Giovanny Rodriguez Blanco, Antonio Bedalov, Dora Visnjic

**Affiliations:** 1 Laboratory for Cell Biology, Department of Physiology, Croatian Institute for Brain Research, University of Zagreb School of Medicine, Zagreb, Croatia; 2 Department of Laboratory Immunology, Clinical Department of Laboratory Diagnostics, University Hospital Center Zagreb, Zagreb, Croatia; 3 Clinical Institute of Medical and Chemical Laboratory Diagnostics, Medical University of Graz, Graz, Austria; 4 Clinical Research Division, Fred Hutchinson Cancer Research Centre, Seattle, WA USA; 5 Present Address: Department of Oncology, University Hospital Center Split, Split, Croatia

**Keywords:** Cancer metabolism, Acute myeloid leukaemia

## Abstract

Differentiation therapy offers a promising approach in acute myeloid leukemia (AML) by overcoming the developmental block that maintains leukemic blasts. Increasing evidence indicates that DNA replication stress can promote differentiation rather than cytotoxicity; however, the metabolic mechanisms linking replication stress to differentiation remain poorly defined. Here, we investigated how perturbations in nucleotide metabolism regulate replication stress–driven differentiation. Using metabolomic and functional analyses in AML cell lines, we show that agents inducing differentiation through replication stress, including 5-aminoimidazole-4-carboxamide ribonucleoside (AICAr), dihydroorotate dehydrogenase (DHODH) inhibition, and low-dose cytarabine, converge on disruption of nucleotide pool balance. Low-dose AICAr induced a pyrimidine–purine imbalance, S phase arrest, and enhanced differentiation, whereas high-dose reduced these effects. Although brequinar and cytarabine altered nucleotide metabolism through distinct mechanisms, differentiation induced by all agents was abolished by supplementation with high levels of ribo- and deoxyribonucleosides, confirming that nucleotide imbalance is a central driver. We further identify ribonucleotide reductase (RNR) as a critical modulator of this process. Replication stress induced context-dependent regulation of RNR subunits, with RRM2 upregulated in p53-mutant U937 cells and the p53-responsive RRM2B isoform predominating in p53-wild-type MOLM-13 cells. Consistent with these differences, RRM2 depletion enhanced differentiation in U937 cells without affecting viability but impaired differentiation and survival in MOLM-13 cells. These findings position nucleotide metabolism as a key regulator of AML differentiation and suggest that combining RNR-targeted and checkpoint-modulating strategies could optimize therapeutic responses.

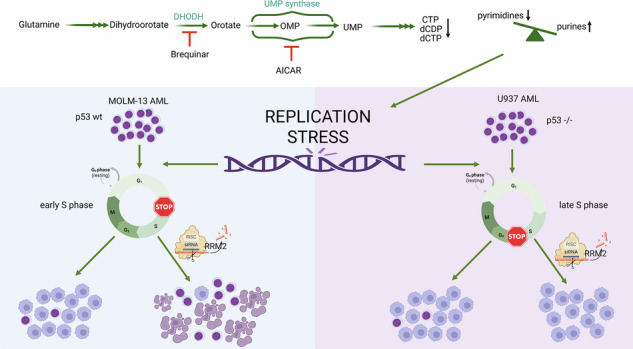

## Introduction

Increasing evidence suggests that DNA replication stress (RS) can drive leukemic cells toward differentiation rather than cell death, highlighting differentiation as a promising therapeutic strategy [1–6]. Unlike conventional cytotoxic approaches aimed at eradicating tumor cells, often poorly tolerated in older acute myeloid leukemia (AML) patients with comorbidities, differentiation therapy relieves the developmental block that defines AML, pushing blasts toward terminal maturation and loss of proliferative potential. The clinical success of all-trans retinoic acid (ATRA) in acute promyelocytic leukemia (APL) and isocitrate dehydrogenase (IDH) inhibitors in IDH-mutated AML underscores the therapeutic relevance of differentiation-based strategies [7, 8].

A number of pharmacologic agents have been shown to trigger differentiation by inducing replication stress. Low doses of cytarabine (AraC), a cornerstone of AML chemotherapy [8–10], and pyrimidine synthesis inhibitors such as dihydroorotate dehydrogenase (DHODH) inhibitors [11] both promote differentiation, albeit through distinct mechanisms. Pyrimidine synthesis inhibitors deplete deoxynucleotide triphosphate (dNTP) pools, stalling replication forks, whereas AraC is metabolized to Ara-CTP, which competes with deoxycytidine triphosphate (dCTP) during DNA synthesis [12, 13]. We previously showed that exogenous 5-aminoimidazole-4-carboxamide ribonucleoside (AICAr, acadesine) promoted differentiation of AML cell lines and primary blasts [14–16], suggesting that increases in cellular AICAR levels influence myeloid maturation. Recent studies in other hematopoietic and leukemia models support this idea, showing that interventions elevating endogenous AICAR, such as knockdown of folate pathway enzymes methylenetetrahydrofolate dehydrogenase 2 (MTHFD2) or serine hydroxymethyltransferase 2 (SHMT2), or mild folate depletion, promote differentiation alongside metabolic changes resembling those induced by AICAr supplementation [17, 18]. Although AICAr is widely used as an AMPK activator, analogous to PMA in PKC activation, all studies indicate that its differentiation effects are AMPK-independent [14, 17–19]. Our results instead pointed that the mechanism of differentiation is more similar to DHODH inhibition and consistent with inhibition of uridine monophosphate (UMP) synthase downstream of DHODH. Like the DHODH inhibitor brequinar, AICAr induced S phase arrest and their differentiation effects in U937 cells were suppressed by downregulation of checkpoint kinase 1 (Chk1), a central mediator of the DNA damage and replication stress response [4]. Similarly, low-dose AraC triggered S phase arrest and differentiation, suggesting that both classes promote AML differentiation through a shared mechanism, likely mediated by replication stress [9].

In our previous studies on AICAr-induced differentiation in U937 cells, we measured only orotate and uridine monophosphate (UMP) levels, suggesting UMP synthase inhibition. Interestingly, AICAr’s effects were biphasic: the low dose strongly increased orotate and promoted differentiation, whereas the high dose had a weaker effect and even reduced the differentiation-inducing effect of brequinar when administered together [4]. These findings, together with reports that differentiation can result from overall dNTP imbalances rather than from single nucleotide depletion, suggest that metabolic perturbations underlie replication stress–induced differentiation [6, 20–22].

Recent work implicates ribonucleotide reductase (RNR) in the regulation of AML differentiation [20]. RNR, a key enzyme maintaining dNTP balance, is a heterodimeric tetramer composed of two identical large subunits (RRM1) and two identical small subunits (RRM2 or RRM2B). RRM1 levels are constant throughout the cell cycle and are always in excess of RRM2. RRM2, expressed during S phase and degraded after mitotic entry, supports DNA replication, whereas RRM2B (or p53R2) is a p53-inducible isoform activated in response to DNA damage, sustaining DNA repair and mitochondrial DNA synthesis [23–25]. RNR hyperactivation or RRM2 upregulation was reported to overcome differentiation arrest in AML models, including U937 cells treated with nelarabine [20]. These observations suggest that perturbations in RNR activity and dNTP homeostasis may represent a common pathway linking replication stress to leukemic differentiation.

In this study, we investigate the metabolic basis of replication stress–induced differentiation in AML cell lines, with a particular focus on the contributions of RNR subunits, and define this process as a metabolically regulated pathway that can be therapeutically exploited.

## Results

### AICAr exhibits a biphasic effect on UMP synthase inhibition in de novo pyrimidine synthesis and disrupts purine metabolism in a dose-dependent manner

To systematically investigate how AICAr, brequinar, and AraC affect nucleotide metabolism and differentiation in AML cells, we performed a comprehensive metabolomic analysis in U937 cells, focusing on pyrimidine and purine pathways and their relationship to differentiation.

**Fig. 1:**
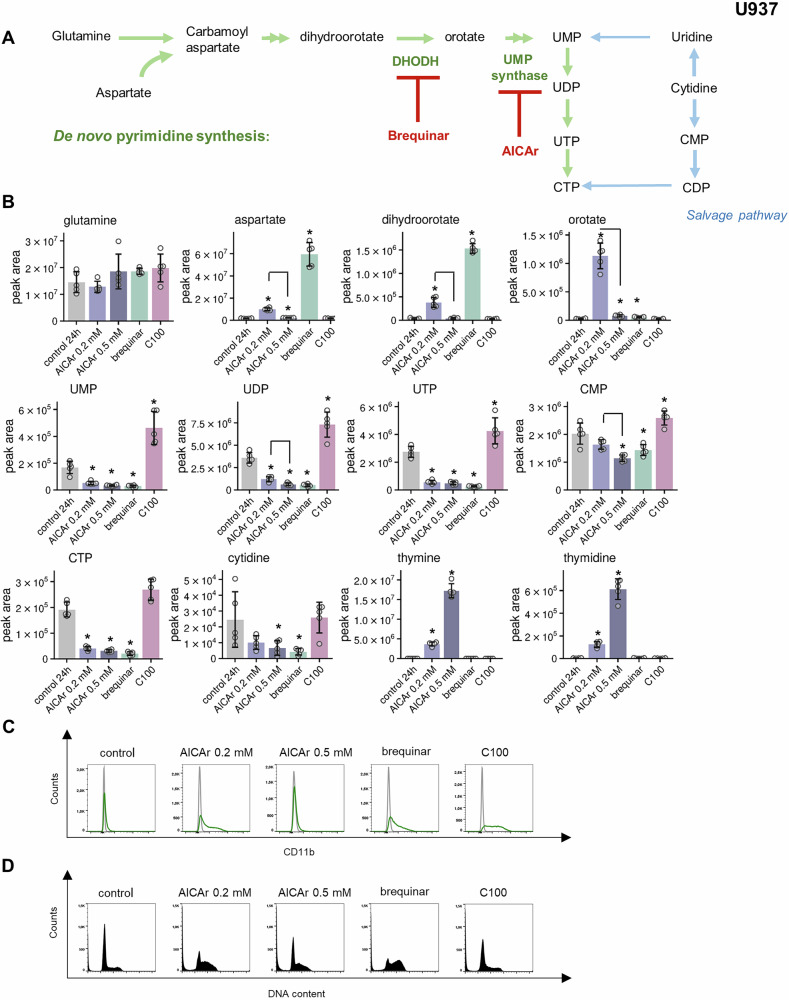
Pyrimidine Metabolism in U937 cells upon AICAr, brequinar, and AraC treatment. **A** Schematic representation of pyrimidine de novo synthesis and salvage pathway. **B** U937 cells were treated with AICAr (0.2 or 0.5 mM), brequinar (0.5 μM), or 100 nM AraC (C100) for 24 h. Intracellular levels of pyrimidine pathway metabolites were measured by LC/MS. Data are representative of three independent experiments performed in quintuplicate. Results are expressed as mean ± SD *P < 0.05 vs. control—indicates *P* < 0.05 between experimental groups. Peak area values represent normalized metabolite levels (per 10⁶ cells). **C** Representative flow cytometry histograms showing CD11b expression (green line) compared to isotype control (black line). **D** Representative histograms of propidium iodide (PI)-stained cells from three independent experiments analyzed by flow cytometry. DHODH dihydroorotate dehydrogenase, UMP uridine monophosphate, AICAr 5-aminoimidazole-4-carboxamide riboside, UDP uridine diphosphate, UTP uridine triphosphate, CTP cytidine triphosphate, CMP cytidine monophosphate, CDP cytidine diphosphate.

Figure 1A provides a schematic overview of de novo pyrimidine synthesis and the salvage pathway, highlighting the key enzymatic steps targeted in this study. Brequinar inhibits dihydroorotate dehydrogenase (DHODH), whereas AICAr acts downstream at the level of uridine monophosphate (UMP) synthase, which is reflected in the distinct metabolomic profiles observed in Fig. 1B.

Examining de novo pyrimidine synthesis (Fig. 1B), both low-dose AICAr and brequinar increased aspartate, with brequinar showing the stronger effect. Consistent with DHODH inhibition, brequinar raised dihydroorotate while lowering orotate, whereas AICAr increased both, particularly at low doses. These changes coincided with CD11b induction (Fig. 1C) and S phase arrest (Fig. 1D and Supplementary Fig. 1A). Both agents strongly suppressed UMP, UDP, UTP, CMP, and CTP, while AraC led to their partial accumulation. Notably, AICAr dose-dependently elevated thymine and thymidine, an effect absent with brequinar (Fig. 1B).

**Fig. 2:**
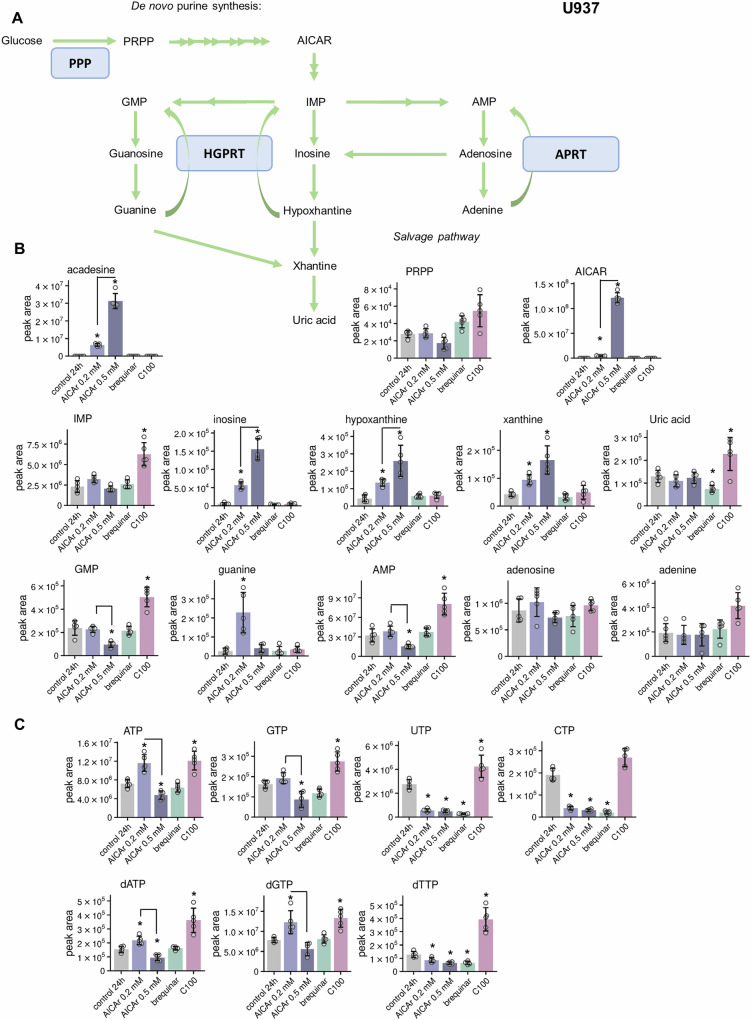
Altered purine metabolism and nucleotide pools in U937 cells treated with AICAr, brequinar, or AraC. **A** Schematic representation of purine de novo synthesis and salvage pathways. **B** U937 cells were treated with AICAr (0.2 or 0.5 mM), brequinar (0.5 μM), or 100 nM AraC (C100) for 24 h. Intracellular levels of purine pathway metabolites were measured by LC/MS. **C** Intracellular levels of purine and pyrimidine ribonucleotides and deoxyribonucleotides measured by LC/MS after 24 h treatment with AICAr, brequinar, or AraC. Data are representative of three independent experiments performed in quintuplicate. Results are expressed as mean ± SD *P < 0.05 vs. control—indicates *P* < 0.05 between experimental groups. PRPP phosphoribosyl pyrophosphate, AICAr 5-aminoimidazole-4-carboxamide ribonucleoside, IMP inosine monophosphate, AMP adenosine monophosphate, GMP guanosine monophosphate, HGPRT hypoxanthine-guanine phosphoribosyltransferase, APRT adenine phosphoribosyltransferase, PPP pentose phosphate pathway, ATP adenosine triphosphate, dATP deoxyadenosine triphosphate, GTP guanosine triphosphate, dGTP deoxyguanosine triphosphate, dTTP deoxythymidine triphosphate, CTP cytidine triphosphate.

In de novo purine synthesis (Fig. 2A, B), exogenous AICAr accumulated as ribonucleoside (acadesine) and its phosphorylated form, AICAR, an endogenous purine precursor. Increases in hypoxanthine, inosine, and guanine suggested reduced HGPRT activity [26, 27]. Low-dose AICAr or brequinar had little effect on IMP, AMP, or GMP, but high-dose AICAr decreased AMP and GMP, whereas AraC markedly elevated IMP, AMP, and GMP.

Overall, metabolomic analysis showed brequinar selectively inhibits DHODH, while AICAr disrupts both purine and pyrimidine metabolism. AICAr caused stronger dihydroorotate/orotate accumulation at low dose, increased thymine/thymidine dose-dependently, and reduced AMP and GMP at high dose.

### Nucleotide imbalance correlates with differentiation

Heatmap clustering confirmed dose-dependent effects of AICAr on pyrimidine metabolites (Supplementary Fig. 2A). Although no metabolite was uniformly up- or downregulated across all highly differentiating conditions, several ribonucleotides and deoxyribonucleotides were consistently among the most significantly altered (Supplementary Fig. 2B).

To identify distinct patterns, we compared nucleotide triphosphates (NTPs) and dNTPs. Low-dose AICAr increased ATP, dATP, and dGTP, whereas high-dose AICAr decreased ATP, GTP, and dATP. Both AICAr and brequinar reduced CTP and UTP, while AraC increased all NTPs and dNTPs (Fig. 2C).

Given differences in purine dNTP levels between low- and high-dose AICAr treatments, we tested exogenous deoxyadenosine (dA) and deoxyguanosine (dG) at 10 μM, previously reported to induce differentiation [20]. The nucleosides had no effect on viability or differentiation in control, AICAr-, or AraC-treated cells, but modestly increased CD11b in brequinar-treated cells (Supplementary Fig. 3A, B). This suggests differentiation effects may arise from exacerbated imbalances between specific dNTPs, at least for brequinar-treated cells. To determine whether the limitation of specific pyrimidines contributed to this phenotype, we next supplemented cultures with individual pyrimidines. Neither deoxycytidine nor thymidine restored proliferation or suppressed differentiation marker expression in AICAr- or brequinar-treated cells (Supplementary Fig. 3C, D). By contrast, we previously showed that uridine supplementation, which bypasses de novo pyrimidine synthesis, rescues differentiation induced by both AICAr and brequinar [4], supporting a model in which disruption of pyrimidine nucleotide balance, rather than depletion of an individual nucleotide, is the primary driver of replication stress – mediated differentiation.

**Fig. 3:**
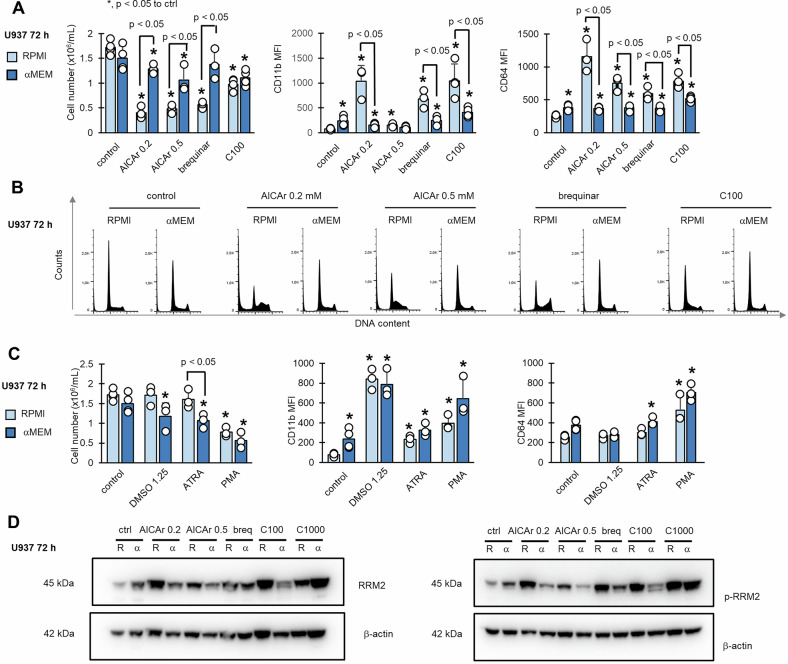
High levels of ribonucleosides and deoxyribonucleosides in αMEM prevents differentiation and modulate RRM2 regulation in U937 cells. **A** U937 cells were cultured for 72 h in RPMI or αMEM medium and treated with AICAr (0.2 or 0.5 mM), brequinar (0.5 µM), or AraC (100 nM). Cell viability and CD11b/CD64 expression were assessed by flow cytometry. Results are expressed as mean ± SD *P < 0.05 vs. control—indicates *P* < 0.05 between experimental groups. **B** Representative histograms of PI-stained cells analyzed by flow cytometry under the conditions described in (**A**). **C** U937 cells were treated with 1.25% DMSO, ATRA (1 µM), or PMA (500 nM) for 72 h. Cell viability and differentiation marker expression were assessed by flow cytometry. *P < 0.05 vs. control—indicates *P* < 0.05 between experimental groups. **D** Western blot analysis of RRM2 and Thr33-phosphorylated RRM2 (p-RRM2) expression in U937 cells after 48 h treatment with AICAr (0.2 or 0.5 mM), brequinar (0.5 µM), or AraC (100 or 1000 nM) in RPMI or αMEM.

High dNTPs can alleviate replication stress [28]. Standard RPMI medium used to culture AML cell lines lacks ribonucleotides and deoxyribonucleotides. Our previous work demonstrated that ribonucleoside addition fully prevented AICAr and brequinar effects but did not affect AraC, reflecting their distinct replication stress mechanisms [9]. To test high-dose deoxyribonucleosides, cells were cultured in αMEM with 10 mg/ml of ribonucleosides and deoxyribonucleosides. In αMEM, the effects of all agents on proliferation, differentiation, and cell cycle arrest were abolished (Fig. 3A, B and Supplementary Fig. 1B). This indicates that abundant extracellular deoxyribonucleosides can replenish intracellular dNTP pools, thereby relieving replication stress not only caused by nucleotide biosynthesis inhibition but also by nucleoside analog incorporation.

Importantly, this was not a general impairment of differentiation capacity, as marker levels remained comparable in response to well-established differentiation inducers like DMSO, ATRA, and phorbol 12-myristate 13-acetate (PMA) (Fig. 3C).

### AICAr, brequinar, and AraC increase RRM2 levels in U937 cells

RNR is an enzyme that catalyzes dNTP formation, with the RRM2 subunit contributing to balanced dNTP levels during S phase [12, 23, 24]. To examine effects on RRM2, U937 cells were treated with two doses of AICAr, brequinar, or cytarabine in RPMI or αMEM supplemented with ribonucleosides and deoxyribonucleosides.

All three agents increased RRM2 expression in U937 cells after 72 h with low-dose AICAr showing a stronger effect than high-dose (Fig. 3D and Supplementary Fig. 4A). In supplemented αMEM, RRM2 increases were less pronounced for all treatments, except for control cells, which showed elevated RRM2. Phosphorylation of RRM2 at Thr33 mirrored the patterns observed for total protein levels.

### Effects of RNR inhibitors on U937 differentiation

To investigate RNR’s role in differentiation, we tested two RRM2 inhibitors, COH29 and hydroxyurea (HU), in U937 cells.

**Fig. 4:**
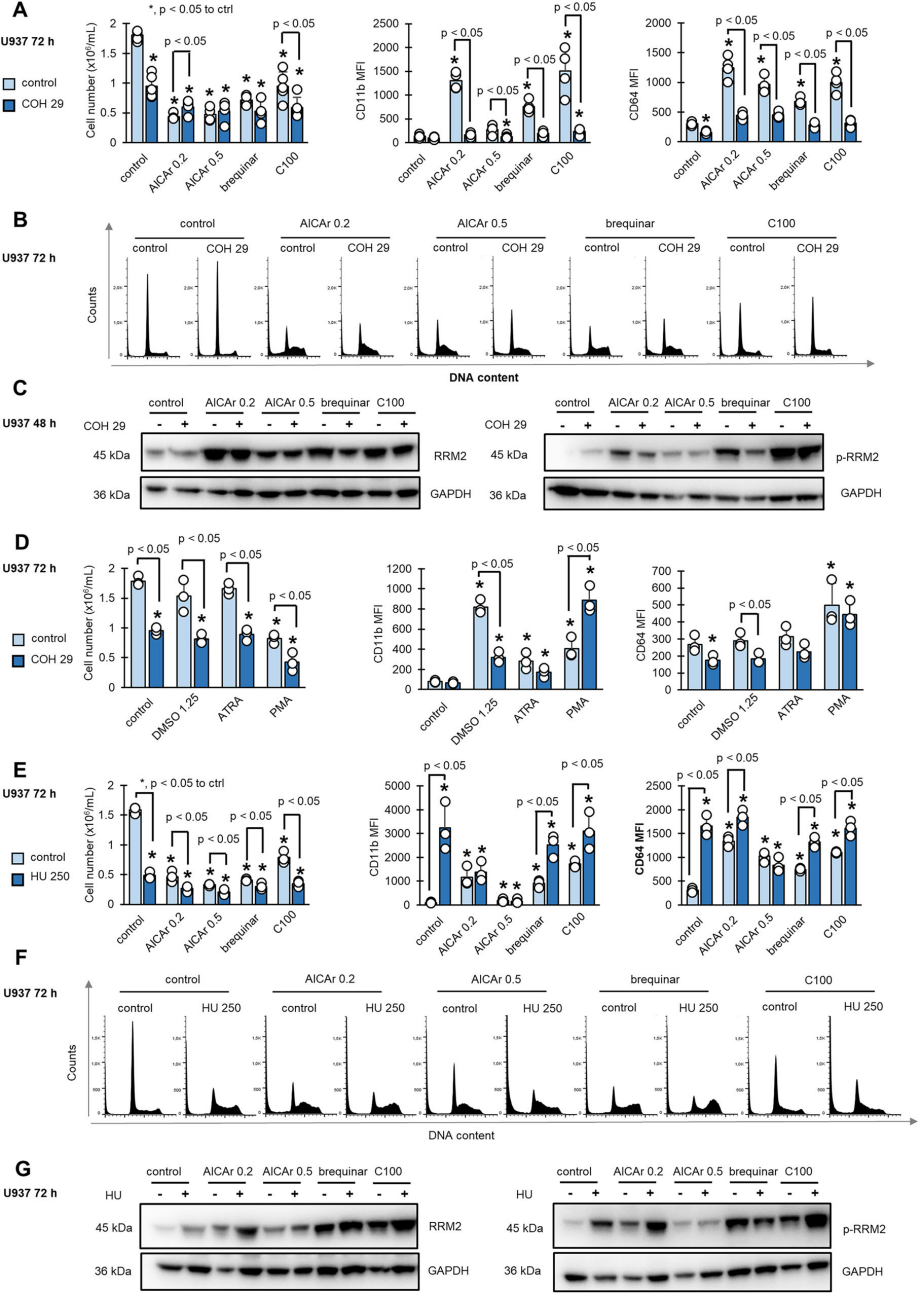
Opposing effects of COH29 and hydroxyurea on leukemia cell differentiation. U937 cells were treated with AICAr (0.2 or 0.5 mM), brequinar (0.5 µM), or AraC (100 nM) in the presence (+) or absence (−) of the RNR inhibitor COH29 (10 μM) for 48–72 h. Cell viability and CD11b/CD64 expression were assessed by flow cytometry (**A**), representative PI-stained histograms are shown in (**B**), and RRM2 expression was analyzed by Western blot (**C**). **D** U937 cells were treated with 1.25% DMSO, ATRA (1 µM), or PMA (500 nM) in the presence or absence of COH29 (10 μM) for 72 h, and cell viability and differentiation marker expression were assessed by flow cytometry. U937 cells were treated with AICAr (0.2 or 0.5 mM), brequinar (0.5 µM), or AraC (100 nM) in the presence (+) or absence (−) of hydroxyurea (HU, 250 μM) for 48–72 h. Cell viability and differentiation marker expression were assessed by flow cytometry (**E**), representative PI-stained histograms are shown in (**F**), and RRM2 expression was analyzed by Western blot (**G**). Results are expressed as mean ± SD *P < 0.05 vs. control— indicates *P* < 0.05 between experimental groups.

COH29, a small-molecule RNR inhibitor reported to block nelarabine-induced differentiation [20], reduced cell viability after 72 h and completely prevented the differentiation marker increases induced by AICAr, brequinar, and low-dose AraC (Fig. 4A), without altering S phase arrest (Fig. 4B and Supplementary Fig. 1C). Western blots showed no significant changes in total or phosphorylated RRM2 levels (Fig. 4C and Supplementary Fig. 4B). COH29’s effects on unrelated agonists were context-dependent: it suppressed CD11b/CD64 with DMSO, had no effect with ATRA, and enhanced CD11b with PMA (Fig. 4D). These results suggest that COH29 does not act as a general inhibitor of differentiation markers and, unlike αMEM, does not function by rescuing nucleotide pools, but rather modulates specific differentiation pathways in a context-dependent manner.

HU, a classical RRM2 inhibitor, was tested at a concentration previously shown to reduce viability by ~50% while inducing differentiation [6]. HU decreased viability (Fig. 4E), caused pronounced S phase arrest (Fig. 4F and Supplementary Fig. 1D), induced differentiation markers, and further enhanced markers with brequinar or AraC, but not with AICAr (Fig. 4E). Western blotting showed increased RRM2 levels in both control and AICAr-treated cells after HU pretreatment (Fig. 4G and Supplementary Fig. 4C).

Given the divergent outcomes of the two RRM2 inhibitors, we next assessed their combined effect. Co-treatment partially restored G2 progression in low-dose HU-treated cells, increased cytotoxicity at high dose, and abolished HU-induced differentiation marker expression (Supplementary Fig. 5A, B). These findings indicate that COH29 interferes with HU-driven differentiation, suggesting mechanistic differences from classical RNR inhibition. To further assess whether these divergent effects reflect compound-specific mechanisms, we tested clofarabine, an RNR-targeting nucleoside analog; clofarabine increased differentiation marker expression in U937 cells under sublethal conditions (Supplementary Fig. 5C).

### Downregulation of RRM2 enhances differentiation induced by pyrimidine synthesis inhibitors without affecting viability

**Fig. 5:**
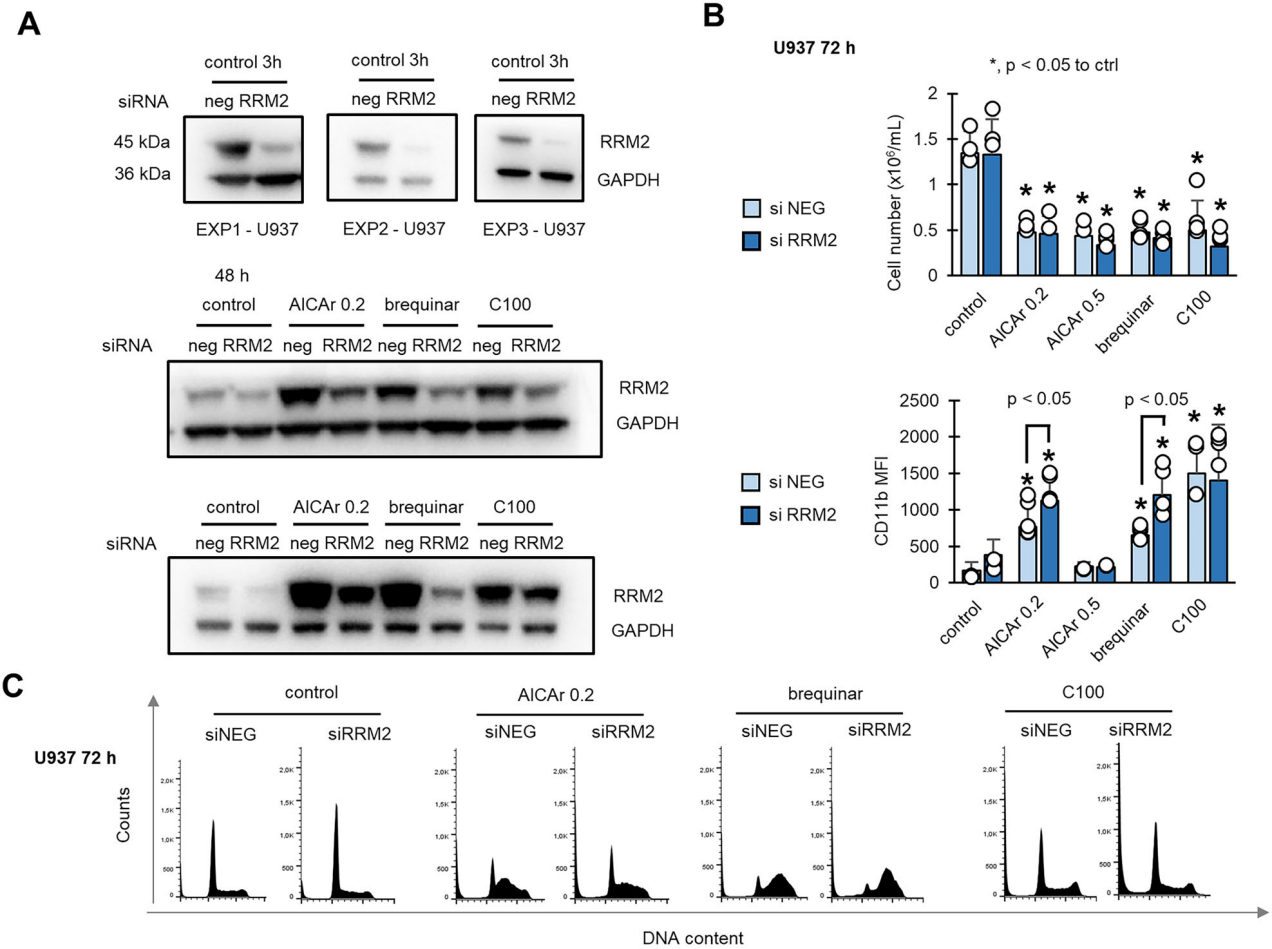
RRM2 knockdown enhances AICAr- and brequinar-induced CD11b expression. U937 cells were transfected with siRNA targeting RRM2 or non-targeting siRNA as a control (neg). After 24 h, cells were treated with AICAr (0.5 mM), brequinar (0.5 μM), or AraC (100 nM). **A** Total cell lysates were collected 3 h or 48 h after treatment and analyzed by Western blot for RRM2 expression. **B** Cell viability and CD11b surface expression were assessed by flow cytometry 72 h after treatment. Results are expressed as mean ± SD *P < 0.05 vs. control—indicates *P* < 0.05 between experimental groups. **C** Representative histograms of PI-stained cells under the conditions described in (**A**).

To directly assess RRM2’s role in differentiation, U937 cells were electroporated with non-targeting control or RRM2-targeting siRNA. Twenty-four hours later, cells were treated for 72 h with AICAr, brequinar, or low-dose AraC. RRM2 protein levels were consistently reduced in siRNA-treated cells at 3 h after plating, across three independent experiments and all treatment conditions (Fig. 5A and Supplementary Fig. 4D).

RRM2 knockdown did not significantly affect cell viability (Fig. 5B). CD11b expression increased in RRM2-silenced cells following low-dose AICAr and brequinar treatment, but not AraC. Cell cycle analysis revealed that the enhanced differentiation was not associated with major alterations in cell cycle progression (Fig. 5C and Supplementary Fig. 1E).

### AML cells show similar nucleotide shifts during differentiation regardless of p53 status and arrest in different phases of S phase

U937 cells are p53-mutant AML cells that rely on ATM/ATR-mediated G2 checkpoints and differentiate primarily during late S/G2 arrest. In contrast, MOLM-13 cells harbor NPM1 and FLT3 mutations, possess functional p53, are more sensitive to AraC, and arrest predominantly in early S phase during differentiation. Previous work demonstrated that while U937 differentiation depends on G2 arrest and Chk1 activity, MOLM-13 cells still differentiate in response to AICAr, brequinar, and AraC without reaching G2 [4, 10].

**Fig. 6:**
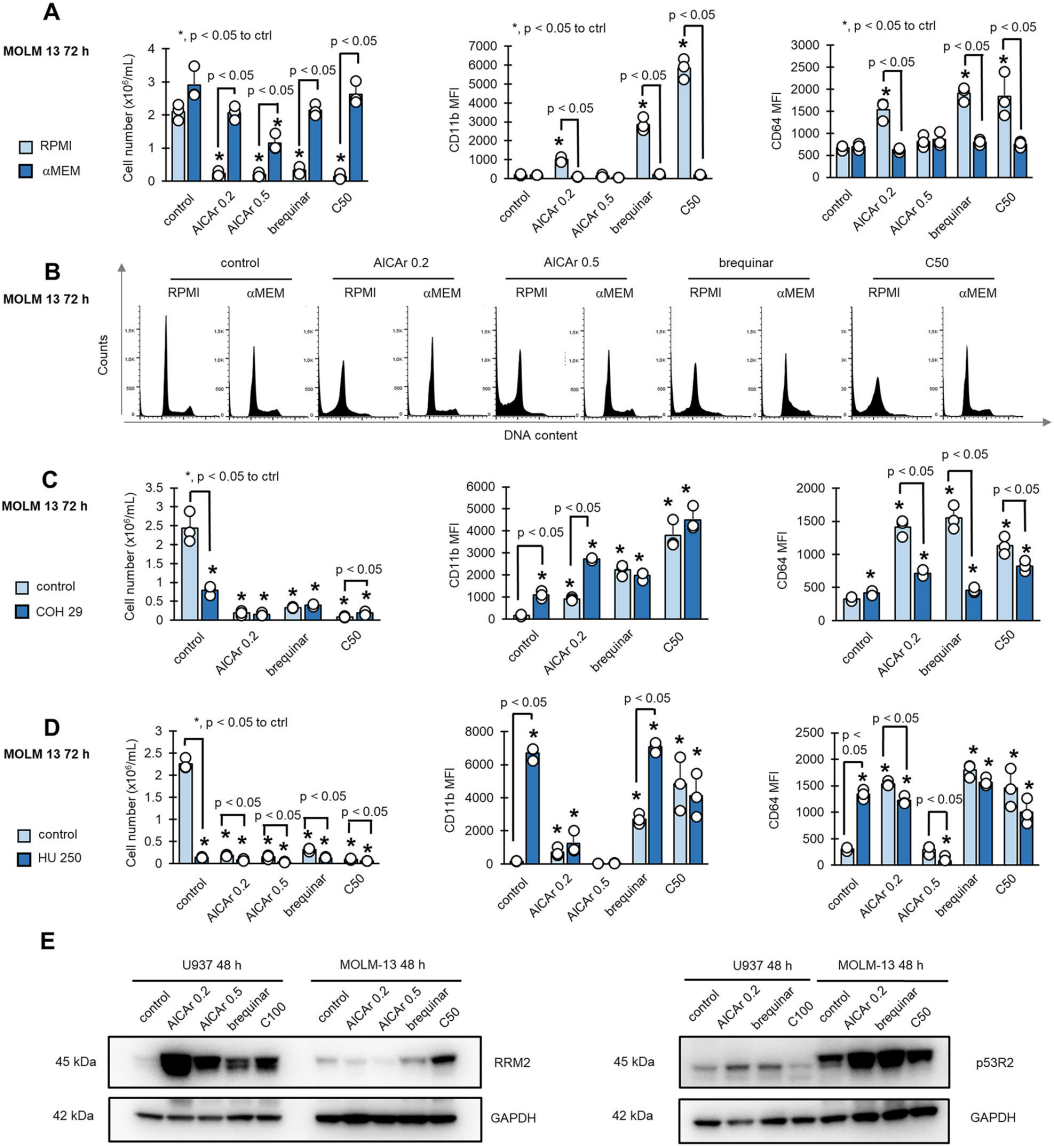
Differentiation agents selectively induce RRM2 and RRM2B/p53R2 in U937 and MOLM-13, and MOLM-13 differentiation is modulated by COH29 and HU. **A** MOLM-13 cells were cultured for 72 h in RPMI or αMEM medium and treated with AICAr (0.2 or 0.5 mM), brequinar (0.5 µM), or AraC (50 nM). Cell viability and differentiation marker expression were assessed by flow cytometry. *P < 0.05 vs. control—indicates *P* < 0.05 between experimental groups. **B** Representative histograms of PI-stained cells under the conditions described in (**A**). **C** MOLM-13 cells were treated with AICAr (0.2 mM), brequinar (0.5 µM), or AraC (50 nM) in the presence (+) or absence (−) of COH29 (10 μM) for 48–72 h. *P < 0.05 vs. control—indicates *P* < 0.05 between experimental groups. **D** MOLM-13 cells were treated with AICAr (0.2 mM), brequinar (0.5 µM), or AraC (50 nM) in the presence (+) or absence (−) of HU (250 μM) for 48–72 h. Cell viability and differentiation marker expression were assessed by flow cytometry. *P < 0.05 vs. control—indicates *P* < 0.05 between experimental groups. **E** U937 and MOLM-13 cells were treated with AICAr (0.2 or 0.5 mM), brequinar (0.5 µM), or AraC (100 nM for U937, 50 nM for MOLM-13) for 48 h. Total cell lysates were collected and analyzed by Western blot for RRM2 and RRM2B/p53R2 expression.

To compare metabolic responses, MOLM-13 cells were treated with two doses of AICAr, brequinar, or 50 nM AraC, followed by LC-MS analysis. Despite arrest at distinct points within S phase, MOLM-13 cells exhibited metabolic changes similar to U937 cells, with comparable alterations in purine and pyrimidine metabolism (Supplementary Fig. 6). Heatmap clustering and nucleotide analysis revealed significant changes in NTP and dNTP levels in response to differentiation agents (Supplementary Fig. 7). When MOLM-13 cells were cultured in αMEM supplemented with high doses of ribonucleosides and deoxyribonucleosides instead of RPMI, the effects of all agents on proliferation, differentiation, and cell cycle arrest were abolished (Fig. 6A, B and Supplementary Fig. 1F).

Thus, we conclude that even without G2 arrest, MOLM-13 cells undergo differentiation via mechanisms involving nucleotide imbalance.

### Effects of RNR inhibitors on MOLM-13 differentiation

To evaluate pharmacological RNR inhibition in MOLM-13 cells, we tested COH29 and hydroxyurea (HU) on viability and differentiation markers. COH29 strongly reduced viable control cells, had little effect on AICAr- or brequinar-treated cells, and slightly increased viability in AraC-treated cells. For differentiation markers, COH29 increased CD11b in control and AICAr-treated cells, while CD64 expression was slightly elevated in controls but decreased in AICAr-, brequinar-, or AraC-treated cells (Fig. 6C).

HU produced a different profile: it strongly reduced viability across all conditions, more than in U937 cells, and robustly increased CD11b and CD64 in controls. HU also enhanced CD11b in low-dose AICAr- and brequinar-treated cells (Fig. 6D). Similarly, clofarabine increased differentiation marker expression while markedly reducing viability in MOLM-13 cells (Supplementary Fig. 8).

Comparing MOLM-13 with U937, differentiation patterns in response to pyrimidine synthesis inhibitors and AraC are largely preserved, but responses to RNR inhibitors diverge. In U937, COH29 blocked differentiation without strong cytotoxicity, while HU enhanced differentiation and S phase arrest. In MOLM-13, COH29 effects were selective, and HU cytotoxicity was pronounced, highlighting cell line–specific sensitivities.

Thus, U937 and MOLM-13 cells share metabolic signatures but differ in RNR inhibitor responses.

### Differential regulation of RRM2 and RRM2B in MOLM-13 and U937 cells

RRM2, the canonical RNR subunit, is expressed mainly during S phase to support DNA replication. RRM2B (p53R2) is a p53-inducible isoform upregulated during stress or DNA damage, maintaining DNA repair and mitochondrial DNA synthesis, particularly in non-cycling cells [23, 25]. MOLM-13 cells are p53 wild-type, so we expected RRM2B induction, whereas U937 cells, lacking functional p53, rely almost exclusively on RRM2. Consistently, AICAr, brequinar, or AraC strongly increased RRM2 in U937 cells, while in MOLM-13, RRM2 was induced only by AraC. Conversely, RRM2B was markedly increased in MOLM-13 cells with all agents, including AICAr and brequinar (Fig. 6E).

### siRNA-mediated downregulation of RRM2, but not RRM2B, impairs differentiation and survival in MOLM-13 cells

**Fig. 7:**
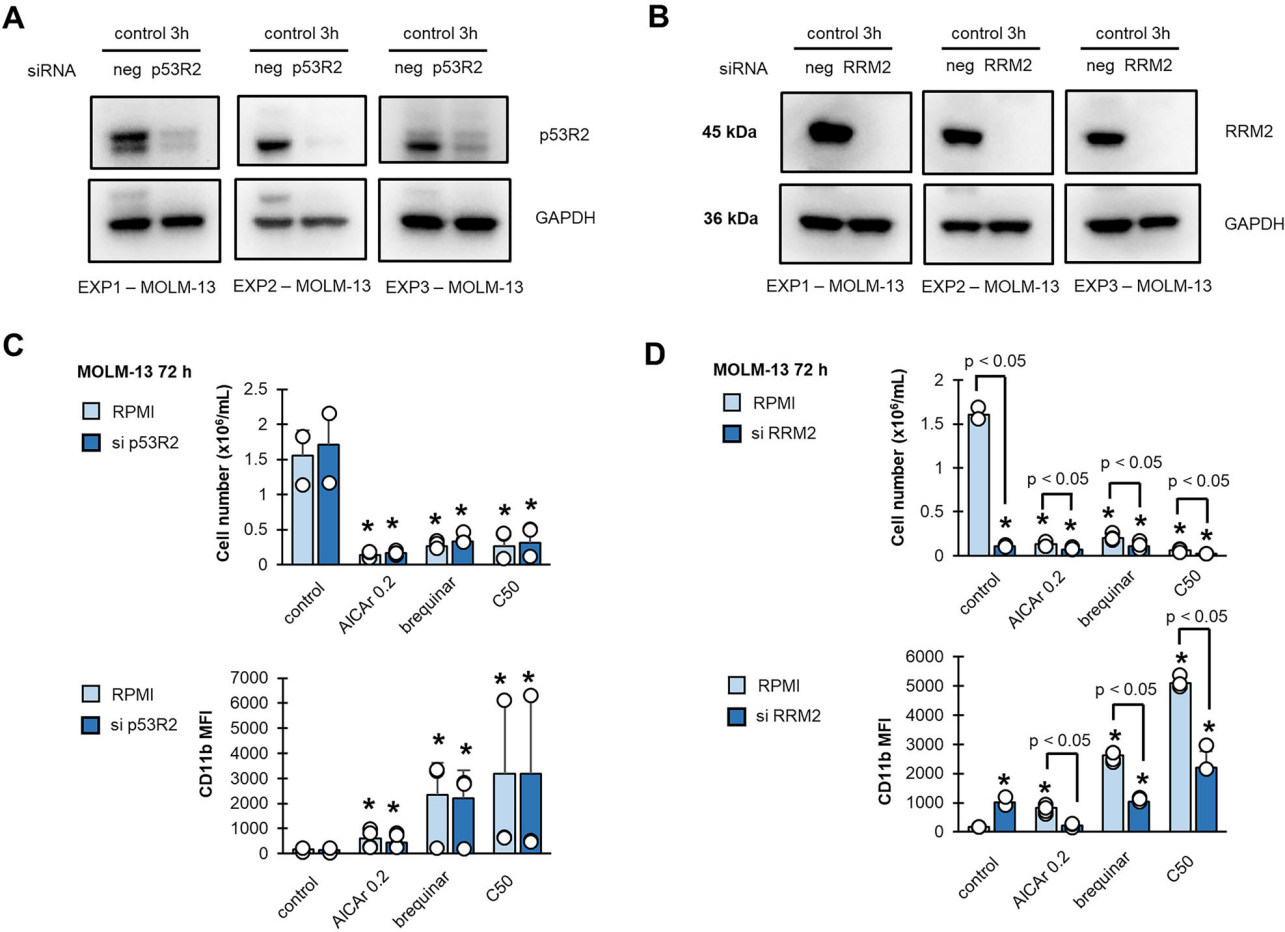
RRM2, but not RRM2B/p53R2, knockdown reduces MOLM-13 cell viability and impairs CD11b expression. MOLM-13 cells were transfected with siRNA targeting p53R2 (RRM2B) (**A**), RRM2 (**B**), or non-targeting siRNA as a control (neg). After 24 h, cells were treated with AICAr (0.2 mM), brequinar (0.5 µM), or AraC (50 nM). Total cell lysates were collected 3 h after treatment and analyzed by Western blot for RRM2B/p53R2 expression (**A**) or RRM2 expression (**B**) (Exp 1–3). **C**, **D** Cell viability and CD11b surface expression were assessed by flow cytometry 72 h after treatment of cells under the conditions described in (**A,** **B**), respectively. Results are expressed as mean ± SD *P < 0.05 vs. control—indicates *P* < 0.05 between experimental groups.

To directly assess the functional contribution of these subunits, we knocked down RRM2B (Fig. 7A) or RRM2 (Fig. 7B) in MOLM-13 cells. Western blot confirmed efficient and specific depletion across three independent experiments (Supplementary Fig. 4F). RRM2B knockdown had no effect on cell viability or differentiation marker expression in control or drug-treated cells (Fig. 7C). In contrast, RRM2 knockdown dramatically reduced viable cells under basal conditions and after treatment with AICAr, brequinar, or AraC. While RRM2 depletion increased CD11b expression in control cells, it attenuated CD11b induction by all differentiation agents (Fig. 7D), suggesting that canonical RRM2 is required for survival and full execution of differentiation programs in MOLM-13 cells.

These findings contrast with U937 cells, where RRM2 knockdown did not reduce viability and enhanced differentiation marker expression with AICAr and brequinar, highlighting that RRM2’s functional role in differentiation is cell type–specific. To determine whether these divergent effects reflected differences in nucleotide availability, we quantified intracellular dNTP levels following RRM2 knockdown. In U937 cells, RRM2 depletion did not reduce dNTP pools at 24 h, but significantly increased dGTP, dATP, and dTTP at 48 h (Supplementary Fig. 9A). In contrast, MOLM-13 cells showed no comparable increases and instead exhibited trends toward reduced dNTP levels (Supplementary Fig. 9B). These findings indicate that RRM2 depletion differentially alters dNTP balance across AML models and suggest that divergent differentiation outcomes reflect differences in nucleotide homeostasis rather than uniform depletion.

### Heterogeneity of RRM2/RRM2B balance in AML cell lines and primary samples

Our previous work demonstrated that differentiation in response to replication stress–inducing agents extends beyond U937 and MOLM-13 cells to additional monocytic AML models, including THP-1 and MOLM-14 [4, 9], whereas more immature myeloid cell lines such as HL-60 and NB4 preferentially undergo cell death rather than differentiation [4, 14]. Additional testing of monocytic AML cell lines revealed that AICAr and brequinar induced differentiation marker expression across multiple models with differing TP53 status, albeit with variable magnitude and effects on viability (Supplementary Fig. 10).

To assess whether the distinct RRM2 and RRM2B regulation observed in U937 and MOLM-13 cells reflects broader patterns across AML models, we examined publicly available global proteomic data from AML cell lines [29]. RRM2/RRM2B ratios exhibited substantial heterogeneity, with U937 and MOLM-13 positioned toward opposite ends of the spectrum and other monocytic AML cell lines, such as THP-1, positioned close to the median (Supplementary Fig. 11A). Comparison of TP53-wild type and TP53-mutant AML cell lines revealed an increase in RRM2/RRM2B ratios in TP53-mutant lines, driven primarily by reduced RRM2B protein levels (Supplementary Fig. 11B). No meaningful correlation was observed between the RRM2/RRM2B ratio and the basal levels of differentiation markers ITGAM, MPO, or FCGR1A in this panel of cell lines (data not shown).

We next asked whether similar relationships could be detected in primary AML samples using an integrated dataset comprising DNA sequencing, proteomics, phosphoproteomics, and drug response profiling [30]. TP53 mutation status as determined by DNA sequencing did not segregate RRM2/RRM2B ratios, while phosphorylation of CHEK1 (Thr279) correlated with RRM2 abundance. Again, no strong or consistent correlation was observed between RRM isoform abundance and baseline ITGAM and MPO expression, with only a modest correlation observed for FCGR1. Sensitivity to DHODH inhibition, quantified as drug sensitivity score (DSS) and reflecting cytotoxic response rather than differentiation induction, was inversely associated with ITGAM expression, indicating reduced susceptibility of more CD11b-positive samples to DHODH-mediated killing (Supplementary Table 1).

Together, these findings suggest that replication stress–induced differentiation can occur across both TP53-mutant and TP53-wild-type AML cell lines, and highlight substantial heterogeneity in baseline RRM2/RRM2B balance across AML models.

## Discussion

In this study, we performed the first extensive metabolomic analysis in AML cells in response to AICAr and compared it with other agents that induce S-phase arrest and differentiation. As expected, brequinar selectively inhibited DHODH, whereas AICAr profoundly affected both purine and pyrimidine de novo synthesis. While effects on purine metabolism increased with dose, the effects on UMP synthesis were biphasic and correlated better with differentiation. Notably, high-dose AICAr caused accumulation of thymine and thymidine, which may explain the reduced S/G2 arrest and lower induction of differentiation at this dose. Thymidine accumulation aligns with its role in synchronizing cells at G1/S (“double thymidine block”) and may reflect activation of a salvage pathway, converting thymine from DNA turnover into thymidine when de novo dUMP-to-dTTP conversion is limited. This pathway can both induce and alleviate replication stress in hematopoiesis, and endogenous thymidine can trigger RS in vivo [2, 12]. Since brequinar reduces UMP without thymidine accumulation, concurrent purine changes appear necessary to trigger this salvage response.

Another factor that may help explain the biphasic effects of AICAr is its impact on purine nucleotides, particularly AMP and GMP, a phenomenon first described in fibroblasts in 1981 [31]. At high doses, AICAr not only affects pyrimidine pools but also reduces purine NTPs and dNTPs. This suggests that cell differentiation may be more sensitive to imbalances among nucleotide pools than to absolute concentrations: when pyrimidines are limiting relative to purines, differentiation is favored, whereas a concomitant reduction in purines diminishes it. Consistently, supplementation with dA and dG in brequinar-treated cells further exacerbates the purine/pyrimidine imbalance, supporting the notion that relative imbalance, rather than absolute nucleotide levels, is a key determinant of S phase progression and enhanced differentiation. Recent work indicates such imbalances are sensed via replication stress signaling rather than canonical growth pathways [32]. This aligns with our observation that low-dose AICAr, which creates stronger pyrimidine imbalances without excessively lowering purines, correlates with S/G2 phase arrest and differentiation, whereas high dose partially rescues S phase progression and reduces differentiation.

We initially focused on RRM2 because culturing cells with supraphysiological doses of ribonucleosides and deoxyribonucleosides abolished S-phase arrest and differentiation in response to all agents, including cytarabine. Cytarabine has been reported to increase RNR activity and dNTP levels [20], which we also observed. Our study confirmed that cytarabine induces RRM2 upregulation, with similar increases in AICAr- and brequinar-treated cells, largely prevented in αMEM. Notably, RNR upregulation occurs despite reduced intracellular pyrimidine dNTP levels in AICAr- and brequinar-treated cells, suggesting a compensatory response to replication stress. Cells appear to increase RRM2 in an attempt to restore dNTP pools and sustain DNA synthesis. Nevertheless, under conditions of DHODH or UMP synthase inhibition, this compensatory response is insufficient to fully normalize nucleotide pools, allowing replication stress to persist and drive differentiation. These observations highlight that while RNR upregulation is a protective response, its effectiveness depends on both the availability of precursors and the cellular context. High exogenous dNTPs could either directly inhibit RRM2 via feedback or allow cells to bypass pyrimidine limitation or AraC competition, enabling S phase progression without activating DNA damage signaling. In both scenarios, RRM2 upregulation is abrogated; however, this does not imply that differentiation in response to our agents strictly depends on relative nucleotide pool imbalance or RNR activity. Rather, these findings, combined with cell cycle analysis, indicate that high nucleoside concentrations can rescue cells from replication stress, S-phase arrest, RRM2 induction, and differentiation induced by these agents.

The effects of pharmacological RNR inhibitors differed markedly between the two cell lines. Notably, combined treatment in U937 cells inhibited, rather than enhanced, HU-induced differentiation. COH29, a recently discovered RNR inhibitor that prevents holoenzyme assembly by binding RRM2’s C-terminal interface [33], completely abolished CD11b expression in response to all agents, similar to previous reports for nelarabine [20]. HU, a well-established cytostatic agent, induces differentiation in parallel with S phase arrest [6], effects we confirmed in control cells of both cell lines. The reduction in viable cell number was greater in MOLM-13 than in U937 cells, mirroring siRRM2 effects and supporting convergence of HU and genetic RRM2 inhibition on similar differentiation responses. COH29 moderately increased CD11b expression in control cells, especially in MOLM-13, but this occurred without S phase arrest, highlighting a mechanistic difference from HU and siRRM2. Consistent with this interpretation, clofarabine more closely recapitulated the differentiation-associated effects observed with HU and siRNA-mediated RRM2 depletion than those seen with COH29, helping to reconcile the apparent discrepancy between pharmacological and genetic RRM2 perturbation. This suggests that COH29 exerts mechanistic effects distinct from classical RNR inhibition in these cell lines.

Some differences remain between hydroxyurea and siRRM2 downregulation. HU induced pronounced S phase arrest, whereas siRNA-mediated RRM2 knockdown has a more limited impact on cell cycle progression, consistent with other models [34]. While dNTPs were not measured in HU-treated cells here, prior studies show HU depletes and/or imbalances dNTPs in hematopoietic cells, causing S phase accumulation and increased CD11b expression in ER-Hoxa9 cells [6]. In THP-1 cells, HU selectively lowers purine dNTPs and alters the dCTP/dATP ratio, enhancing AraC toxicity by suppressing SAMHD1 ara-CTPase activity [35]. These findings suggest HU primarily promotes differentiation through dNTP perturbation, reflected in the consistent CD11b increase seen with control, AraC-, and brequinar-treated cells. The exception was when HU was combined with AICAr: although HU still induces S phase arrest, it does not further enhance CD11b expression, likely because AICAr’s purine-driven nucleotide imbalance limits HU-induced differentiation. In addition to dNTP depletion, HU also generates ROS that impair DNA polymerases [36], providing an additional mechanism by which HU affects DNA replication and the cell cycle. In nonhematopoietic cells, a similar purine-specific reduction occurs following siRNA-mediated RRM2 knockdown [37, 38]. However, inducible RRM2 suppression in U937 cells did not produce uniform depletion of dNTP pools [20], consistent with our finding that dNTP levels were unchanged at 24 h but selectively increased (dATP, dGTP, and dTTP) at later time points following RRM2 knockdown. This supports the concept that differentiation is driven by disruption of dNTP balance rather than absolute depletion.

RNR activity is closely linked to the cell cycle through RRM2, which peaks at G1/S and is degraded in G2/M, while DNA damage signaling via ATR/ATM–Chk1 promotes its accumulation [23, 34, 39]. Our previous work showed that both U937 and MOLM-13 cell lines activate Chk1 and undergo differentiation in response to replication stress, though their cell cycle responses differ [10]. In this study, U937 (p53-mutant) progresses to G2 with strong RRM2 induction and Thr33 phosphorylation, whereas MOLM-13 (p53-wild-type) arrests in early S with detectable RRM2 but no significant increase or Thr33 phosphorylation. p53R2 induction depended on functional p53 and was observed only in MOLM-13 cells treated with all agents.

Despite the absence of RRM2 upregulation in MOLM-13 cells, RRM2 knockdown profoundly reduced viability and abolished agent-induced differentiation, whereas p53R2 knockout had no effect. This aligns with findings in colon cancer, where p53(+/+) cells show greater sensitivity to DNA-damaging agents than p53(−/−) cells, despite similar RRM2 and p53R2 patterns, suggesting p53R2 alone is insufficient for effective DNA repair [37].

Silencing RRM2 in various models induces S-phase block, γ-H2Ax accumulation, and Chk1 activation [34, 40], and HU activates Chk1 in U937 cells [41]. Our previous work showed Chk1 contributes to differentiation induced by pyrimidine synthesis inhibitors in U937 [4]. Here, RRM2 downregulation enhanced differentiation in untreated and pyrimidine inhibitor-treated U937 cells, likely by aggravating dNTP imbalance and replication stress without causing substantial cell death. Consistent with this interpretation, RRM2 knockdown increased selected dNTPs in U937 cells at later time points, supporting a role for nucleotide imbalance in differentiation.

An important translational consideration is that DHODH and RNR are essential components of nucleotide metabolism, raising questions regarding the therapeutic window. However, brequinar has been shown to preferentially induce differentiation and growth arrest in AML cells while exhibiting substantially lower activity in normal hematopoietic progenitors, supporting the existence of a potential therapeutic window [42]. Moreover, both DHODH inhibitors and acadesine (AICAr) have been evaluated clinically, demonstrating the feasibility of systemic modulation of these pathways under defined dosing regimens [42, 43]. RNR inhibition likewise has clinical precedent, as exemplified by hydroxyurea, which is widely used with dose-limiting but manageable myelosuppression [44].

In summary, our study demonstrates that AML cell differentiation in response to AICAr, brequinar, and cytarabine is tightly coupled to nucleotide metabolism and clarifies the role of RNR in replication stress-mediated differentiation. Both U937 and MOLM-13 differentiate under replication stress, yet RNR plays opposite roles in shaping this response. In U937, strong ATR–CHK1 activation and RRM2 upregulation normally buffer nucleotide imbalance and dampen the differentiation trigger, whereas RRM2 knockdown removes this buffer and amplifies differentiation. In contrast, MOLM-13 cells display limited RRM2 induction and predominant upregulation of p53R2 (RRM2B). Because p53R2 provides only small, repair-oriented dNTP pools insufficient for bulk S-phase DNA synthesis, MOLM-13 cells operate near the minimal replication threshold. Further RRM2 loss therefore drives fork collapse and prevents differentiation, despite the presence of replication stress. Thus, while replication stress initiates differentiation, the balance between RNR subunits and checkpoint competence determines RNR’s role in survival versus differentiation. These findings suggest that targeting nucleotide metabolism, combined with strategies that reinforce checkpoint signaling, could optimize differentiation therapy in AML.

## Materials and methods

### Reagents and resources

All antibodies, chemicals, siRNAs, and cell culture reagents used in this study are listed in Supplementary Table 2.

### Cell culture

Human acute myeloid leukemia (AML) cell lines U937, MOLM-13, THP-1, MV-411 and OCI-AML3 were cultured in RPMI-1640 medium supplemented with 10% fetal bovine serum, 2 mM L-glutamine, 50 U/mL penicillin, and 50 μg/mL streptomycin. Cells were maintained at 37 °C in a humidified incubator with 5% CO₂. For experiments, exponentially growing cells were seeded at 0.2 × 10⁶/mL in six-well plates or 0.3 × 10⁶/mL in 25 cm² flasks. Treatments were applied as indicated in the figure legends. Viable cell numbers were determined at the indicated time points using trypan blue exclusion and a hemocytometer.

### Immunophenotyping

After incubation, U937, MOLM-13, THP-1, MV-411 and OCI-AML3 cells were collected, washed, and incubated with Fc Receptor Blocking solution for 10 min. Cells were stained with anti-CD11b-FITC, anti-CD64-FITC, or corresponding isotype controls for 20 min at room temperature in the dark. Non-viable cells were excluded by forward/side scatter (FSC/SSC) gating and 7-AAD staining. Doublets were excluded by FSC-H versus FSC-A gating. Flow cytometry was performed on a FACSCanto II (BD Biosciences, San Jose, CA), and data were analyzed using FlowJo_v10.8.1 (Tree Star Inc., Ashland, OR, USA). Mean fluorescence intensity (MFI) was calculated by subtracting the signal of isotype controls from that of antibody-stained samples.

### Cell cycle analysis

Cells were washed and stained with propidium iodide (PI) buffer (50 μg/mL PI, 10 mM Tris pH 8.0, 10 mM NaCl, 10 μg/mL RNase A, 0.1% Igepal) and incubated for 20 min at 4 °C. Data were collected on a FACSCanto II, acquiring 10,000 events per sample. Aggregates and debris were excluded as previously described [10].

Cell cycle phase distribution (G0/G1, S, and G2/M) was determined from PI DNA content histograms using a consistent gating and modeling strategy across all experiments in FlowJo. Quantification represents the percentage of cells in each cell cycle phase and is shown as mean ± SD from at least three independent experiments. Representative histograms are shown in the figures.

### Western blotting

Exponentially growing U937 or MOLM-13 cells (0.3 × 10⁶/mL, 25 cm² flasks) were treated as indicated, harvested, and lysed in cell lysis buffer containing 1 mM PMSF and 1 μM microcystin. Lysates were sheared through a 23-gauge needle (7 passes), incubated on ice for 10 min, and centrifuged at 14,000 × *g* for 10 min at 4 °C. Supernatants were collected and stored at −80 °C. Protein concentrations were measured by Bradford assay. Equal protein amounts (50 μg/lane) were separated on 4–12% SDS-PAGE gels, transferred to PVDF membranes, and blocked in TBST with 5% non-fat dry milk for 30 min. Membranes were incubated overnight at 4 °C with primary antibodies against RRM2, P-RRM2 (T33), p53R2, or GAPDH (1:1000 in TBST/5% BSA), washed, and incubated with HRP-conjugated secondary antibodies (1:2000, 2 h, room temperature). Bands were visualized with SuperSignal™ West Pico PLUS substrate and imaged using a ChemiDoc™ MP Imaging System (Bio-Rad). Densitometric quantification was performed using Adobe Photoshop CS6 (Adobe Systems Inc., San Jose, CA, USA). Protein signals were normalized to the corresponding loading control and expressed relative to control conditions. Data shown represent mean ± SD from at least three independent experiments.

### siRNA transfection

Knockdown of RRM2 and RRM2B was achieved using siRNA transfection with Neon™ Transfection System (Thermo Fisher Scientific, Waltham, MA, USA), as described previously [4, 9, 45]. Cells were washed, resuspended in transfection buffer (23 × 10⁶/mL), and electroporated with siRNA (final concentration 130 nM; siRNA volume ≤ 14% of total). For U937 cells, a single pulse at 1050 V/50 ms was applied; for MOLM-13, a single pulse at 1700 V/20 ms. Following electroporation, cells were cultured in antibiotic-free medium for ≥16 h at 37 °C, then replated for differentiation and cell cycle assays. Protein knockdown was verified by western blotting.

### Metabolomic analysis

Cells were harvested, counted, and polar metabolites were extracted using a mixture of 40% acetonitrile (MeCN), 40% methanol (MeOH), and 20% water (H₂O) [46]. After vortexing for 15 s, the samples were incubated at −20 °C for 20 min. The samples were then centrifuged at maximum speed, and the supernatant was transferred to HPLC vials. Polar metabolites were analyzed using liquid chromatography coupled to tandem mass spectrometry (LC–MS/MS), following the methodology described by Yuan et al. [47]. Separation was performed on an XBridge Amide column (100 mm × 2.1 mm, 4.6 µm; Waters, Manchester, UK) using a Nexera UHPLC system (Shimadzu, Kyoto, Japan) with gradient elution. Solvent additives for HILIC high-pH separation consisted of ammonium acetate and 0.1% ammonium hydroxide. Metabolite detection was carried out on a SCIEX QTRAP 6500 triple quadrupole mass spectrometer (Applied Biosystems, Framingham, MA, USA) operated in both positive and negative ionization modes. Raw data files were imported into Skyline software for peak integration, and subsequent data normalization and statistical analyses were performed using the online tool MetaboAnalyst. Metabolite peak areas were normalized to cell number (per 10⁶ cells) prior to analysis; for simplicity, normalized values are referred to as ‘Peak area’ throughout the figures.

### Proteomics data from AML cell lines

Publicly available global proteomic data for human leukemia cell lines were obtained from a previously published mass spectrometry–based study [29]. The dataset contains unimputed protein abundance values reported as log₂-transformed intensities, which were used directly for all analyses. Protein abundance data for RRM2, RRM2B, and differentiation-associated markers (MPO, ITGAM/CD11b, and FCGR1A/CD64) were extracted.

For each cell line, values from technical replicates (up to four per cell line) were summarized as the median log₂ protein abundance. The log₂(RRM2/RRM2B) ratio was calculated as the difference between median log₂-transformed RRM2 and RRM2B values. Analyses were performed using both an unfiltered dataset including all cell lines with available measurements and a quality-controlled dataset restricted to cell lines with ≥ 3 valid technical replicates for both proteins.

TP53 mutation status was obtained from the Cellosaurus database [48] and verified using DepMap Public 25Q3 dataset² (Broad Institute, 2025) [49] when necessary. Cell lines were classified as TP53-wildtype or TP53-mutated based on the presence of pathogenic TP53 coding mutations.

### Proteomics, drug response, and genomic data from primary AML samples

Proteomic abundance data, ex vivo drug sensitivity scores (DSS), and DNA sequencing–based mutation data for primary AML samples were obtained from a previously published study [30]. Proteomic data were generated by mass spectrometry–based global proteomic profiling and are reported as log₂-transformed protein intensities.

DSS values for DHODH inhibitors and cytarabine were used as provided by the original study. TP53 mutation status was determined from DNA sequencing data and samples were classified as TP53-wildtype or TP53-mutant accordingly. Protein abundance values for RRM1, RRM2, RRM2B, CHEK1 (Thr279 phosphorylation), and differentiation markers (MPO, ITGAM/CD11b, FCGR1/CD64) were extracted for downstream analyses.

Associations between basal RRM isoform abundance, RRM2/RRM2B ratios, and differentiation marker expression were assessed using Spearman’s rank correlation. Group comparisons of log₂(RRM2/RRM2B) ratios between TP53-wildtype and TP53-mutant AML cell lines were performed using a two-sided Mann–Whitney U test.

### Statistical analyses

Statistical analyses of proteomic data from AML cell lines and primary AML samples were performed using GraphPad Prism (version X). Associations between continuous variables were assessed using Spearman’s rank correlation. Associations between binary variables (TP53 status) and continuous molecular features were assessed using point-biserial correlation. Group comparisons between TP53-wildtype and TP53-mutant samples were performed using two-sided Mann–Whitney U tests. P-values were not adjusted for multiple testing for exploratory omics analyses.

Statistical analyses for metabolomics were performed using R (R Foundation for Statistical Computing, Vienna, Austria), MetaboAnalyst 6.0 (Xia Lab, McGill University, Montreal, QC, Canada), Metabolite Autoplotter (10.1186/s40170-020-00220-x), and Microsoft Excel 2016 (Microsoft Corporation, Redmond, WA, USA). Data normalization, log transformation, and statistical tests such as principal component analysis (PCA), hierarchical clustering, and one-way ANOVA with Tukey’s post hoc test were applied to assess differences between experimental groups. For comparisons between two groups, normalized metabolite peak areas were log10-transformed, and statistical significance was assessed using an unpaired two-tailed Welch’s t-test.

All other statistical analyses were performed using Microsoft Excel, with data expressed as mean ± SD from ≥ 3 independent experiments. Comparisons were made using unpaired two-tailed Student’s *t* tests, and *P* values < 0.05 were considered statistically significant.

### Ethics

This study used established human cell lines and publicly available, previously published datasets. It did not involve direct use of human participants, patient-derived material, or animal subjects. Therefore, ethical approval and informed consent were not required.

## Supplementary information

Supplementary information

Supplementary Data 1 Metabolomics data - Independent Experiment 1

Supplementary Data 2 Metabolomics data - Independent Experiment 2

Supplementary Data 3 Metabolomics data - Independent Experiment 3

original western blots

## Data Availability

All metabolomics data generated in this study are included in Supplementary Data 1–3, with each table corresponding to one independent experiment; rows represent individual samples (technical replicates) and columns represent metabolites (values are normalized peak areas per 10⁶ cells). Other supporting data are available from the corresponding author upon reasonable request. Raw metabolomics data can also be provided upon request or deposited in a public repository if required.
