# Supplementary material for: Roles of RRM2 and RRM2B in pyrimidine stress responses and differentiation of acute myeloid leukemia cells
Source: Cell Death Discov. 2026 Apr 24;12:271. doi: 10.1038/s41420-026-03105-y (PMC13243657; doi:10.1038/s41420-026-03105-y)
Supplement: Supplementary file 1 — Supplementary information [file 41420_2026_3105_MOESM1_ESM.pdf]

Supplementary Information

Roles of RRM2 and RRM2B in pyrimidine stress responses and differentiation of acute myeloid leukemia cells

Alojzija Brcic, Hrvoje Lalic, Tomislav Smoljo, Klara Bardač, Vilma Dembitz, Romana Penker, Giovanny Rodriguez Blanco, Antonio Bedalov, Dora Visnjic

This file contains Supplementary Figures S1–S11 and Supplementary Tables S1–S2.

Supplementary Figure 1.

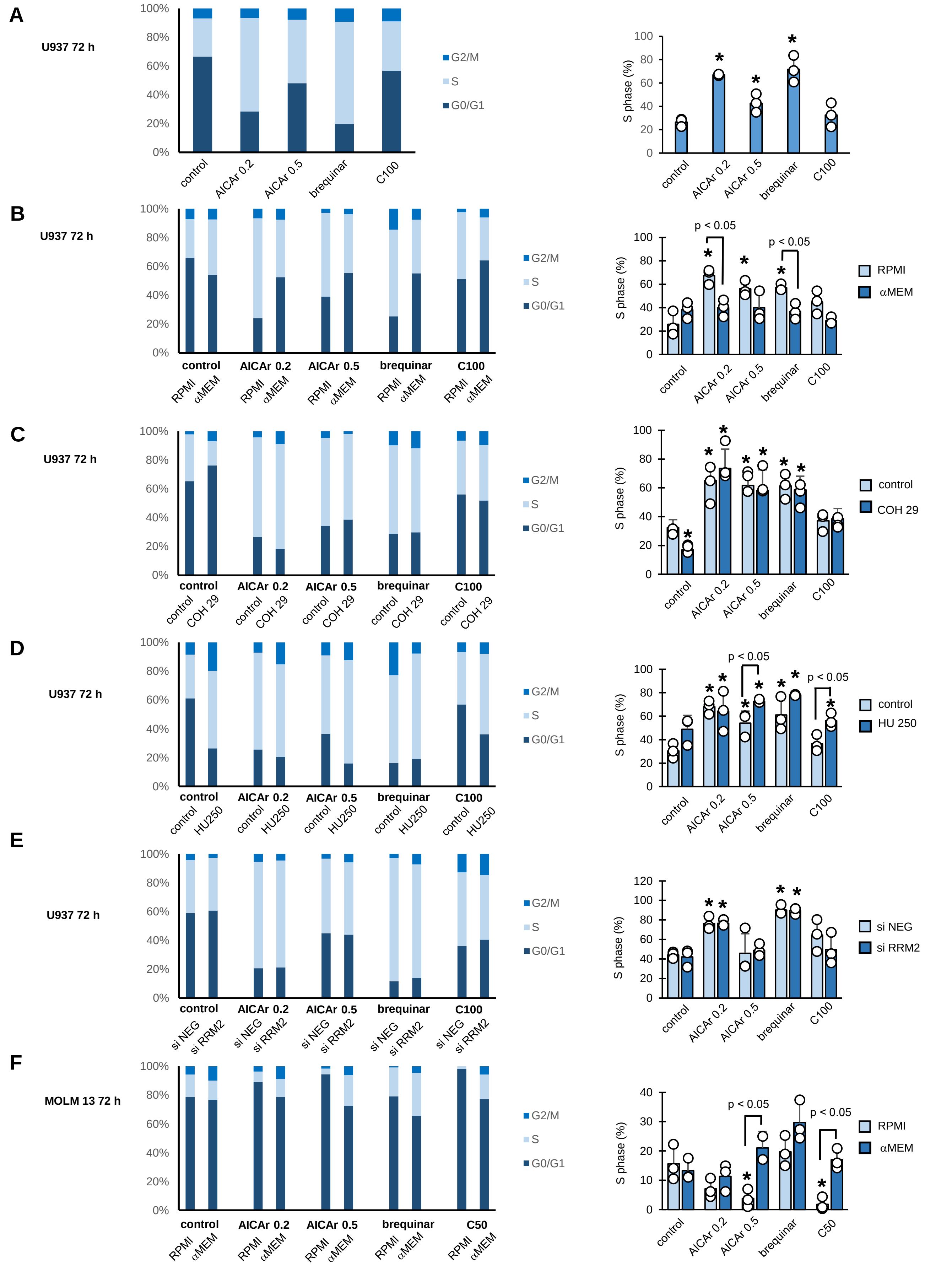

Supplementary Figure 1. Quantification of cell cycle phase distribution corresponding to Figures 1 and 3-6. Cell cycle phases were quantified from PI-stained DNA content histograms using a consistent gating and modeling strategy. Bar plots represent mean ± SD from three independent experiments. **(A)** Quantification corresponding to **Figure 1D** **(B)** Quantification corresponding to **Figure 3B** **(C)** Quantification corresponding to **Figure 4B** **(D)** Quantification corresponding to **Figure 4F** **(E)** Quantification corresponding to **Figure 5C** **(F)** Quantification corresponding to **Figure 6B**. *$P<0.05$ vs control

Supplementary Figure 2

A

U937

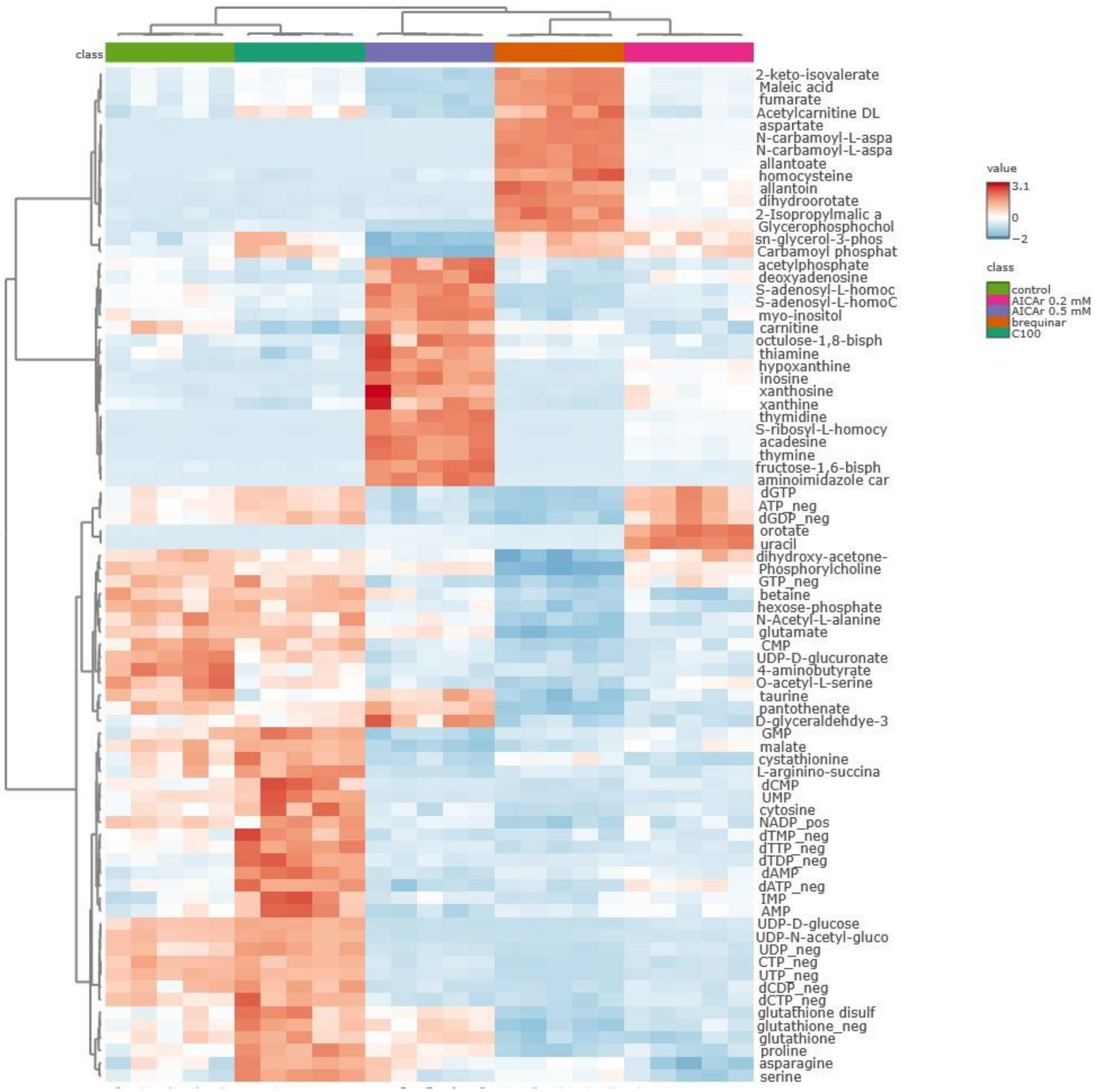

B

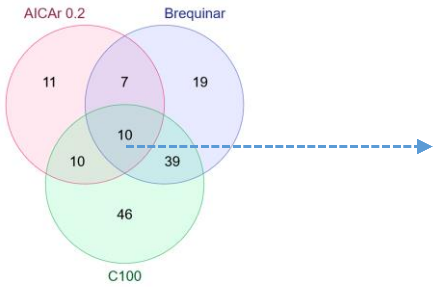

Carbamoyl phosphate
CTP
dCDP
dCMP
dCTP
dGDP
dTTP
hexose-phosphate
sn-glycerol-3-phosphate
UMP

Supplementary Figure 2. Heatmap clustering and Venn diagram analysis of metabolite changes in U937 cells treated with AICAr, brequinar, and AraC.

(A) Heatmap showing hierarchical clustering of metabolite profiles across five treatment groups: control, AICAr (0.2 mM), AICAr (0.5 mM), brequinar (0.5 µM), and 100 nM AraC (C100).

(B) Venn diagram illustrating overlap and unique metabolites significantly altered by AICAr (0.2 mM), brequinar (0.5 µM), and 100 nM AraC (C100).

Supplementary Figure 3

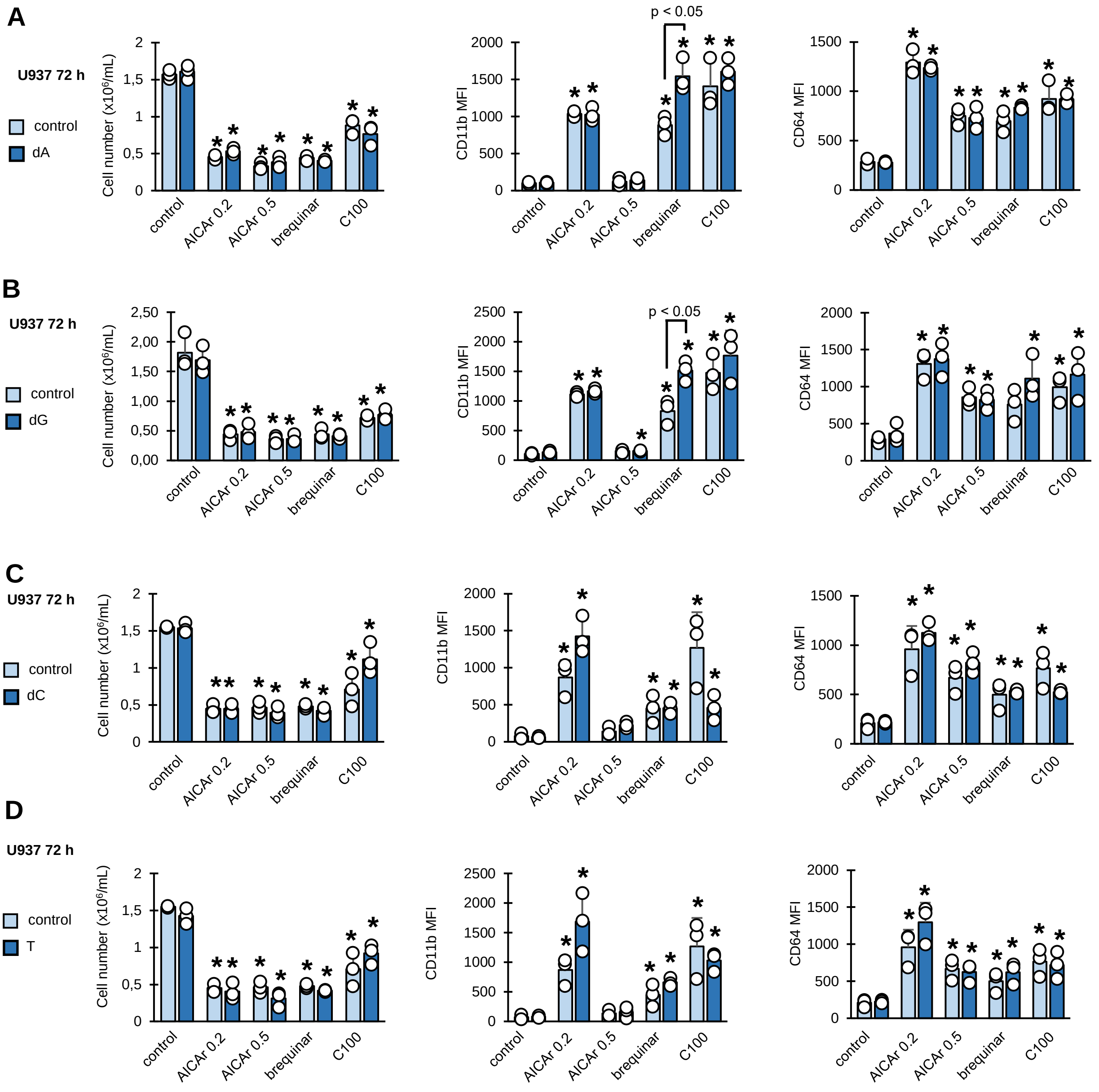

Supplementary Figure 3. Deoxyadenosine and deoxyguanosine potentiate brequinar-induced CD11b expression in U937 cells.

U937 cells were treated with AICAr (0.2 or 0.5 mM), brequinar (0.5 µM), or 100 nM AraC (C100) in the presence (+) or absence (−) of (A) 10 µM deoxyadenosine (dA), (B) 10 µM deoxyguanosine (dG), (C) 10 µM deoxycytidine (dC), or (D) 10 µM thymidine (T). Cell viability and CD11b/CD64 expression were assessed 72 h after treatment. Data represent mean ± SD of at least three independent experiments. *$P < 0.05$ vs. untreated control.

Supplementary Figure 4.

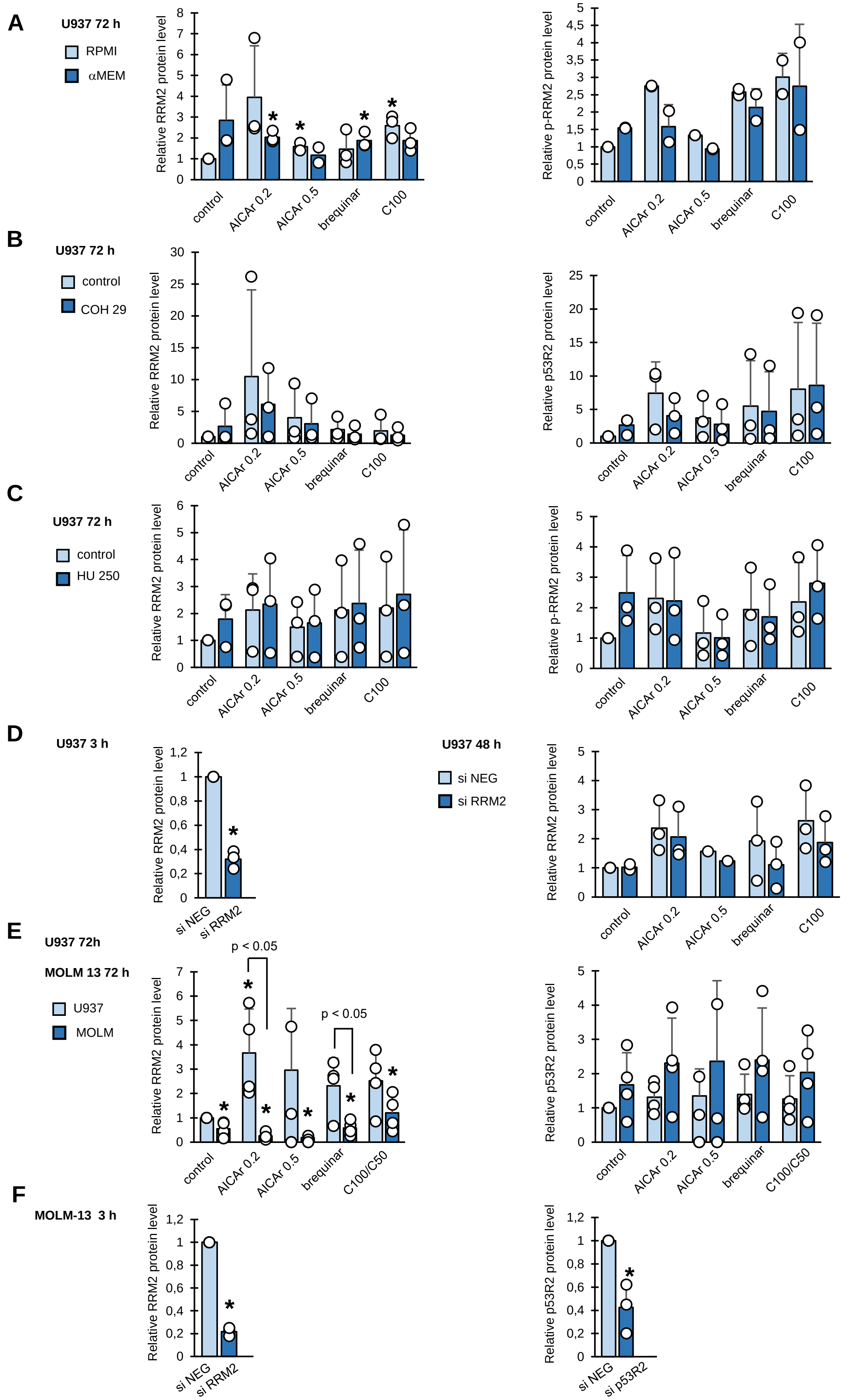

Supplementary Figure 4. Quantification of Western blot data corresponding to Figures 3–7. Western blot bands were quantified by densitometric analysis. RRM2, phospho-RRM2 (Thr33), and RRM2B (p53R2) signals were normalized to the corresponding loading control and expressed relative to the control condition. Data are shown as mean ± SD from three independent experiments. (**A**) quantification corresponding to **Figure 3D**. (**B**) quantification corresponding to **Figure 4C**. (**C**) quantification corresponding to **Figure 4G**. (**D**) quantification corresponding to **Figure 5A**. (**E**) quantification corresponding to Figure **6E**. (**F**) quantification corresponding to **Figure 7A**. **P* < 0.05 vs control.

Supplementary Figure 5

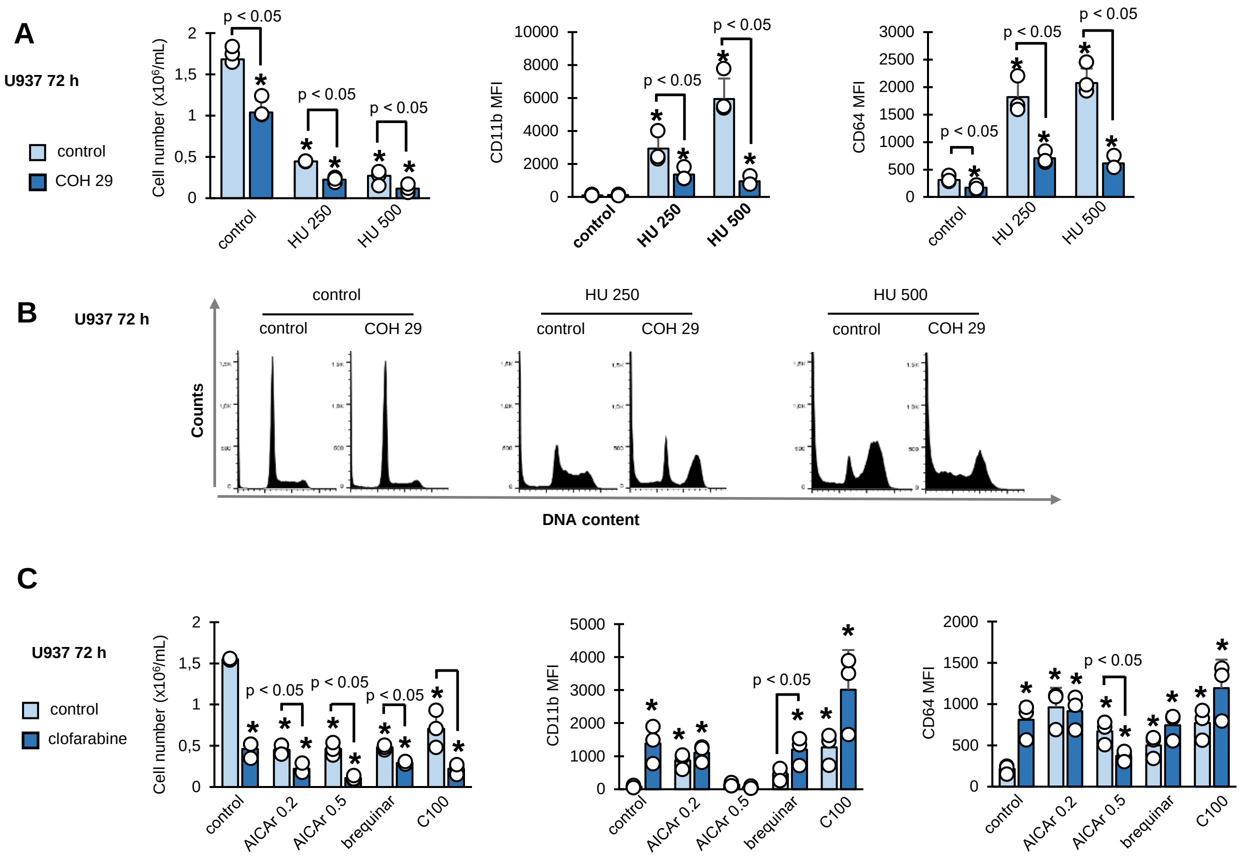

Supplementary Figure 5. COH29 inhibits hydroxyurea-induced differentiation in U937 cells, whereas clofarabine induces differentiation similarly to hydroxyurea.

(A) U937 cells were treated with hydroxyurea (HU; 250 or 500 μM) in the presence (+) or absence (−) of COH29 (10 μM) for 72 h. Cell viability and CD11b/CD64 expression were assessed by flow cytometry.

(B) Representative histograms of PI-stained cells under the conditions described in (A).

(C) U937 cells were treated with AICAr (0.2 or 0.5 mM), brequinar (0.5 μM), or 100 nM AraC (C100) in the presence (+) or absence (−) of 30 nM clofarabine. Cell viability and CD11b/CD64 expression were assessed 72 h after treatment. Data represent mean ± SD of at least three independent experiments. *$P < 0.05$ vs. untreated control.

Supplementary Figure 6

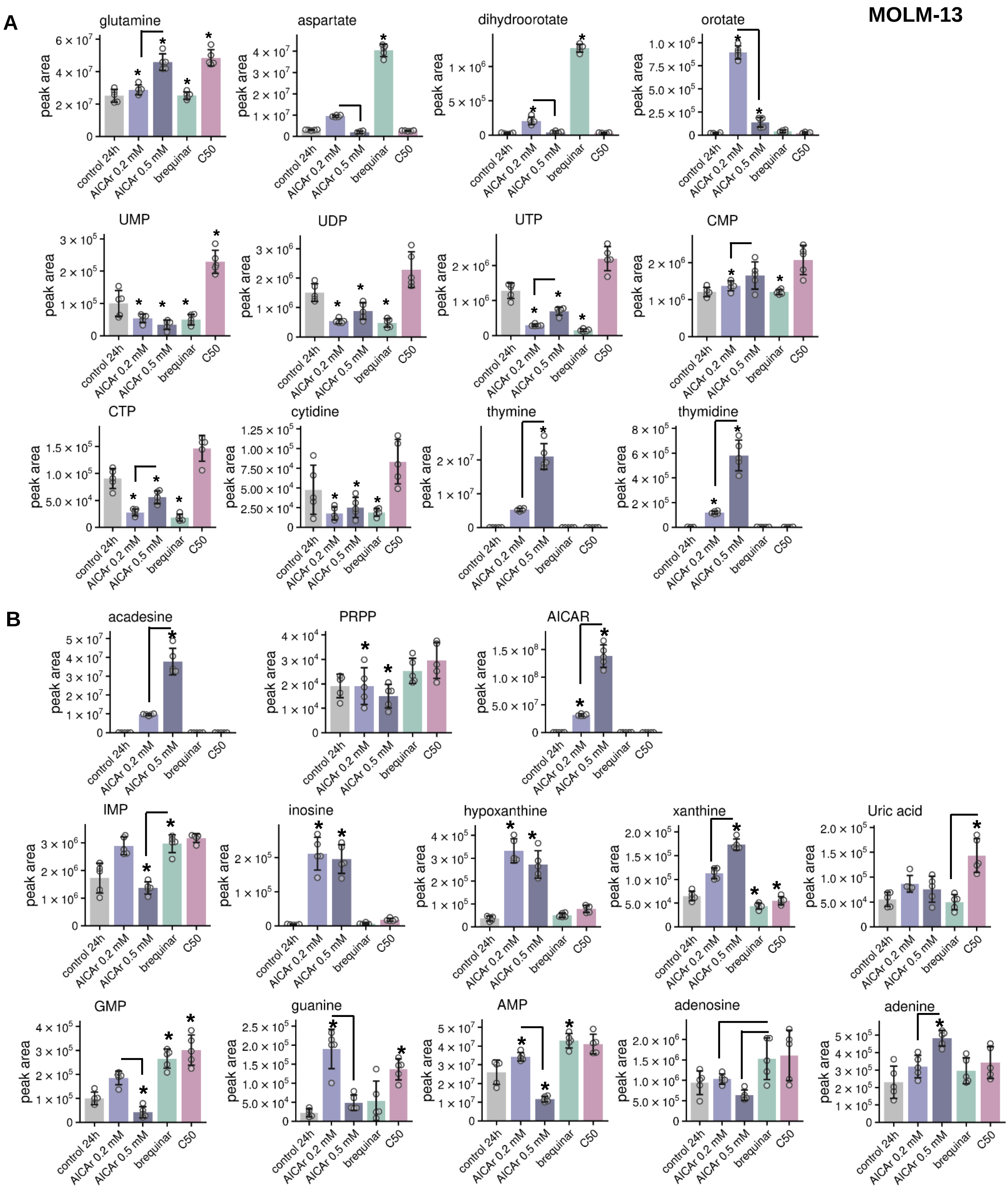

Supplementary Figure 6. Pyrimidine and Purine Metabolism in MOLM-13 cells upon AICAr, brequinar, and AraC treatment.

MOLM-13 cells were treated with AICAr (0.2 or 0.5 mM), brequinar (0.5 µM), or 50 nM AraC (C50) for 24 h. Intracellular levels of pyrimidine (A) and purine (B) pathway metabolites were measured by LC/MS. Data represent two independent experiments performed in quintuplicate and are shown as mean ± SD. *P < 0.05 vs. control; – indicates P < 0.05 between groups.

Supplementary Figure 7 **MOLM-13**

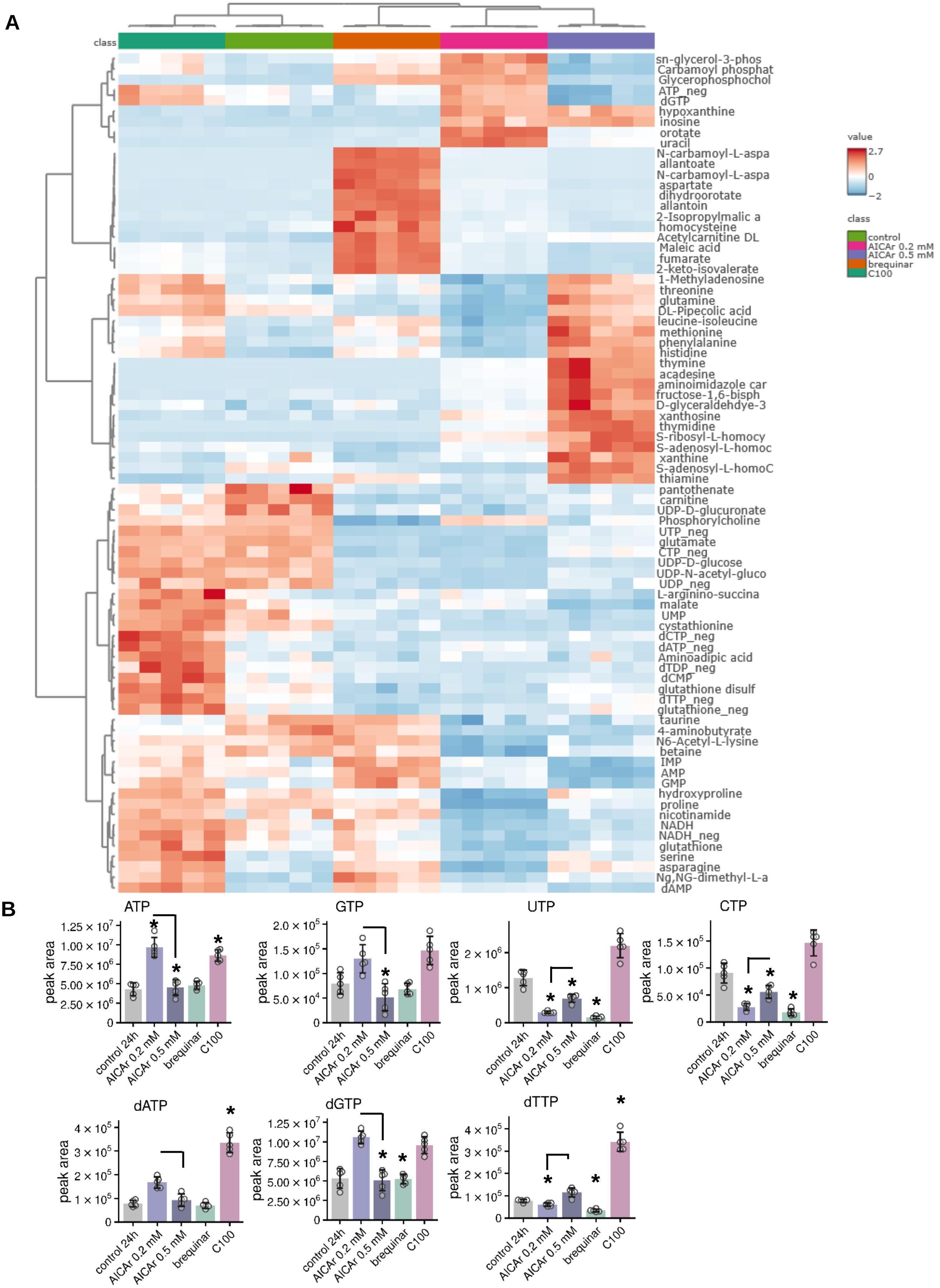

Supplementary Figure 7. Heatmap clustering and nucleotide pools in MOLM-13 cells upon AICAr, brequinar, and AraC treatment. (A) Heatmap showing hierarchical clustering of metabolite profiles across five treatment groups: control, AICAr (0.2 mM), AICAr (0.5 mM), brequinar (0.5 µM), and 50 nM AraC (C100).

(B) Intracellular levels of purine and pyrimidine ribonucleotides and deoxyribonucleotides were measured by LC/MS after 24 h treatment.

Supplementary Figure 8

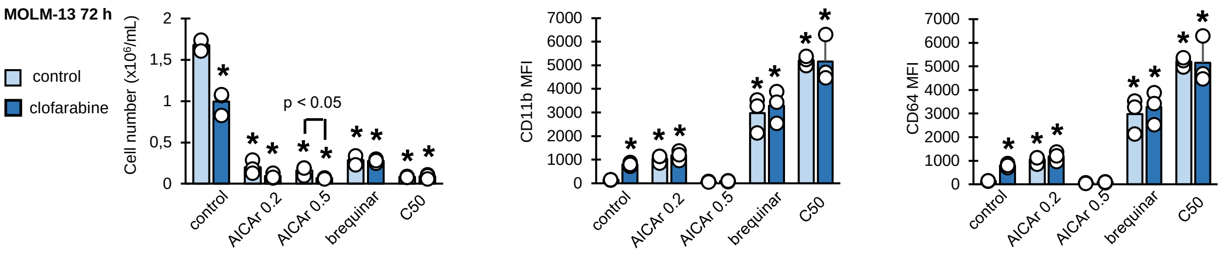

Supplementary Figure 8. Effects of clofarabine on viability and differentiation marker expression in MOLM-13 cells.

MOLM-13 cells were treated with AICAr (0.2 or 0.5 mM), brequinar (0.5 µM), or 50 nM AraC (C50) in the presence (+) or absence (−) of 30 nM clofarabine. Cell viability and CD11b/CD64 expression were assessed 72 h after treatment. Data represent mean ± SD of at least three independent experiments. **P* < 0.05 vs. untreated control.

Supplementary Figure 9

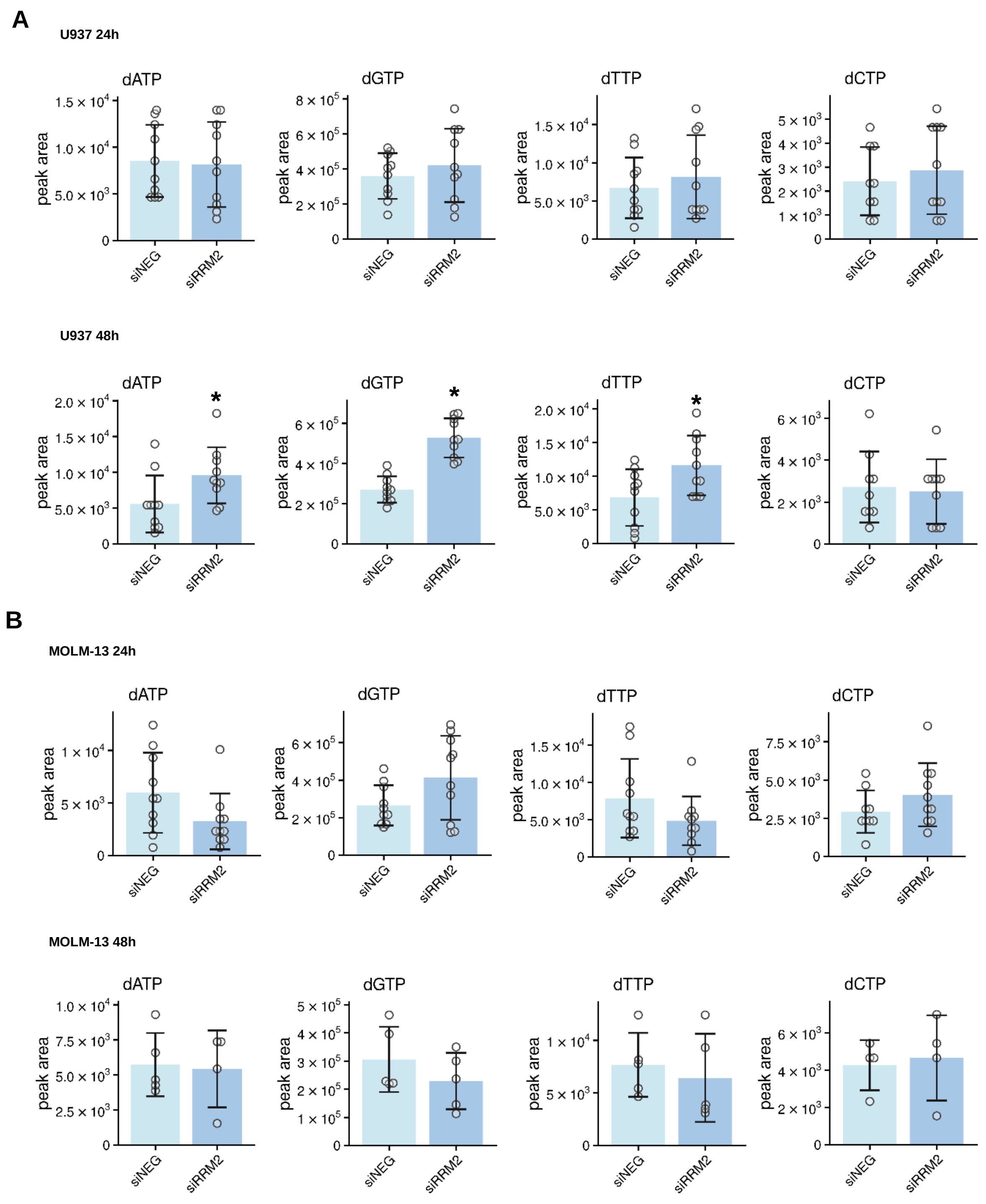

Supplementary Figure 9. RRM2 knockdown differentially affects intracellular dNTP pools in U937 and MOLM-13 cells. Intracellular deoxyribonucleotide triphosphate (dATP, dCTP, dGTP, and dTTP) levels were quantified by LC–MS at 24 and 48 h after transfection of (A) U937 and (B) MOLM-13 cells with siRNA targeting RRM2 (siRRM2) or non-targeting control siRNA (siNEG). Metabolite levels are shown as peak areas normalized to viable cell number. Each point represents an individual sample; horizontal lines indicate mean values. Statistical significance was assessed on log10-transformed data using an unpaired two-tailed Welch's t-test. *$P < 0.05$.

Supplementary Figure 10

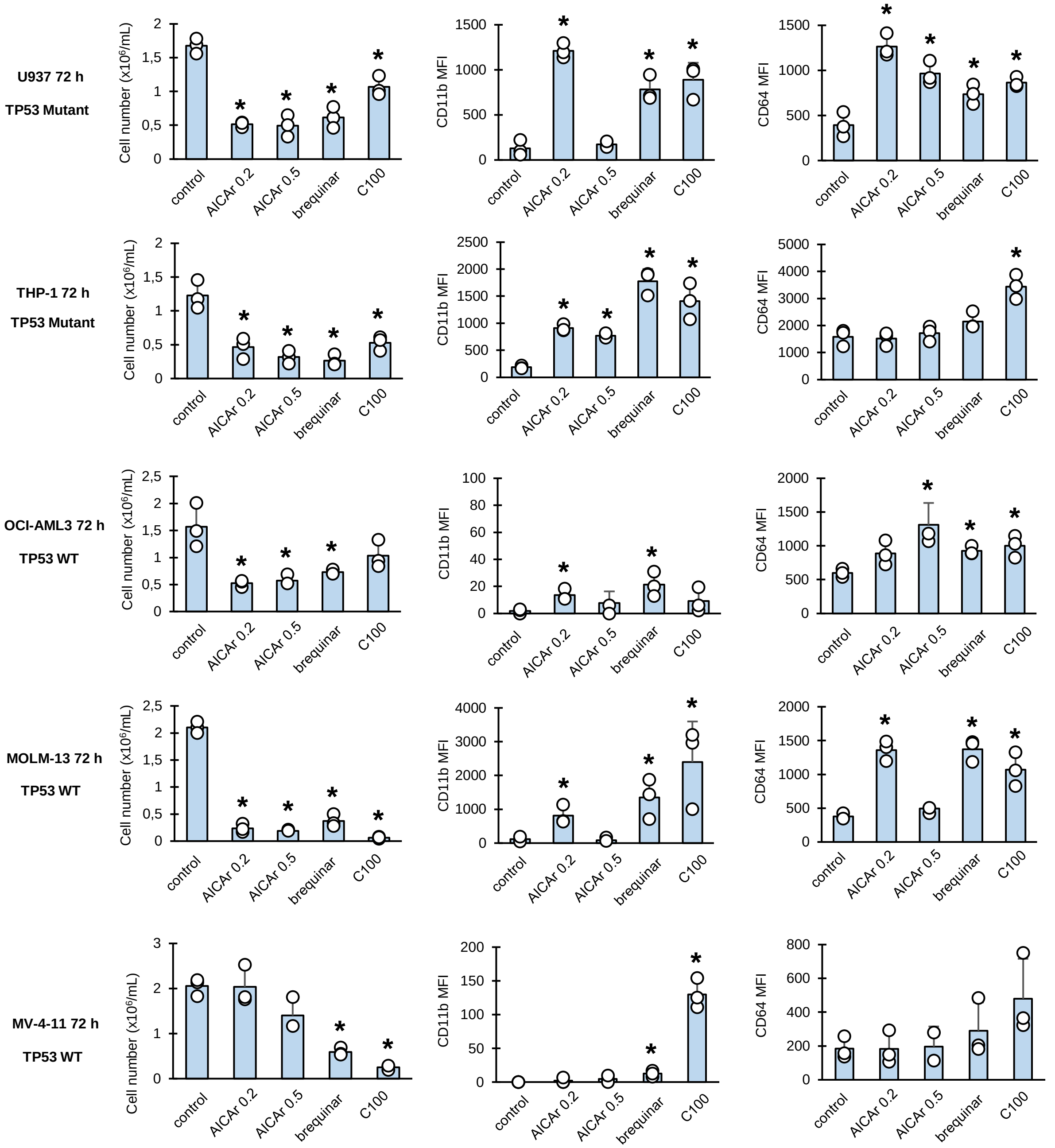

Supplementary Figure 10. Replication stress–inducing agents promote differentiation marker expression across monocytic AML cell lines with differing TP53 status

AML cell lines with different TP53 status (wild-type (WT) or mutant) were treated with AICAr (0.2 or 0.5 mM), brequinar (0.5 µM), or 100 nM AraC (C100). Cell viability and CD11b/CD64 expression were assessed 72 h after treatment. Data represent mean ± SD of three independent experiments. *$P < 0.05$ vs. untreated control.

Supplementary Figure 11

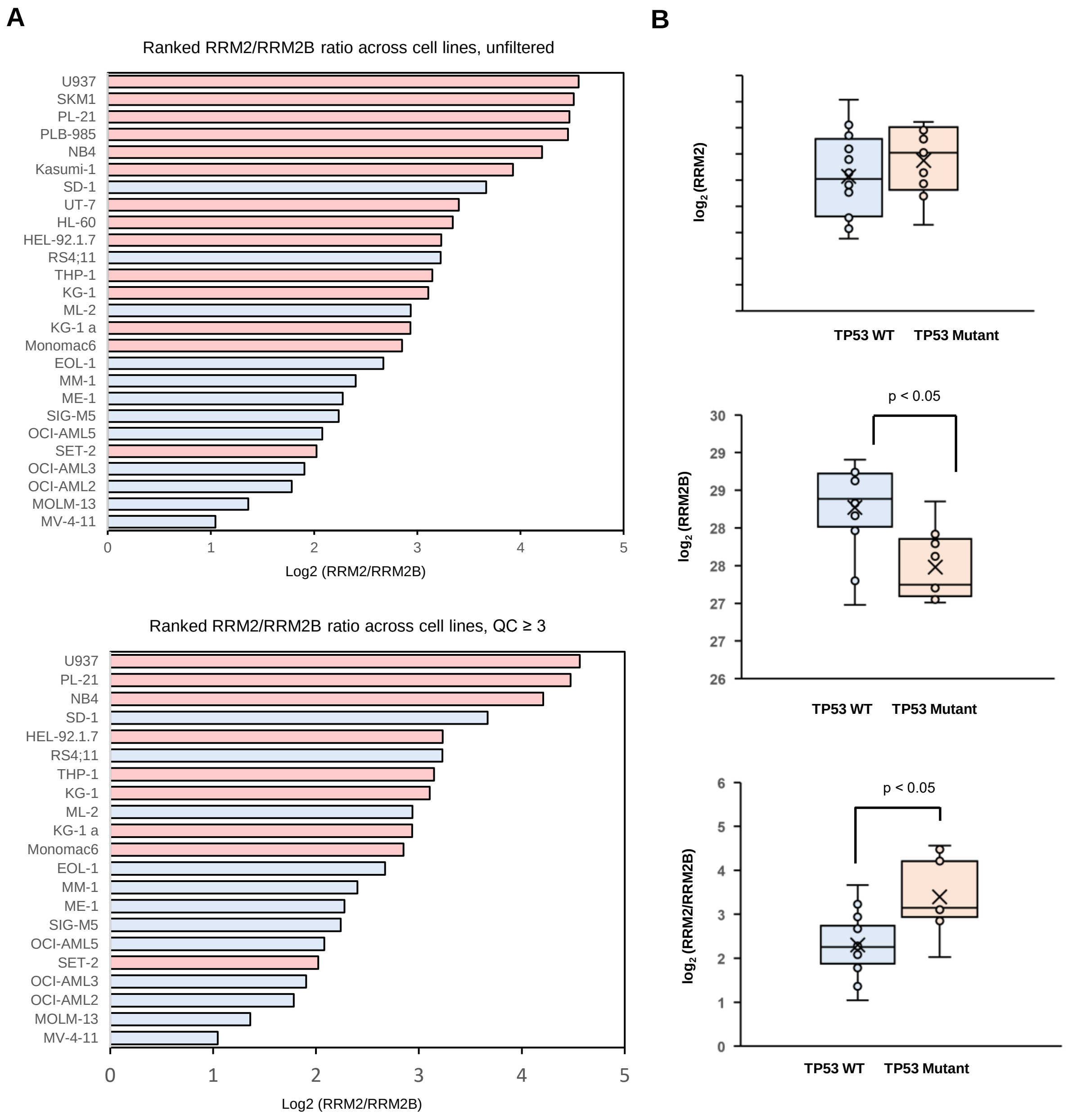

Supplementary Figure 11. TP53-associated differences in RRM2/RRM2B balance across AML cell lines.

Public proteomic data (27) were used to calculate median $\log_2$(RRM2/RRM2B) ratios across leukemia cell lines.

(A) Ranked ratios for all cell lines (unfiltered) and for a quality-controlled subset with ≥3 technical replicates (QC ≥ 3), color-coded by TP53 status (wild-type (WT), blue; mutant, pink).

(B) Box plots comparing $\log_2$(RRM2/RRM2B) ratios between TP53-wildtype and TP53-mutant AML cell lines; boxes indicate interquartile range with median, whiskers indicate range, and points represent individual cell lines. Statistical significance was assessed using a two-sided Mann–Whitney U test.

Supplementary Table 1. Molecular correlates of drug response and replication stress in primary AML samples

Supplementary Table 1A

Association of TP53 status with CHEK1 (Thr279) phosphorylation, RNR proteins, DHODH inhibitor DSS and cytarabine DSS

Tested variable	Median (WT)	Median (mut)	p-value	N (WT/MUT)
**RRM1**	77.22	76.69	0.716	29/11
**RRM2**	3.75	4.04	0.492	29/11
**RRM2B**	2.23	3.66	0.419	29/11
**RRM2/RRM2B**	1.89	1.66	0.963	29/11
**CHEK1 (T279)**	2.06	4.42	0.112	29/11
**DHODH inh. DSS**	0	0	0.623	23/7
**Cytarabine DSS**	8.7	5.0	0.049	23/9

Supplementary Table 1B

Association of CHEK1 (Thr279) phosphorylation with RNR proteins

variable	Tested variable	association	p-value	n
**CHEK1 (T279)**	**RRM1**	ρ = 0.25	0.067	55
**CHEK1 (T279)**	**RRM2**	**0.49**	**1.4×10^{-4}**	**55**
**CHEK1 (T279)**	**RRM2B**	0.16	0.25	39
**CHEK1 (T279)**	**RRM2/RRM2B**	**0.424**	**0.00127**	**55**

Supplementary Table 1C

Association between RRM abundance and differentiation markers in primary AML samples

variable	marker	Spearman	p-value	n
**RRM2/RRM2B**	**MPO**	0.149	0.307	55
**RRM2/RRM2B**	**ITGAM**	0.189	0.168	55
**RRM2/RRM2B**	**FCGR1**	**0.278**	**0.04**	**55**
**RRM2**	**MPO**	0.074	0.593	55
**RRM2**	**ITGAM**	0.194	0.155	55
**RRM2**	**FCGR1**	**0.346**	**0.01**	**55**
**RRM2B**	**MPO**	0.024	0.864	55
**RRM2B**	**ITGAM**	0.127	0.356	55
**RRM2B**	**FCGR1**	0.125	0.362	55

Supplementary Table 1D

Correlation of DHODH inhibitor (vidofludimus) DSS with molecular features

variable	Tested variable	association	p-value	n
**DSS**	**RRM1**	0.191	0.243	39
**DSS**	**RRM2**	-0.081	0.622	39
**DSS**	**RRM2B**	0.265	0.103	39
**DSS**	**RRM2/RRM2B**	-0.268	0.099	39

**DSS**	**CHEK1 (T279)**	0.029	0.861	39
**DSS**	**MPO**	0.026	0.876	39
**DSS**	**ITGAM**	**-0.364**	**0.023**	**39**
**DSS**	**FCGR1**	-0.280	0.084	39
**DSS**	**TP53wt**	r_pb −0.132	0.488	WT = 23, MUT = 7

Supplementary Table 1E

Correlation of cytarabine DSS with molecular features

variable	Tested variable	association	p-value	n
**DSS**	**RRM1**	0.066	0.661	47
**DSS**	**RRM2**	0.007	0.965	47
**DSS**	**RRM2B**	-0.034	0.818	47
**DSS**	**RRM2/RRM2B**	0.003	0.983	47
**DSS**	**CHEK1 (T279)**	0.082	0.584	47
**DSS**	**MPO**	0.192	0.196	47
**DSS**	**ITGAM**	-0.207	0.163	47
**DSS**	**FCGR1**	0.088	0.556	47
**DSS**	**TP53wt**	r_pb – 0.299	0.096	WT = 23, MUT = 9

Supplementary Table 1. Molecular correlates of replication stress signaling and drug response in primary AML samples.

(A) TP53 status was determined by DNA sequencing and samples were grouped as TP53 wild-type versus TP53 mutant. Values represent median log_2-transformed protein intensities. Group differences in continuous molecular features were assessed using the Mann–Whitney U test.

(B-E) Associations with continuous variables were assessed using Spearman correlation. TP53 status was encoded as a binary variable (0 = wild-type, 1 = mutant), and paired associations with continuous variables were assessed using point-biserial correlation.

Supplementary Table 2. Reagents and resources used

REAGENT or RESOURCE	SOURCE	IDENTIFIER
**Antibodies**		
IgG1-FITC (clone 679.1Mc7)	Immunotech BeckmanCoulter, Marseille France	A07795 RRID:AB_2832964
CD11b-FITC (clone Bear1)	Immunotech BeckmanCoulter, Marseille France	IM0530 RRID:AB_130987
CD64-FITC (clone 22)	Immunotech BeckmanCoulter, Marseille France	B49185
Anti-mouse IgG, HRP-linked antibody	Cell Signaling Technology, Beverly, MA, USA	7076 RRID:AB_330924
Anti-rabbit IgG, HRP-linked antibody	Cell Signaling Technology, Beverly, MA, USA	7074 RRID:AB_2099233
p53R2	Abcam, Cambridge, UK	AB8105 RRID:AB_306275
GAPDH (H-12)	Santa Cruz Biotechnology, Dallas, Texas, USA	I1013
GAPDH (D16H11)	Cell Signaling Technology, Beverly, MA, USA	5174 RRID:AB_10622025
alpha-Tubulin (11H10)	Cell Signaling Technology, Beverly, MA, USA	2125 RRID:AB_2619646
RRM2 (E7Y9J)	Cell Signaling Technology, Beverly, MA, USA	65939 RRID:AB_2895029
P-RRM2 (T33)	Cell Signaling Technology, Beverly, MA, USA	74736
Histone H3 (D1H2) XP®	Cell Signaling Technology, Beverly, MA, USA	4499 RRID:AB_10544537
**siRNA transfection**		
ON-TARGETplus SMARTpool Human RRM2 siRNA	Dharmacon, Lafayette, CO, USA	L-010379-00-0005
ON-TARGETplus™ Control Pool, Non-Targeting Pool	Dharmacon, Lafayette, CO, USA	D-001810-10-05
Nuclease-Free Water	Invitrogen, Thermo Fisher Scientific, Waltham, MA, USA	9914G
Neon Transfection System 100 µl kit	Invitrogen, Thermo Fisher Scientific, Waltham, MA, USA	MPK10096
**Chemicals and peptides**		
Cytosine β-D-arabinofuranoside	Sigma, St. Louis, MO, USA	C1786
AICAr	Sigma, St. Louis, MO, USA	A9978
Brequinar	Sigma, St. Louis, MO, USA	SML0113
COH29	MedChem Express, Monmouth Junction, New Jersey, USA	1190932-38-7
hydroxyurea	Sigma, St. Louis, MO, USA	400046
clofarabine	Sigma-Aldrich, St. Louis, MO, USA	C7495
7-AAD staining solution	Miltenyi Biotec GmbH, Bergisch Gladbach, Germany	130-111-568

bovine serum albumin (BSA)	Sigma, St. Louis, MO, USA	A4503
tween® 20	Sigma, St. Louis, MO, USA	P7949
Trypan blue stain (0.4%)	Gibco, Life Technologies, Grand Island, NY, USA	15250-061
Methanol ROTIPURAN ® 99%	Carl Roth GmbH & Co. Kg, Karlsruhe, Germany	4627.1
MOPS SDS Running Buffer (20X)	Invitrogen, Thermo Fisher Scientific, Waltham, MA, USA	NP0001
NuPAGE™ Transfer Buffer (20X)	Invitrogen, Thermo Fisher Scientific, Waltham, MA, USA	NP00061
NuPAGE® LDS Sample Buffer (4X)	Invitrogen, Thermo Fisher Scientific, Waltham, MA, USA	NP0007
Skim Milk Powder	Sigma-Aldrich, St. Louis, MO, USA	70166
NuPAGE™ 4-12% Bis-Tris Gel, 1.0–1.5 mm, Mini Protein Gels	Invitrogen, Thermo Fisher Scientific, Waltham, MA, USA	NP0323BOX
2′-Deoxyguanosine monohydrate	Sigma-Aldrich, St. Louis, MO, USA	D7145
2′-Deoxyadenosine monohydrate	Sigma-Aldrich, St. Louis, MO, USA	D8668
2′-Deoxycytidine	Sigma-Aldrich, St. Louis, MO, USA	D3897
Thymidine	Sigma-Aldrich, St. Louis, MO, USA	T1895
Dimethyl sulfoxide (DMSO)	Honeywell, Muskegon, MI, USA	472301
Human TruStain FcX™ Fc Receptor Blocking solution	BioLegend, San Diego, CA, USA	422302
Bradford Dye Reagent	Thermo Fisher Scientific, Waltham, MA, USA	J61522
SuperSignal™ West Pico PLUS Chemiluminescent Substrate	Thermo Fisher Scientific, Waltham, MA, USA	34580
PVDF Transfer Membrane	Thermo Fisher Scientific, Waltham, MA, USA	88518
PageRuler™ Plus Prestained Protein Ladder, 10 to 250 kDa	Thermo Fisher Scientific, Waltham, MA, USA	26619
Sodium chloride, cryst.	Kemika, Zagreb, Croatia	1417506
propidium iodide	Sigma, St. Louis, MO, USA	P4170
Trizma® base	Sigma, St. Louis, MO, USA	T1503
RNAse	Sigma, St. Louis, MO, USA	R6513
Microcystin-LR	Enzo, Life Sciences, Farmingdale, NY, USA	ALX-350-012-C100
Phenylmethylsulfonyl fluoride (PMSF)	Sigma, St. Louis, MO, USA	P7626
igepal CA	Sigma, St. Louis, MO, USA	I3021
Sodium azide	Sigma, St. Louis, MO, USA	S8032
**Cell culture**		
RPMI Medium 1640	Life Technologies, Grand Island, NY, USA	42401-018

Fetal bovine serum (FBS)	Life Technologies, Grand Island, NY, USA	10270-106
L-Glutamine 200mM	Life Technologies, Grand Island, NY, USA	25030-024
Penicillin/streptomycin	Life Technologies, Grand Island, NY, USA	15070-063
Alpha MEM Eagle	Pan Biotech, Aidenbach, Germany	P04-21150
**Cell lines**		
U937	ECACC, Salisbury, UK	85011440 RRID:CVCL_0007
MOLM-13	kind gift from P. Gallipoli (obtained from Sanger Institute)	Cell Models Passport
THP-1	DSMZ (Leibniz Institute-Deutsche Sammlung von Mikroorganismen und Zellkulturen GmbH), Braunschweig, Germany	ACC 16
MV-411	kind gift from P. Gallipoli (obtained from Sanger Institute)	Cell Models Passport
OCI-AML3	kind gift from P. Gallipoli (obtained from Sanger Institute)	Cell Models Passport
**Softwares**		
FlowJo_v10.8.1	Tree Star Inc. Ashland, OR, USA	https://www.flowjo.com/solutions/flowjo RRID:SCR_008520
GraphPad Prism version 6.07	GraphPad Software, La Jolla, CA, USA	www.graphpad.com RRID:SCR_002798
Adobe Photoshop	Adobe Systems Inc., San Jose, CA, USA	Version CS6, 2012
MetaboAnalyst6.0	web browser	https://www.metaboanalyst.ca/ RRID:SCR_015539
Metabolite AutoPlotter v2.6, Pietzke, M., Vazquez, A. - Metabolite AutoPlotter	web browser	https://mpietzke.shinyapps.io/AutoPlotter/
