# Supplementary material for: Roles of RRM2 and RRM2B in pyrimidine stress responses and differentiation of acute myeloid leukemia cells
Source: Cell Death Discov. 2026 Apr 24;12:271. doi: 10.1038/s41420-026-03105-y (PMC13243657; doi:10.1038/s41420-026-03105-y)

Western blot original files

Fig.3 D WB1 RRM2

45 kDa

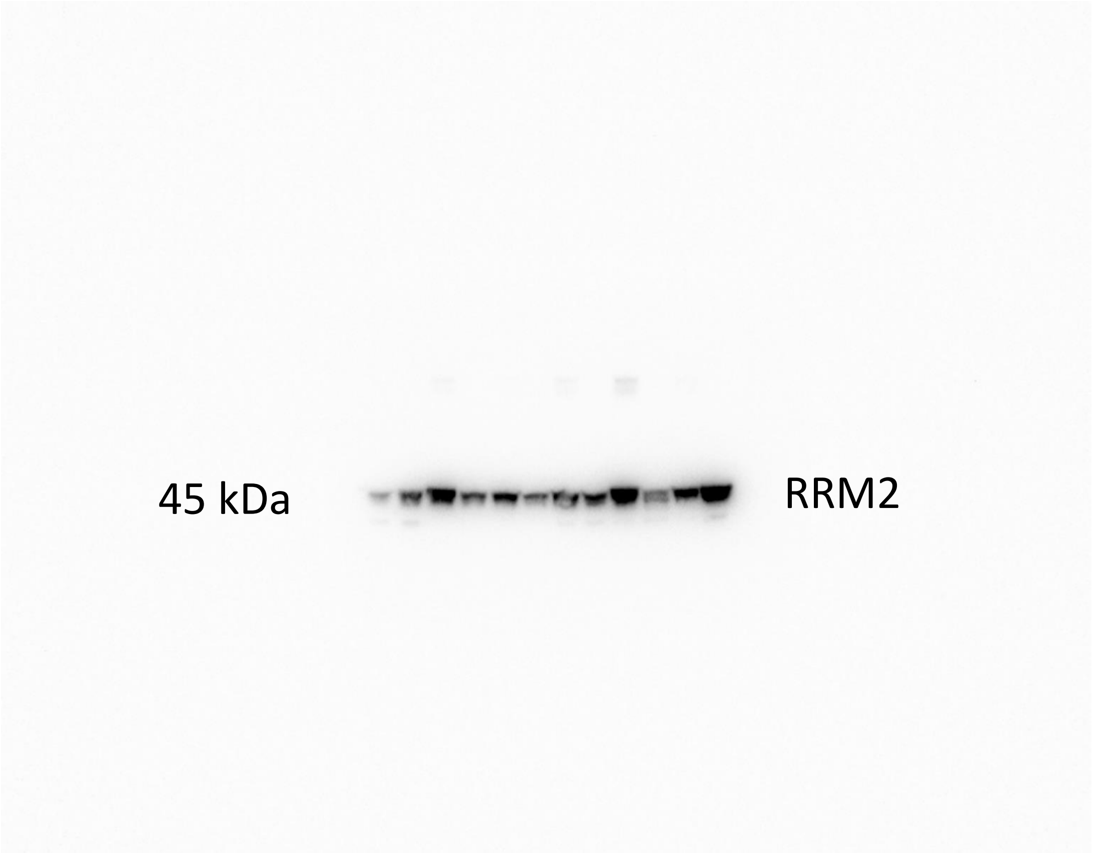

Fig.3 D WB1 β-actin

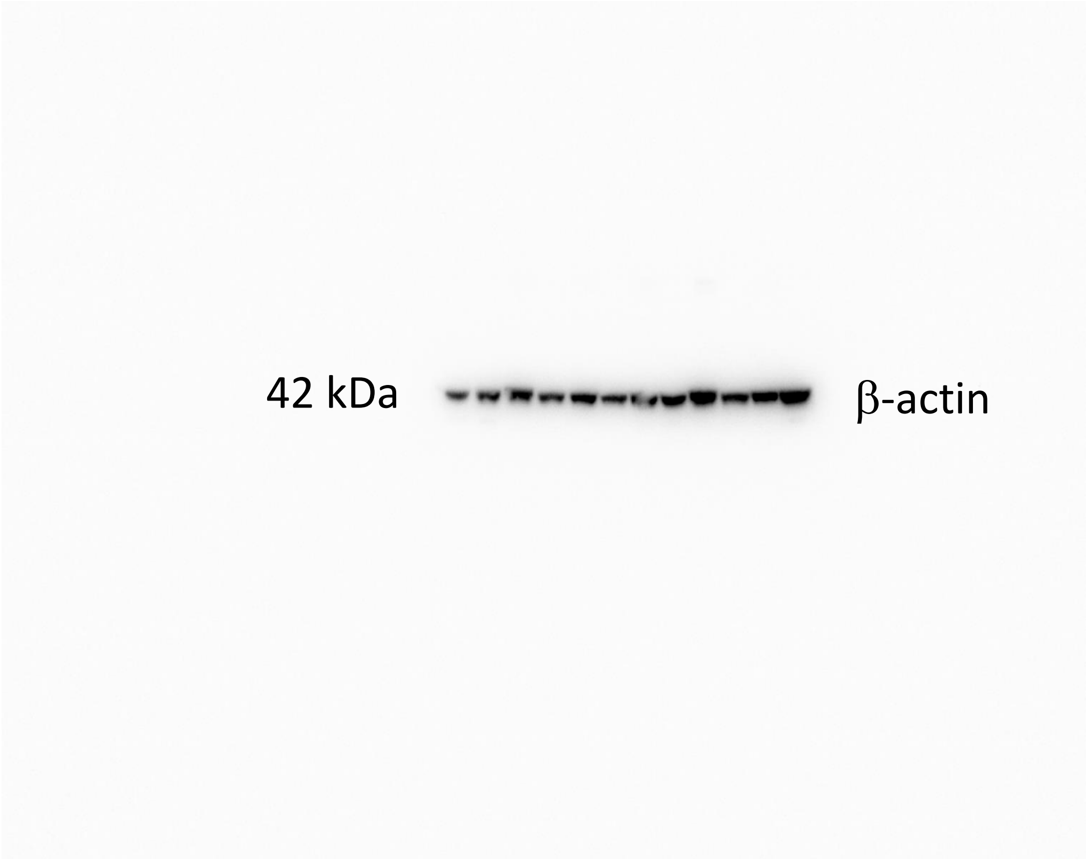

Fig.3 D WB2 p-RRM2

45 kDa 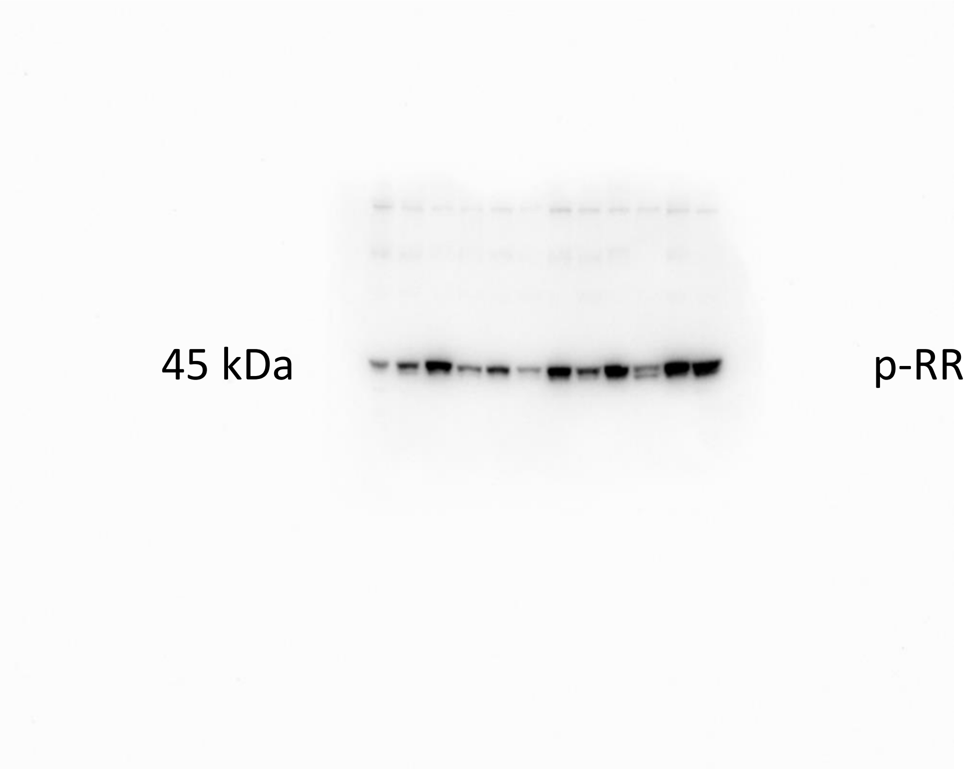 p-RRM2

Fig.3 D WB2 β-actin

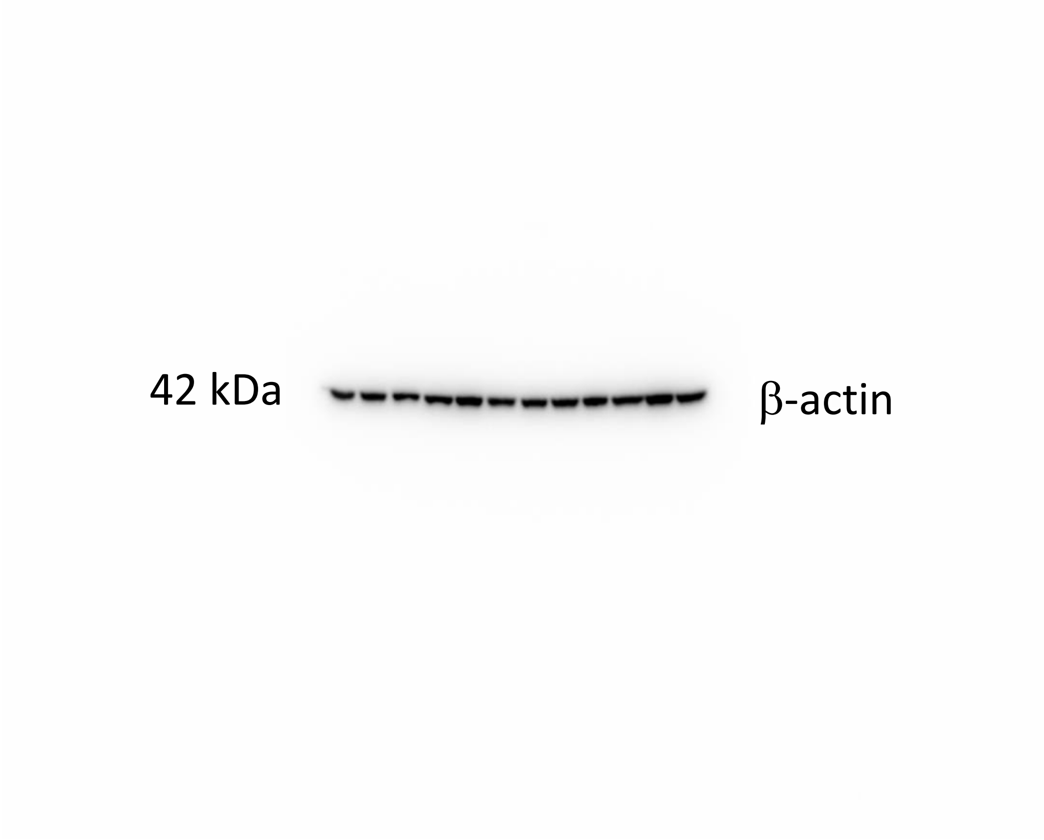

Fig.4 C WB1 RRM2

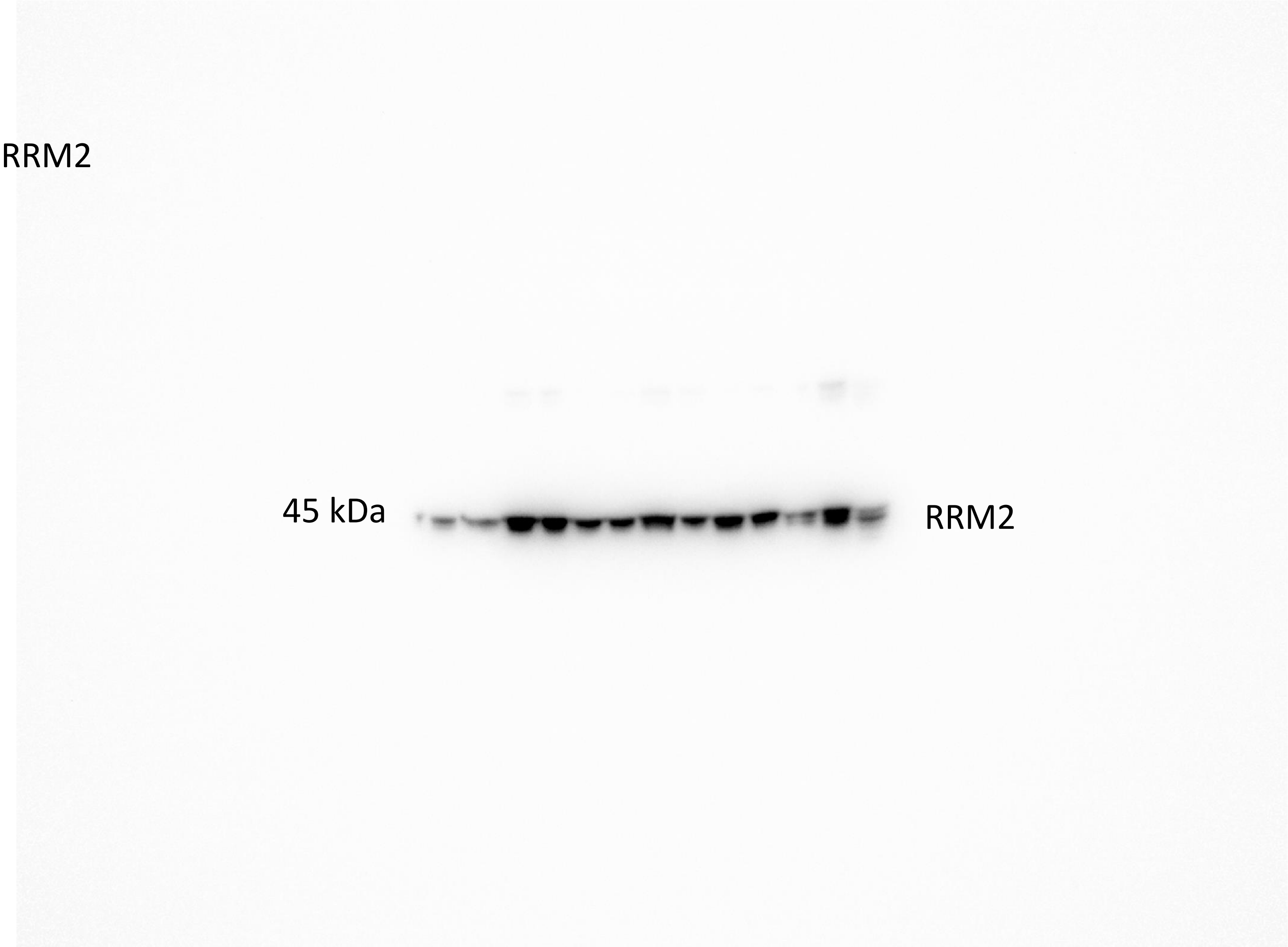

Fig.4 C WB1 GAPDH

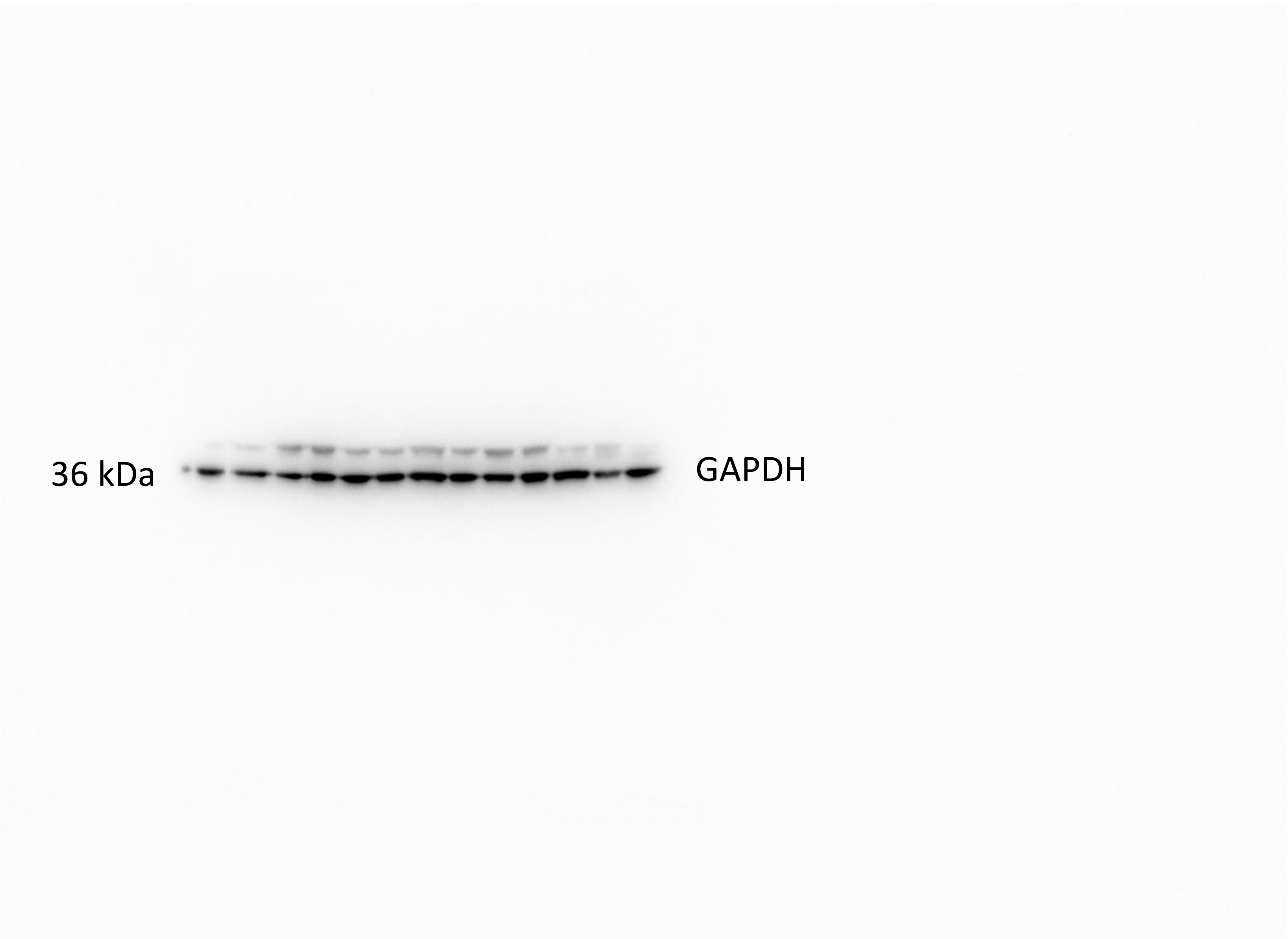

Fig.4 C WB2 p-RRM2

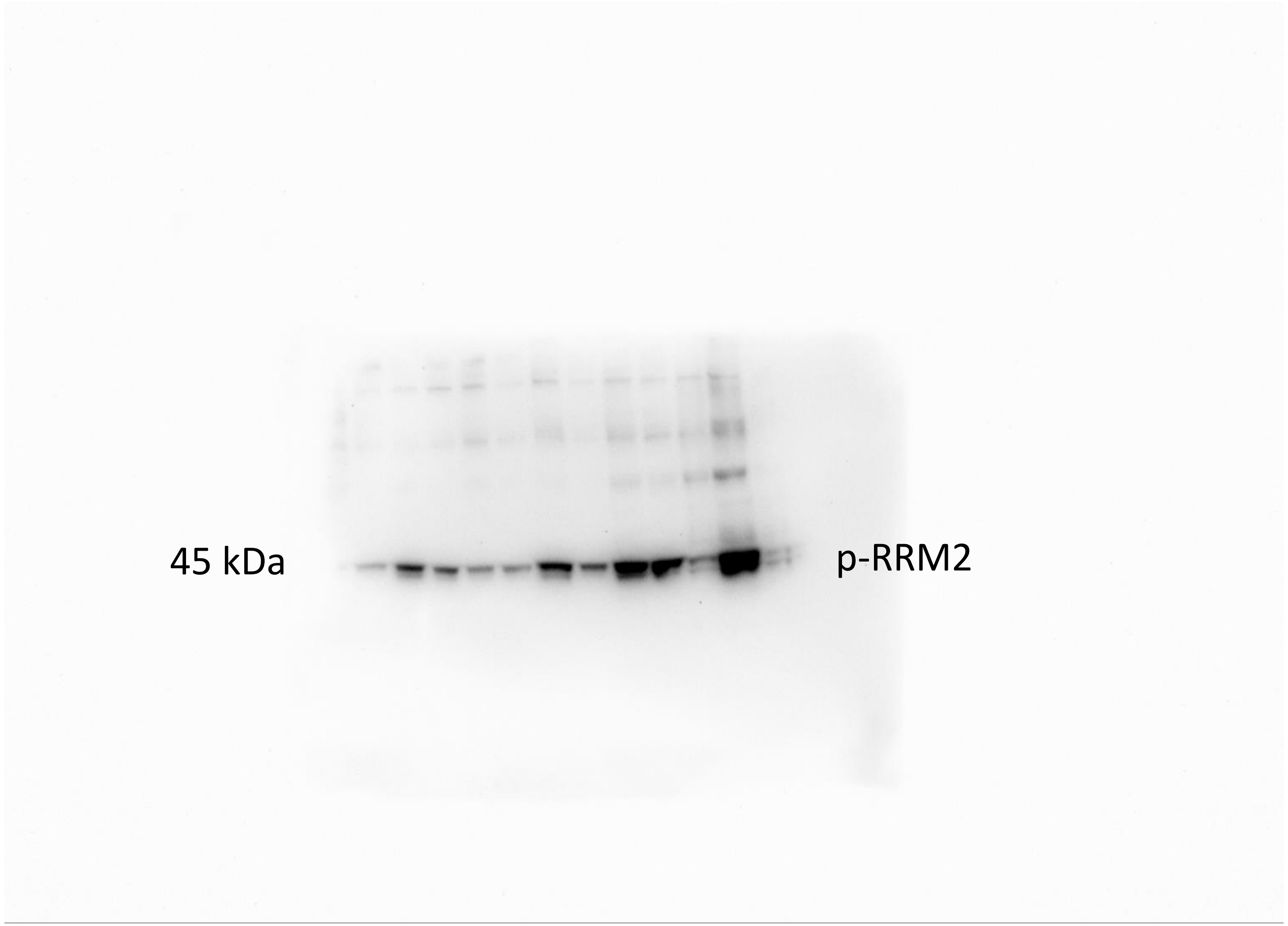

Fig.4 C WB2 GAPDH

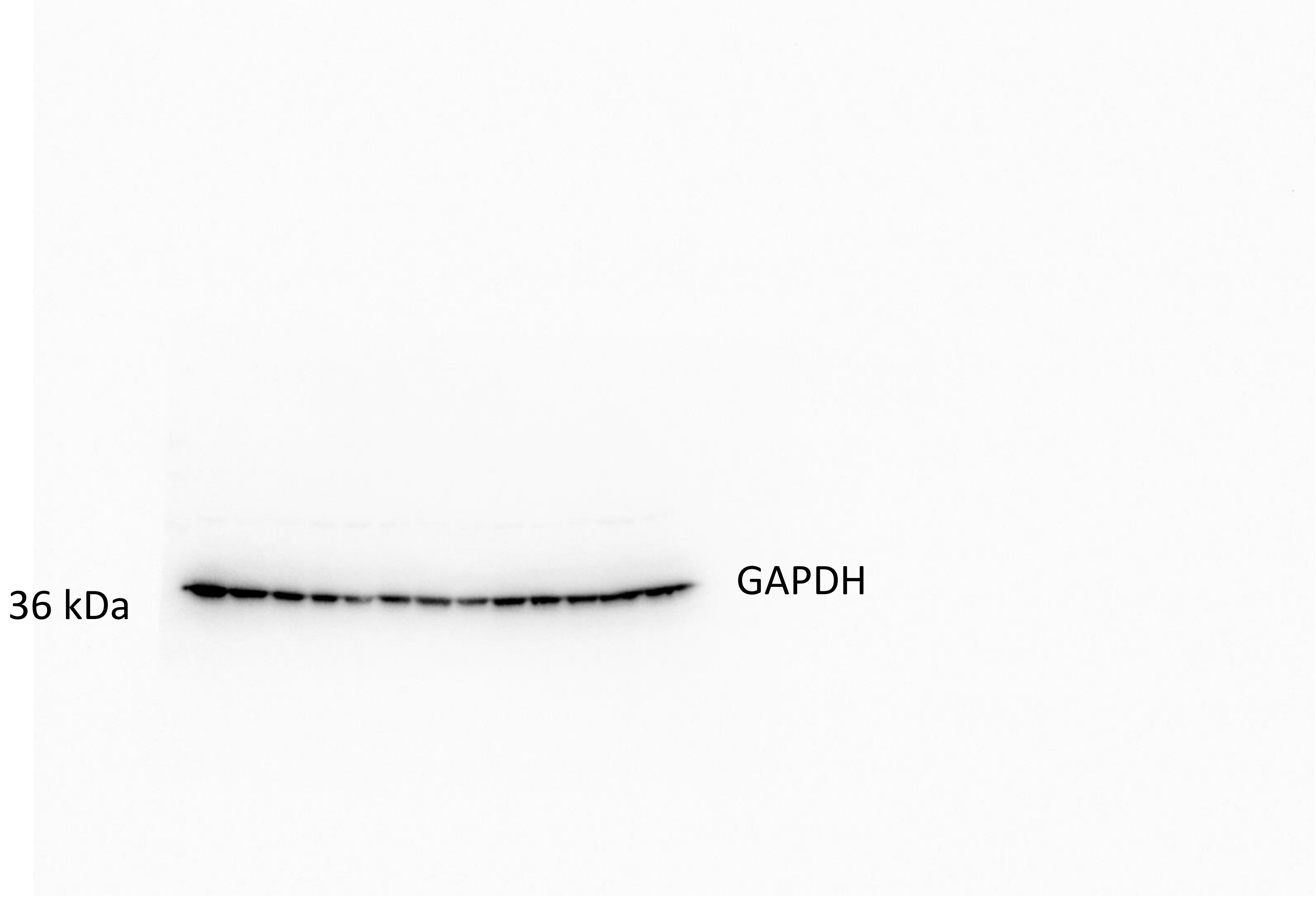

Fig.4 G WB1 RRM2

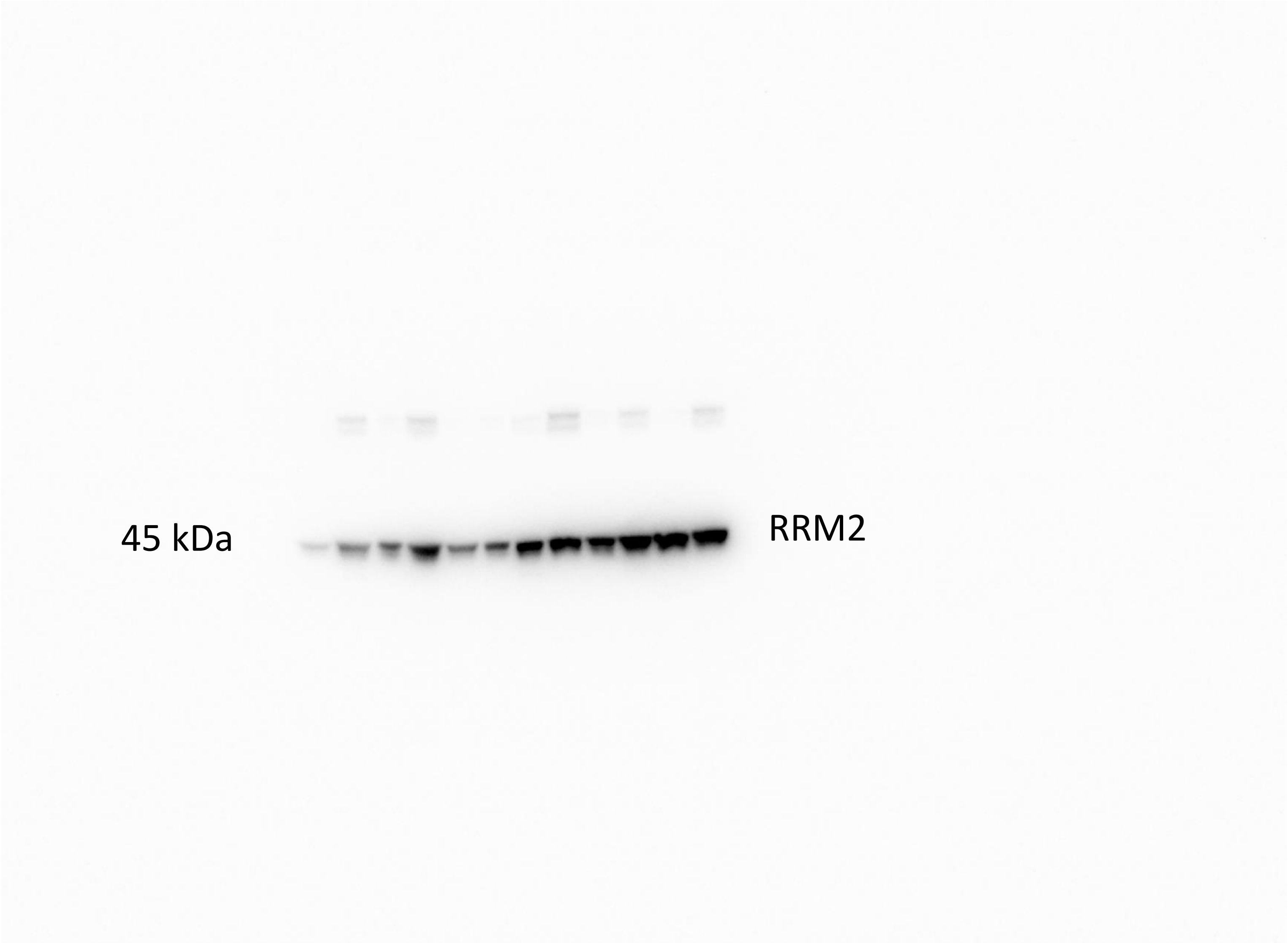

Fig.4 G WB1 GAPDH

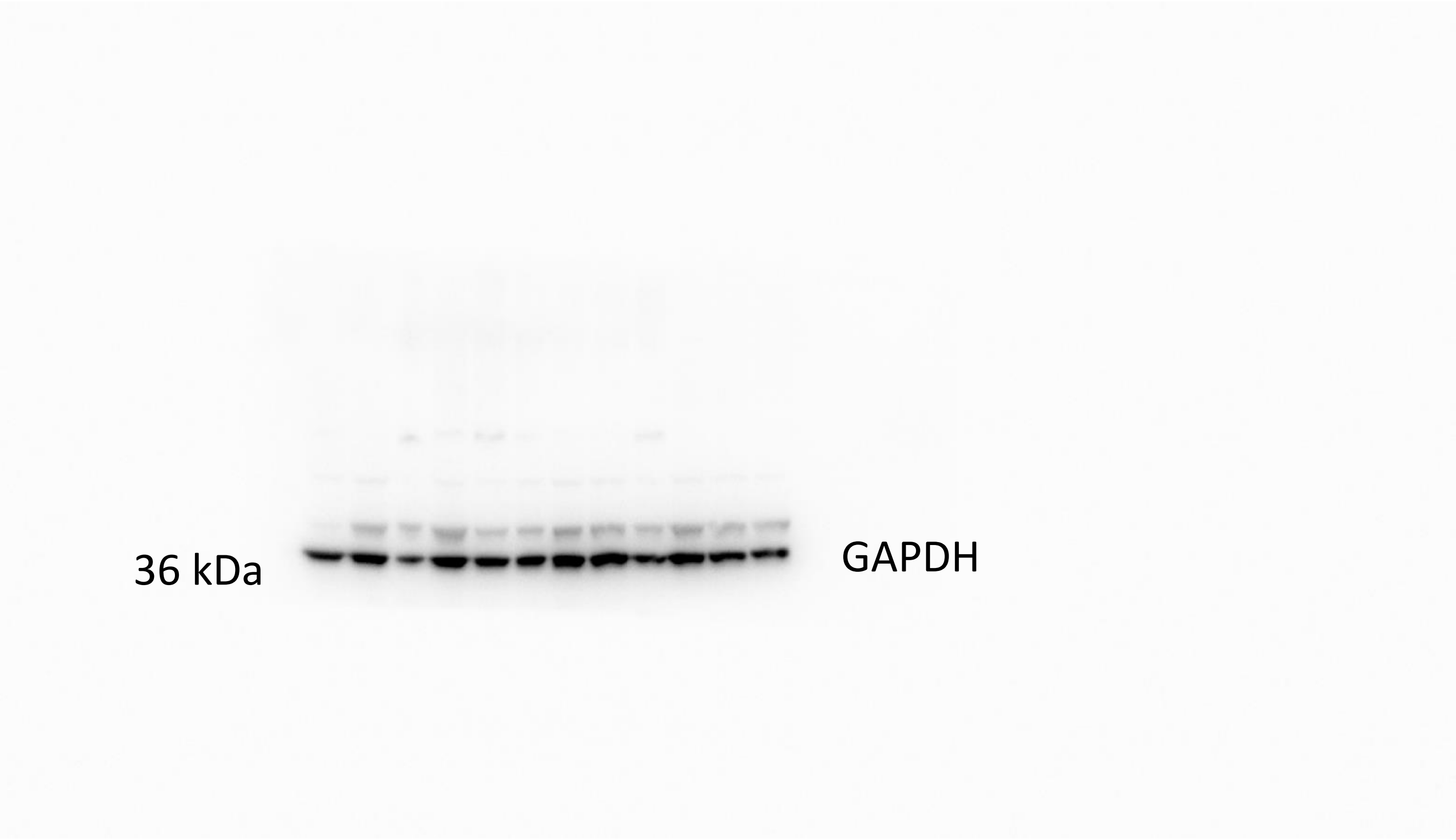

Fig.4 G WB2 RRM2

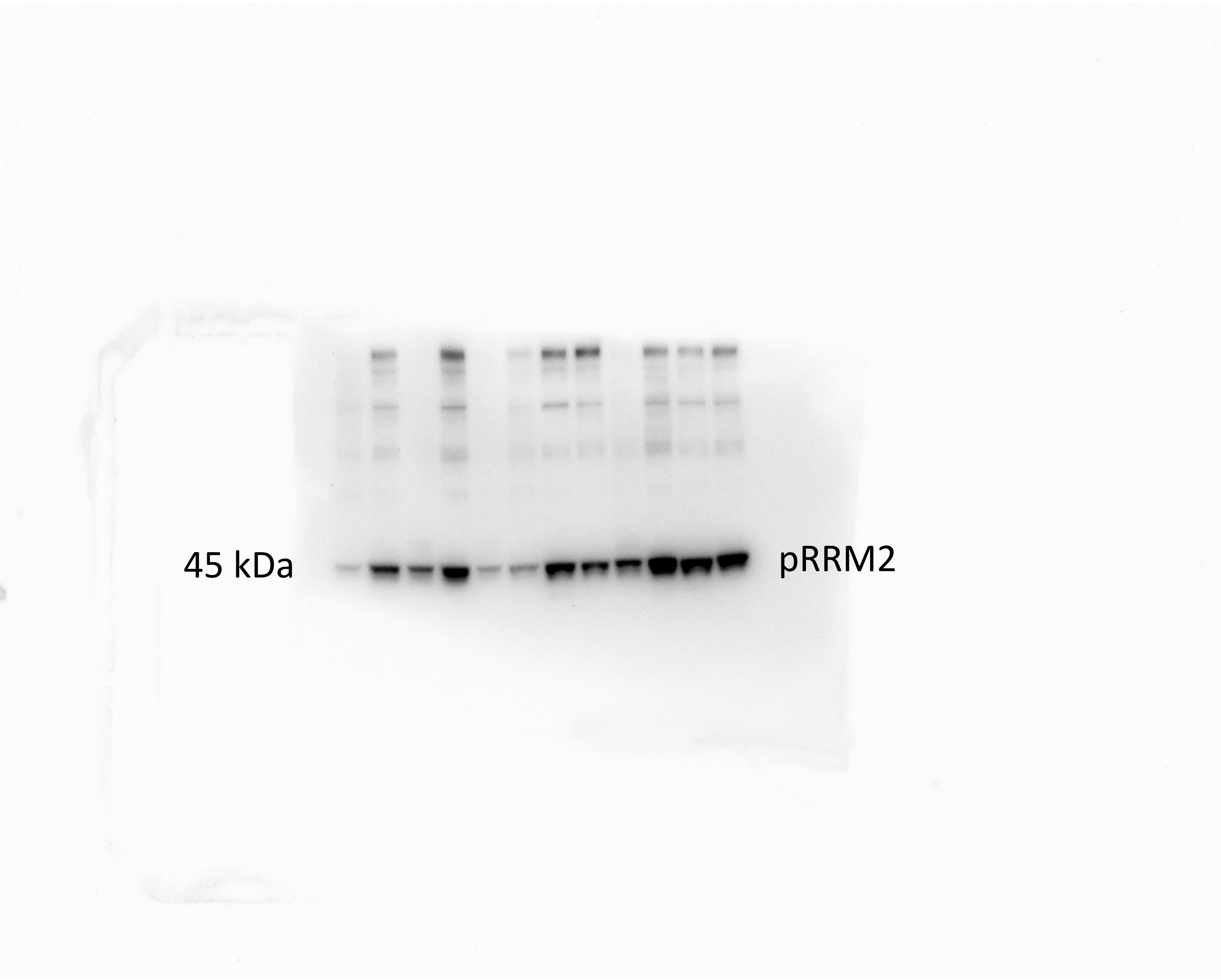

Fig.4 G WB2 GAPDH

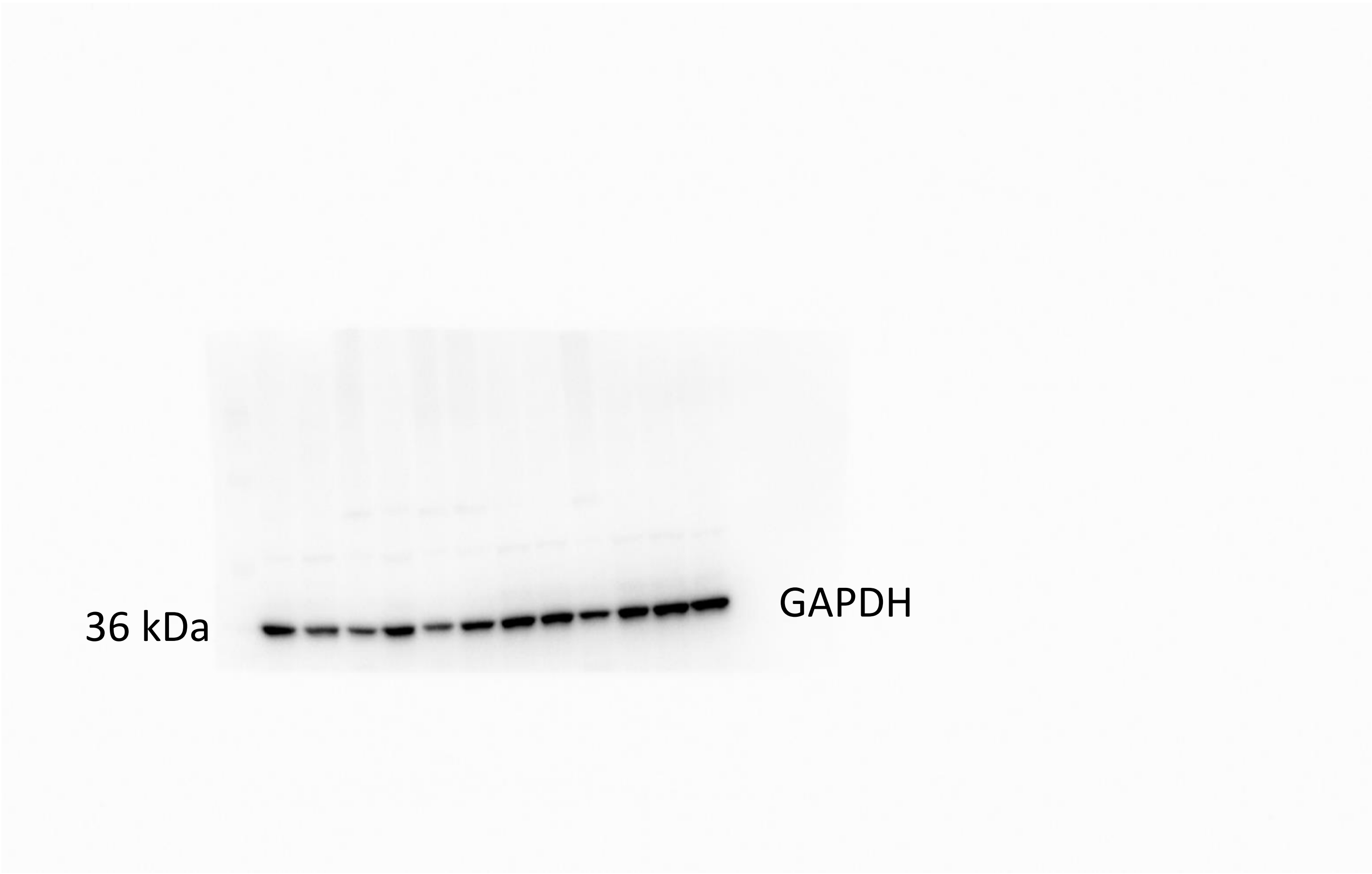

Fig.5 A WB1 EXP1 RRM2

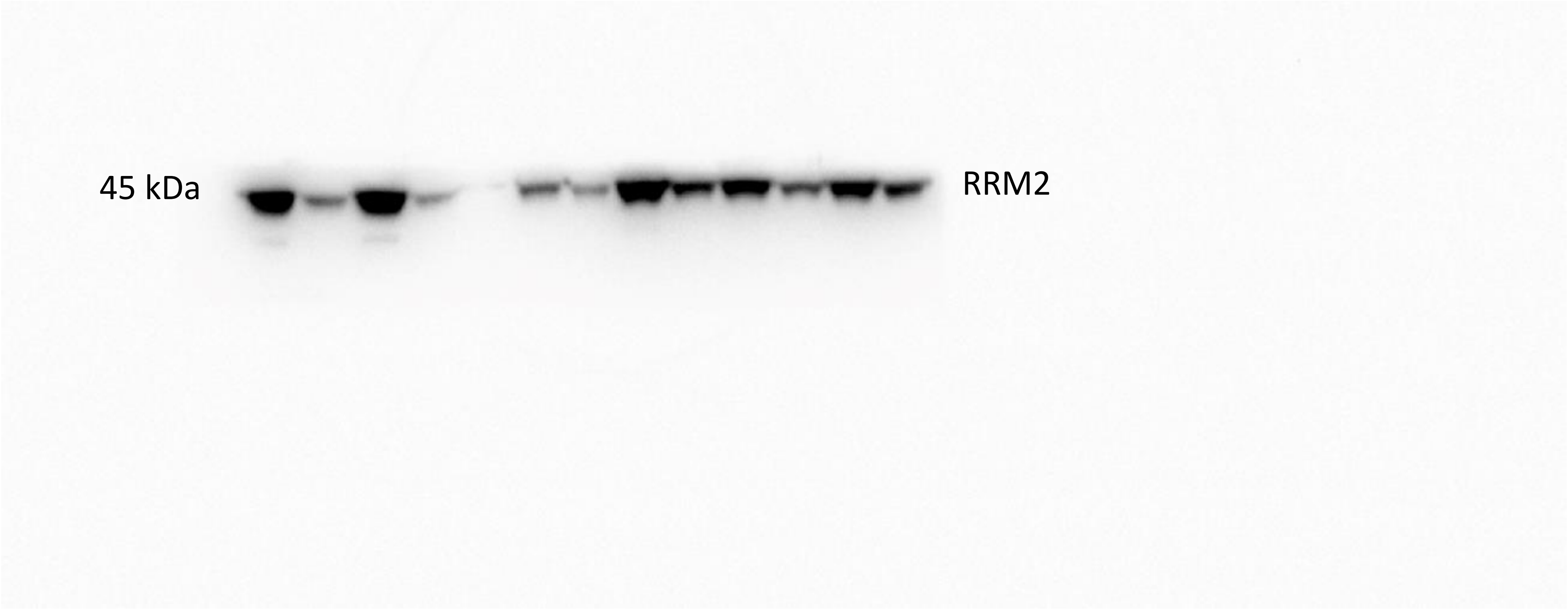

Fig.5 A WB1 EXP1 GAPDH

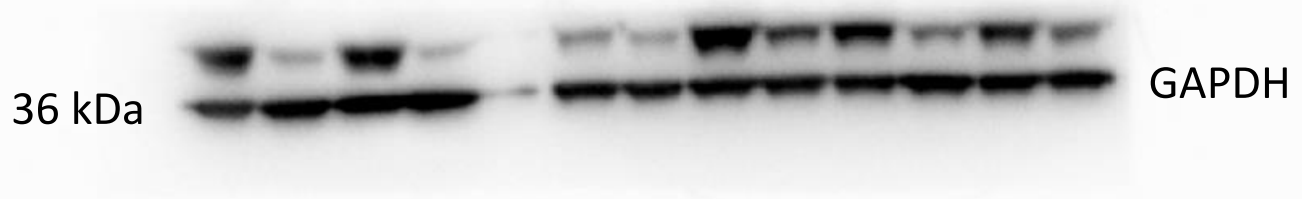

Fig.5 A WB1 EXP2 RRM2

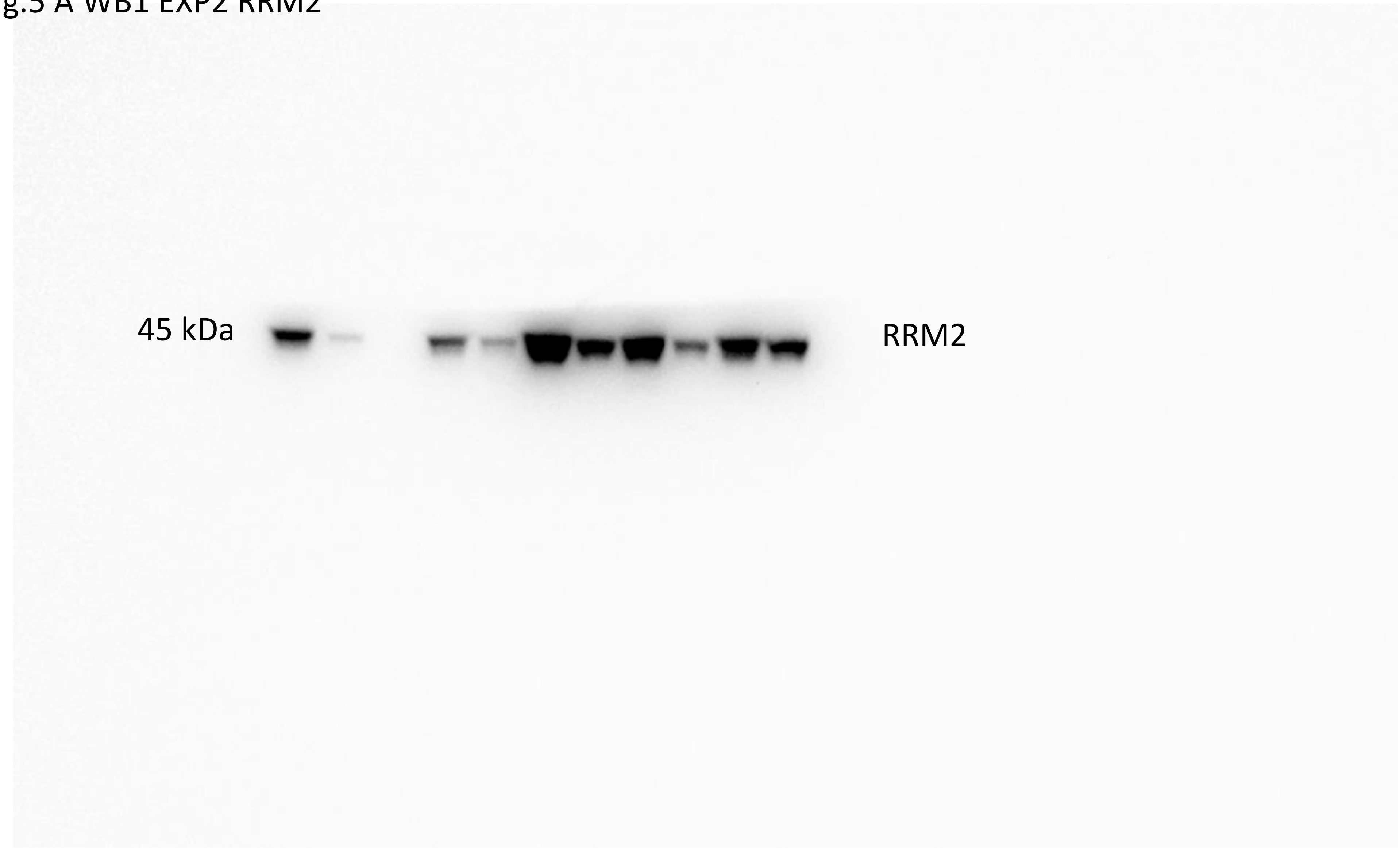

Fig.5 A WB1 EXP2 GAPDH

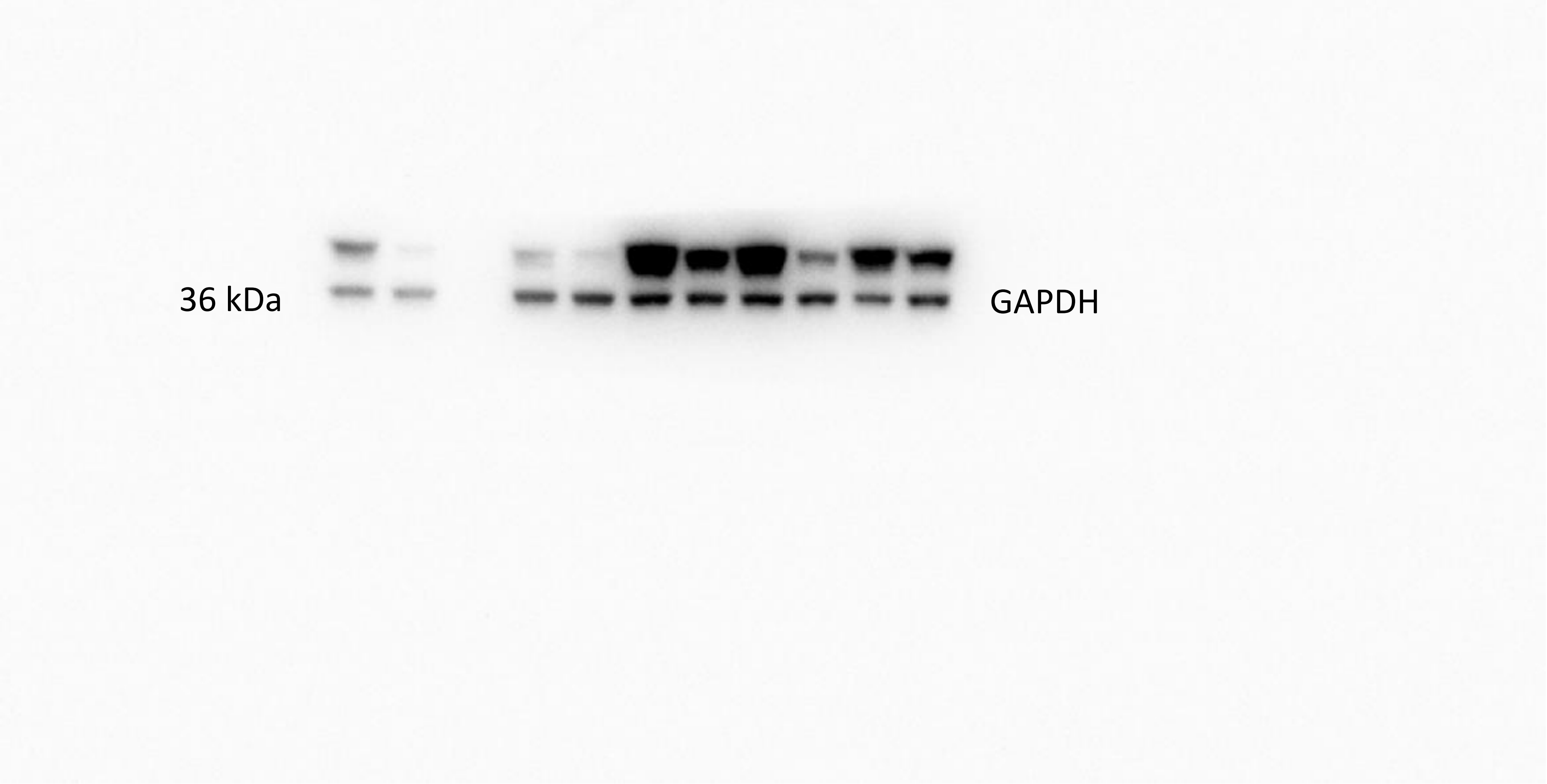

Fig.5 A WB1 EXP3 RRM2

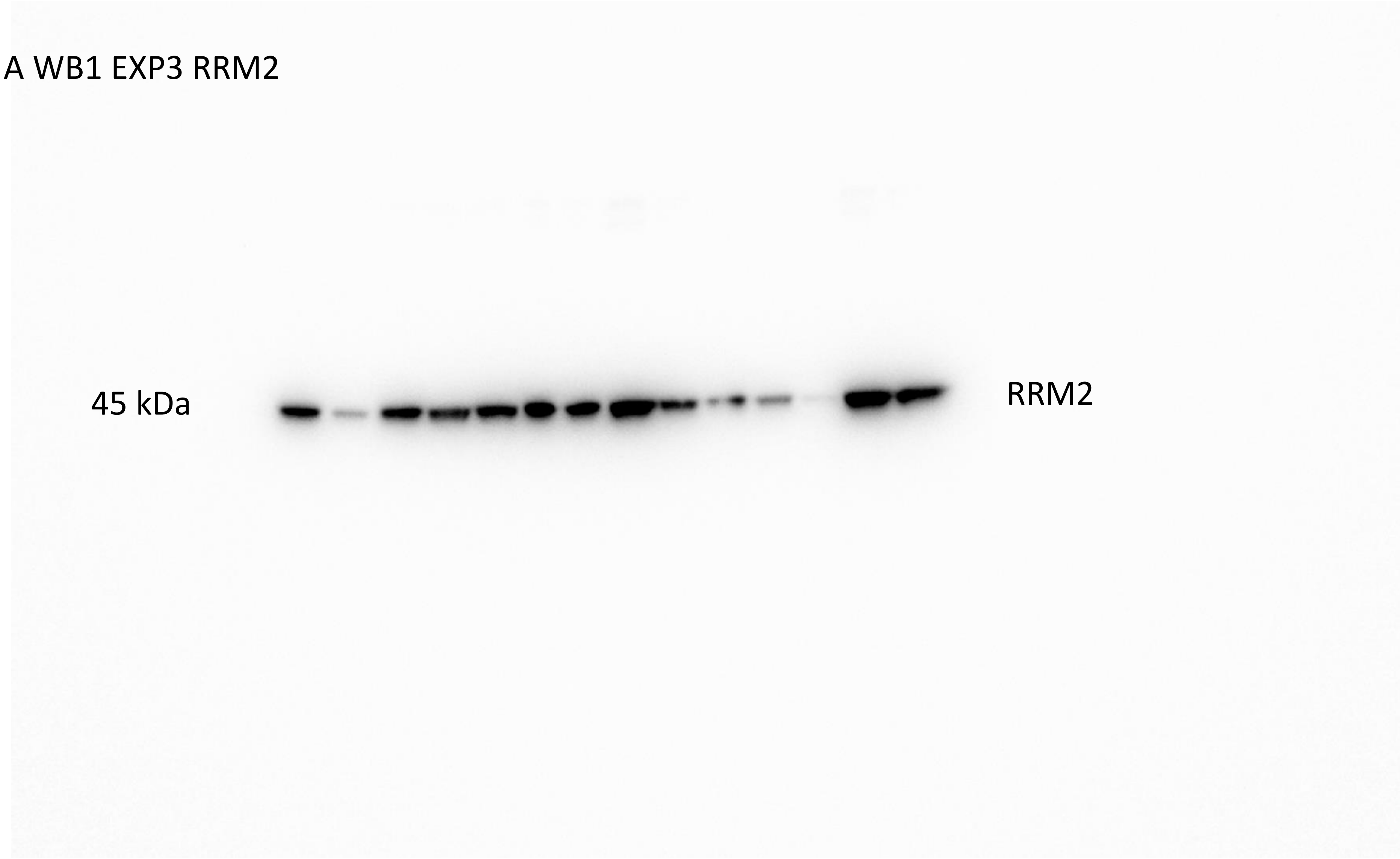

Fig.5 A WB1 EXP3 GAPDH

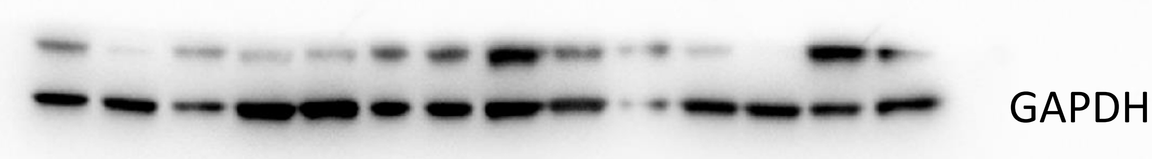

Fig.5 A WB2 RRM2

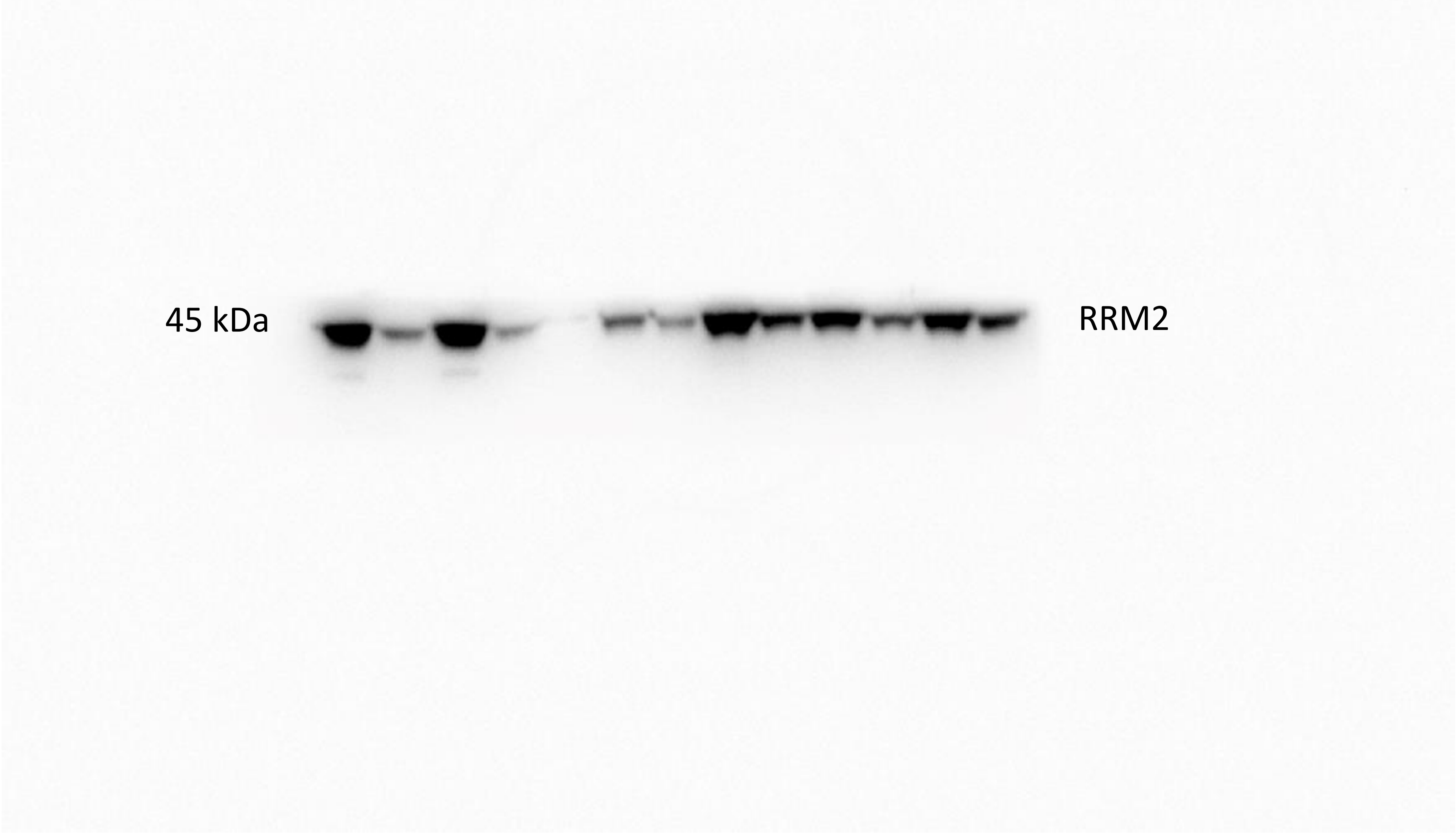

Fig.5 A WB2 GAPDH

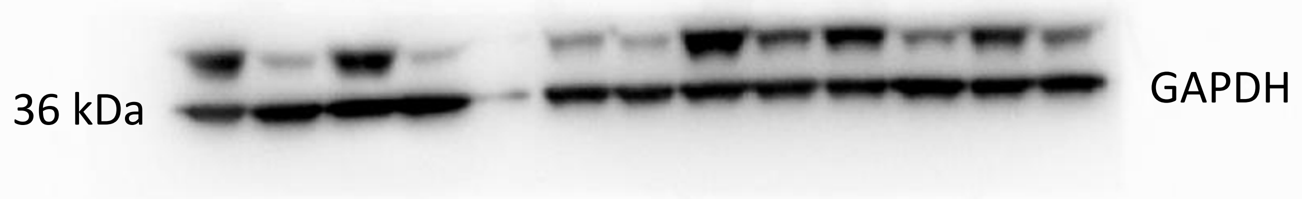

Fig.5 A WB3 RRM2

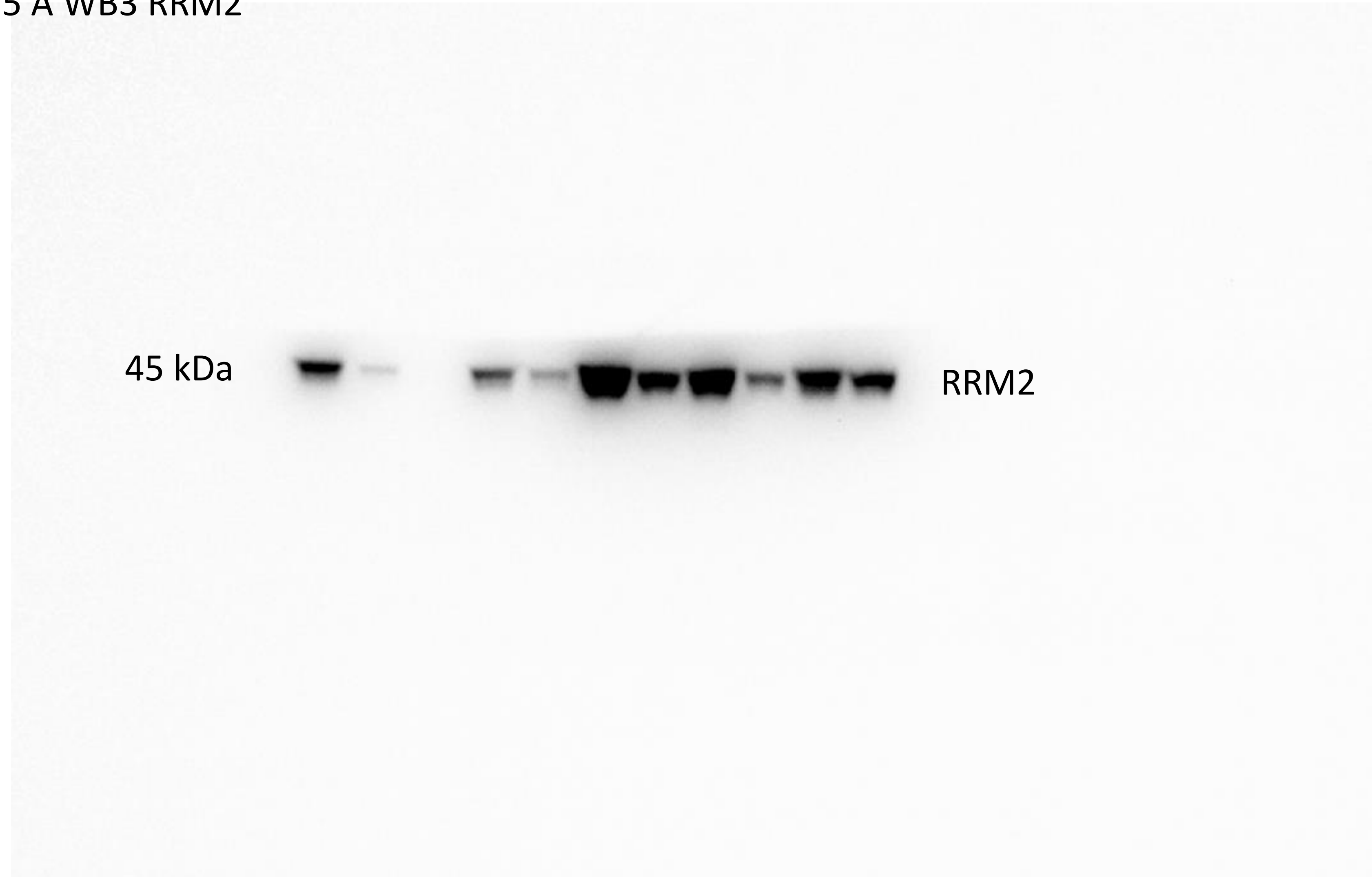

Fig.5 A WB3 GAPDH

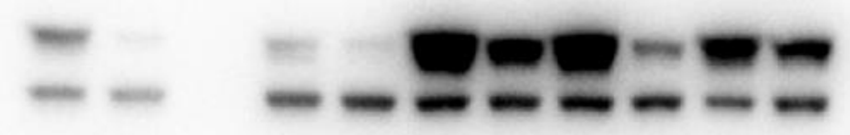

Fig.6 E WB1 RRM2

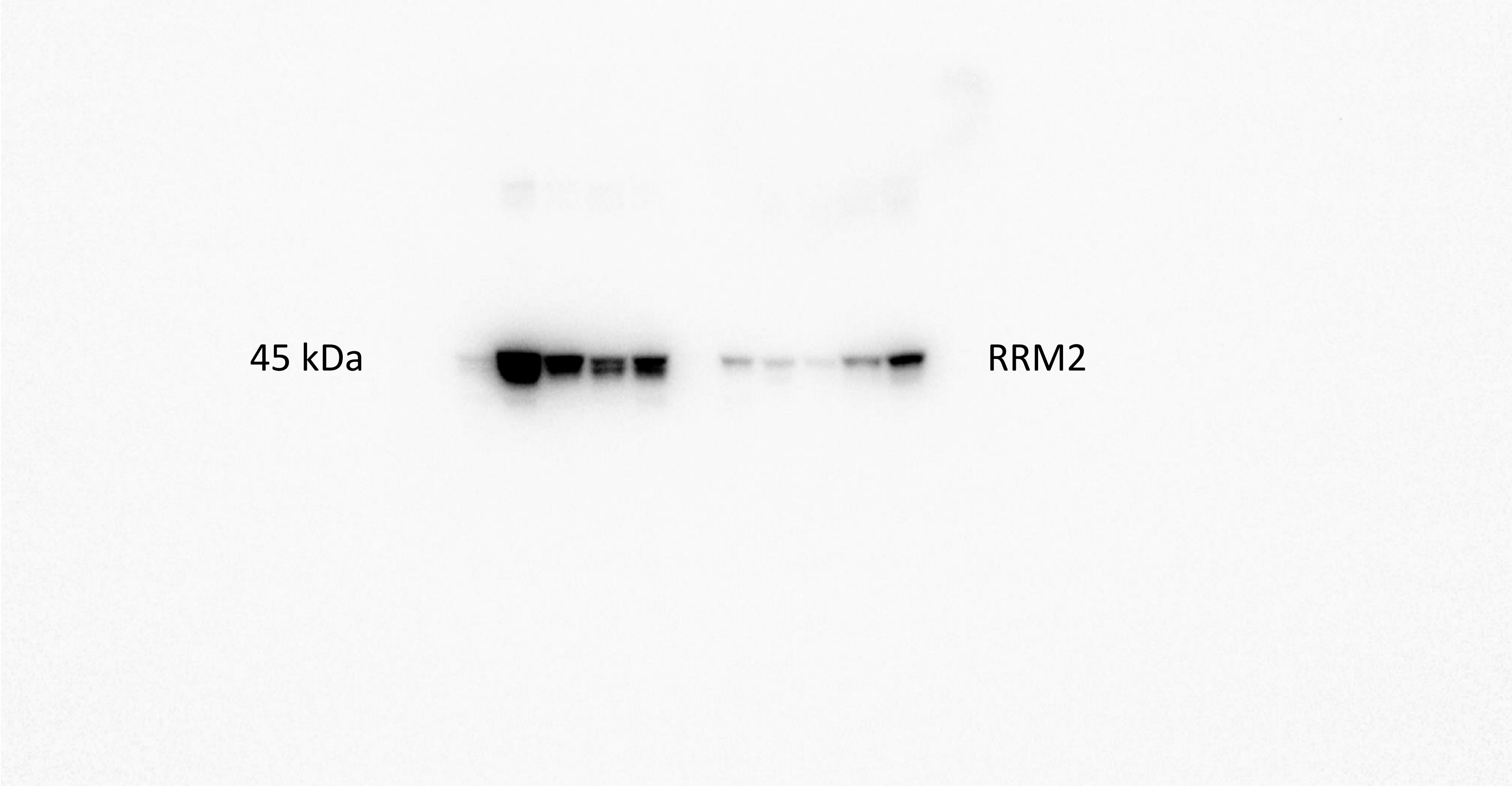

Fig.6 E WB1 GAPDH

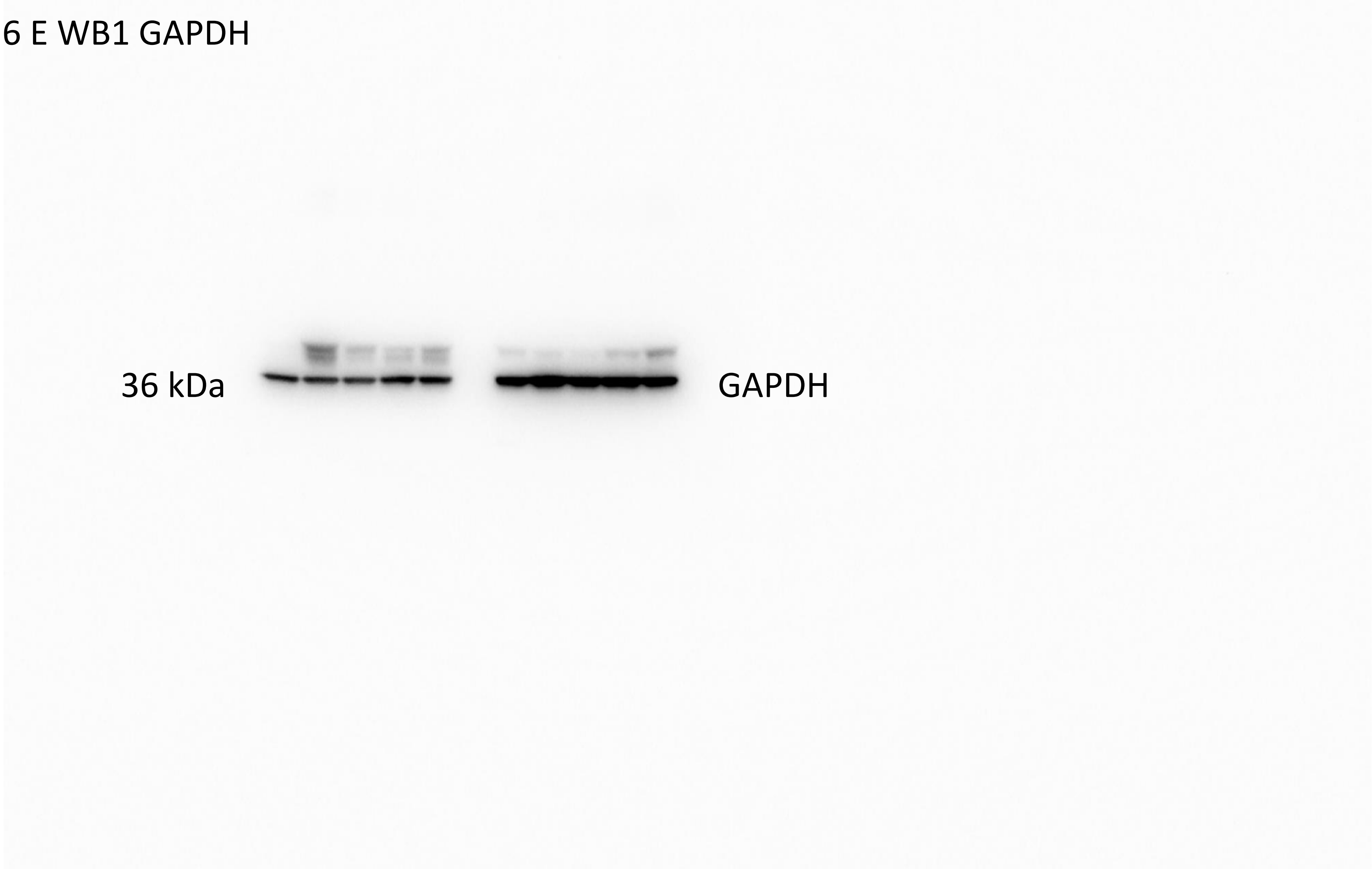

Fig.6 E WB2 RRM2

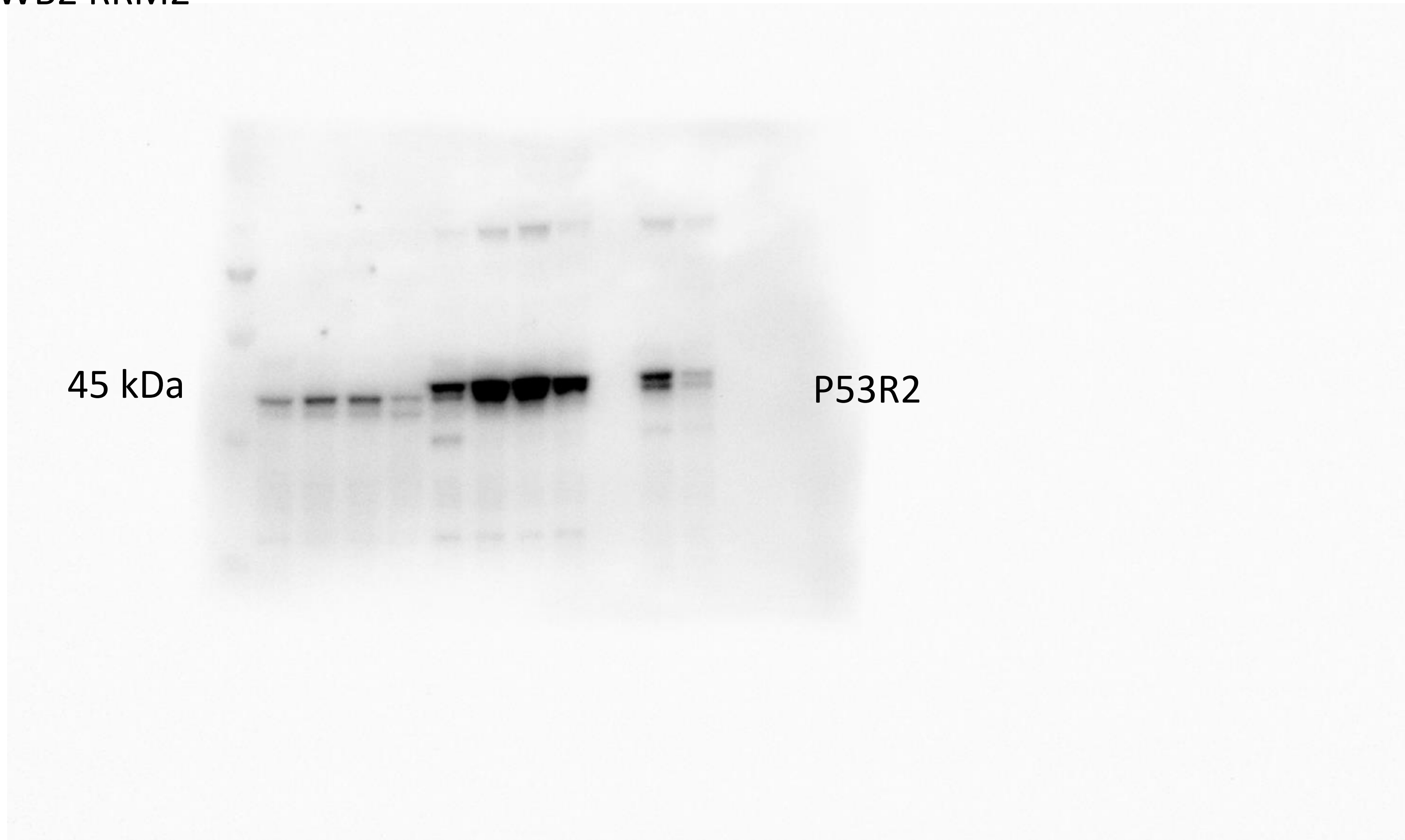

Fig.6 E WB2 GAPDH

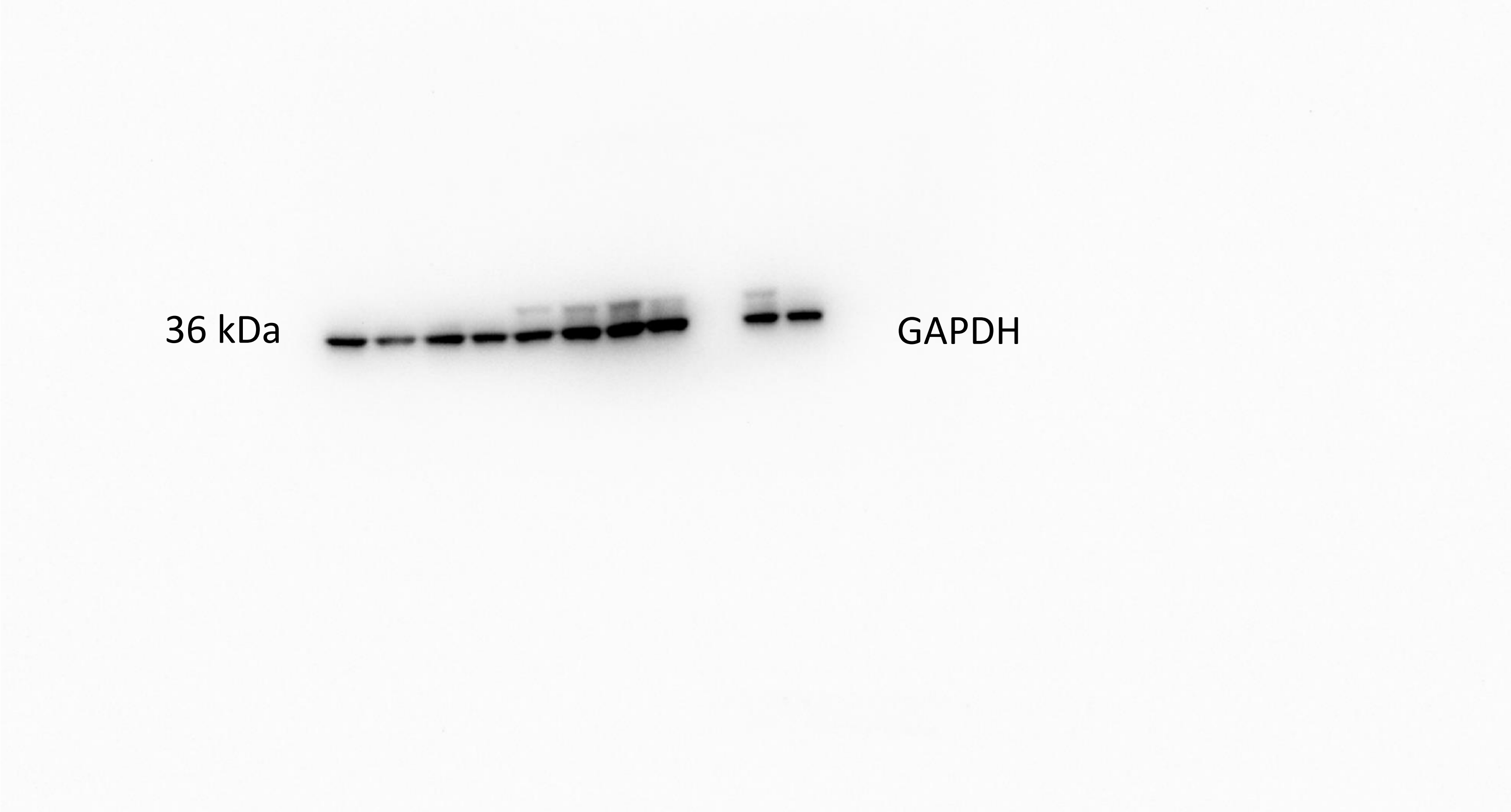

Fig.7 A WB1 p53R2

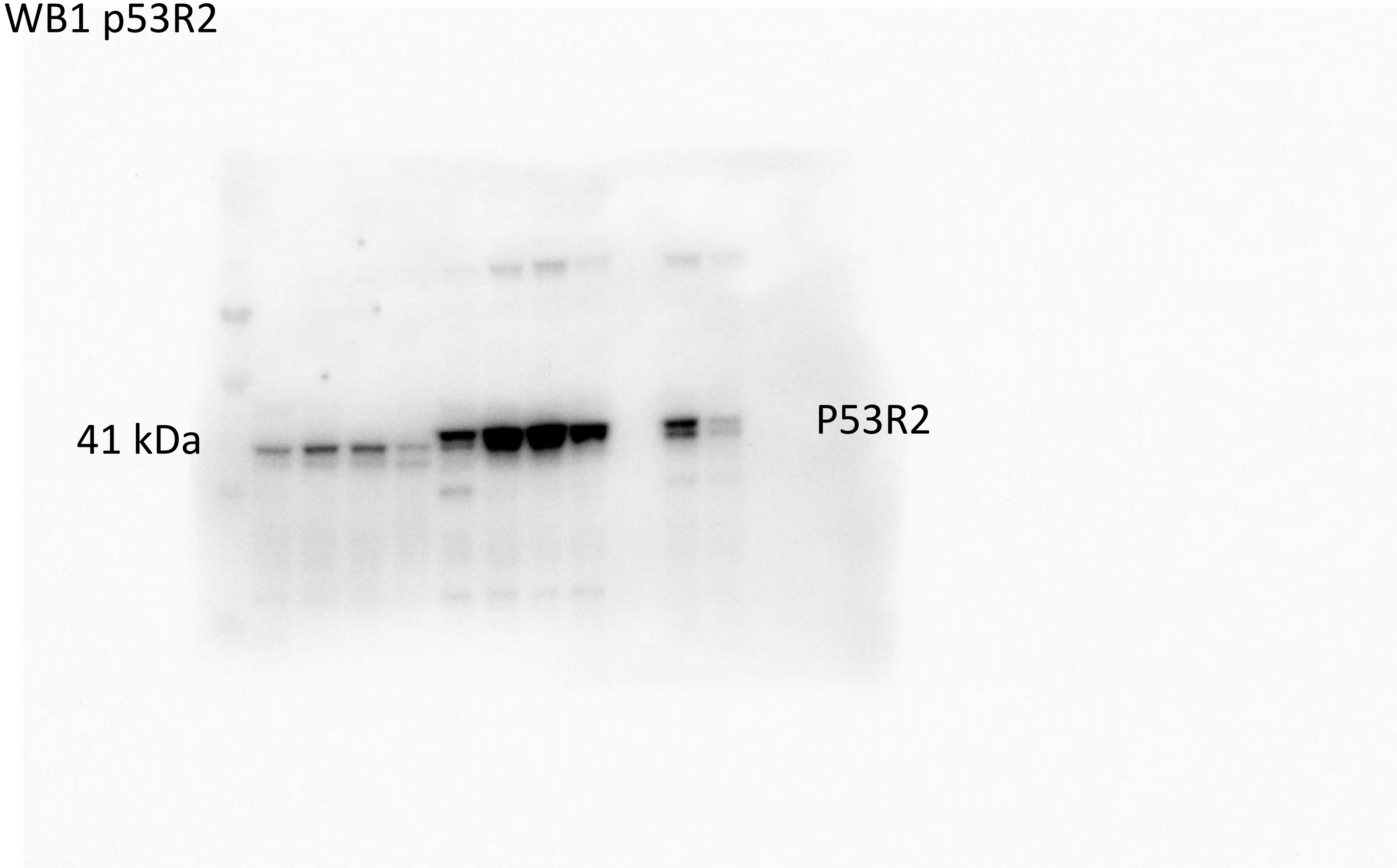

Fig.7 A WB1 GAPDH

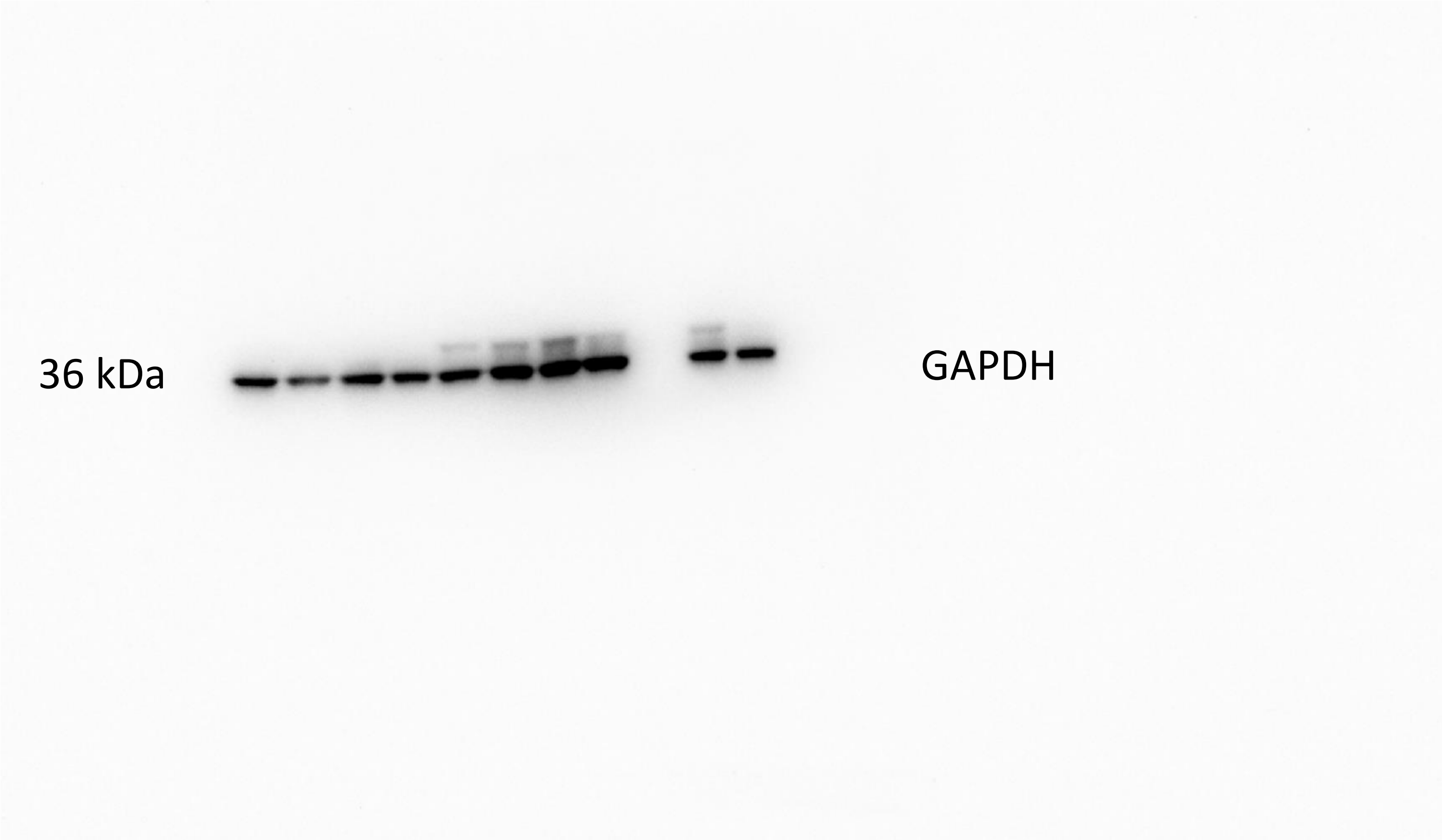

Fig.7 A WB2 p53R2

41 kDa

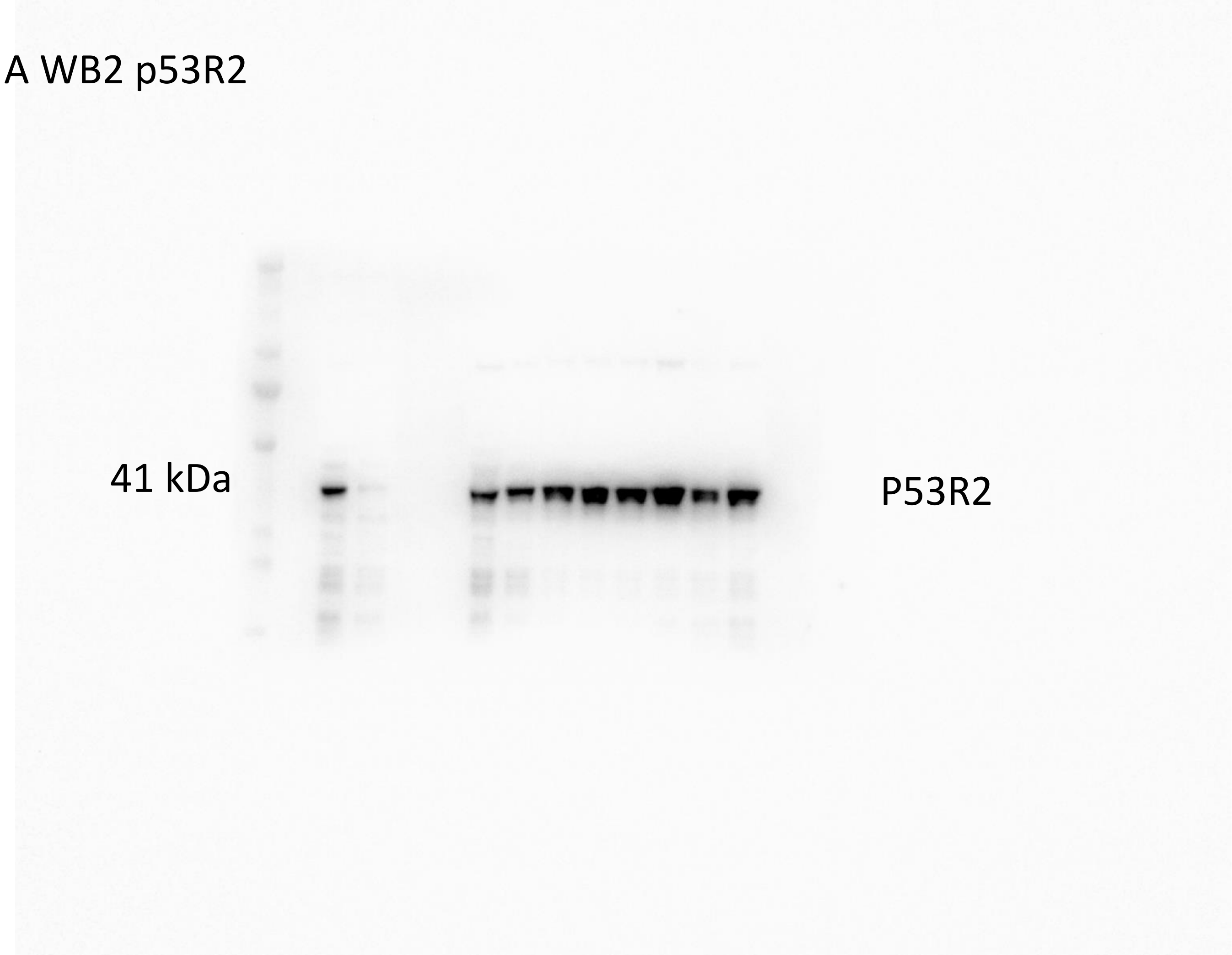

P53R2

Fig.7 A WB2 GAPDH

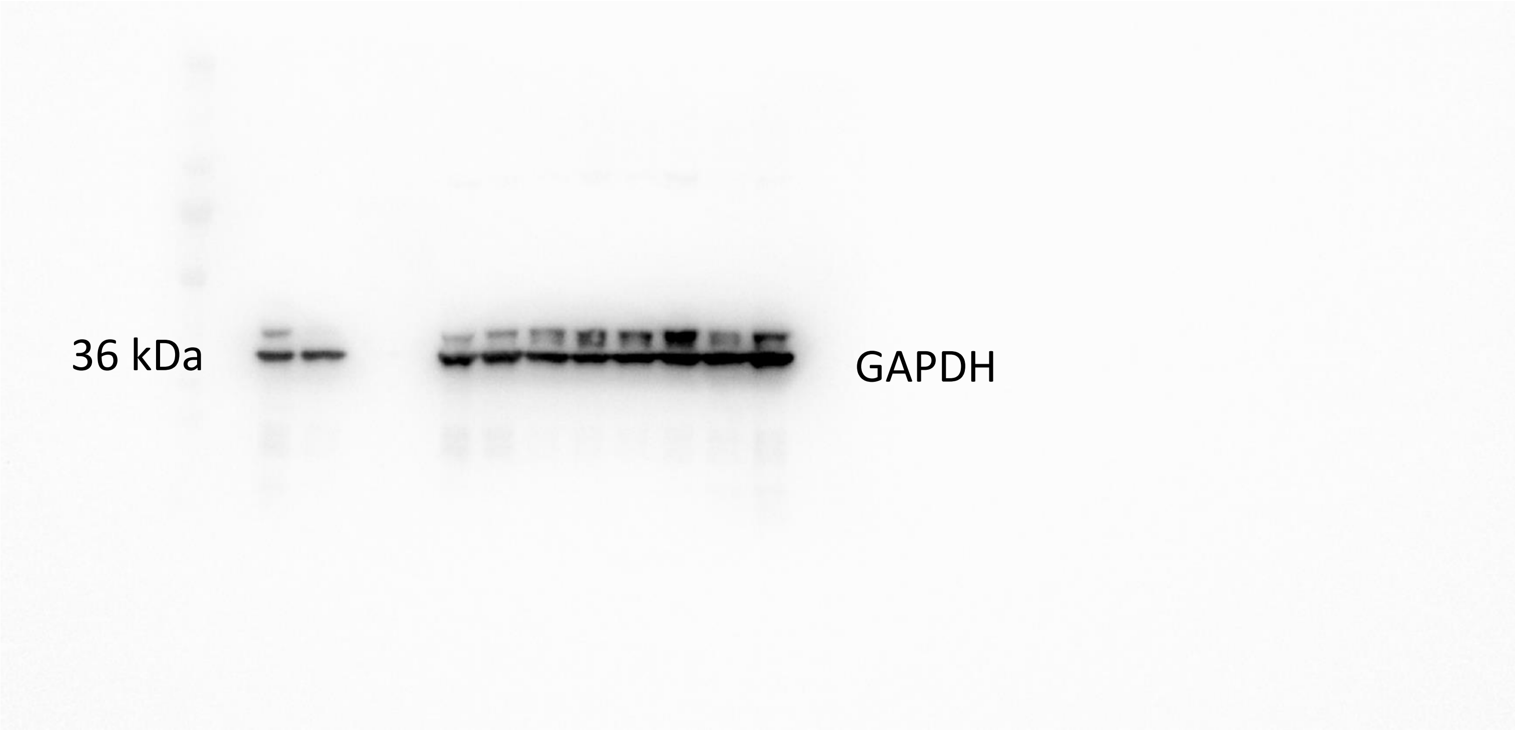

Fig.7 A WB3 p53R2

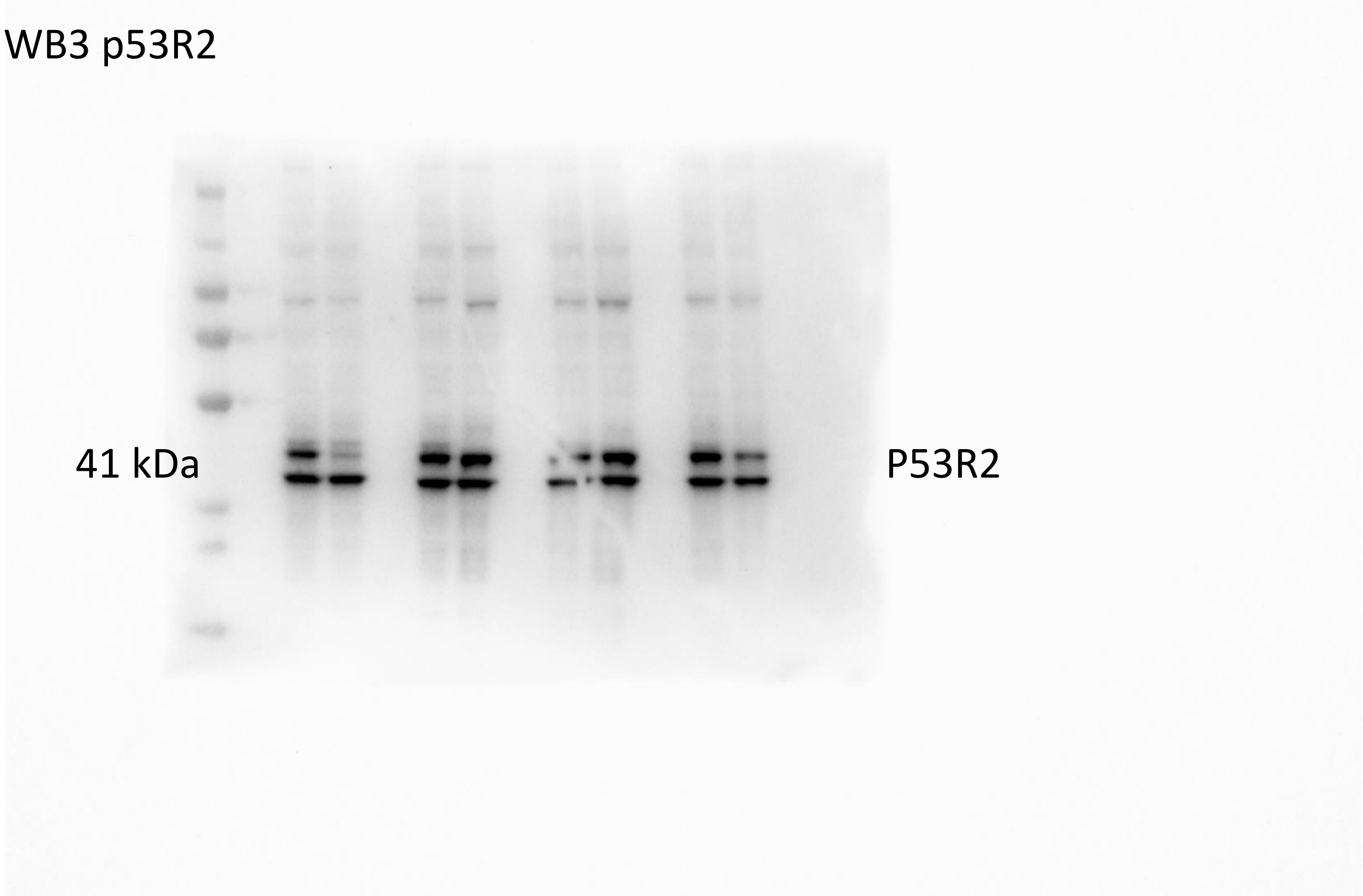

Fig.7 A WB3 GAPDH

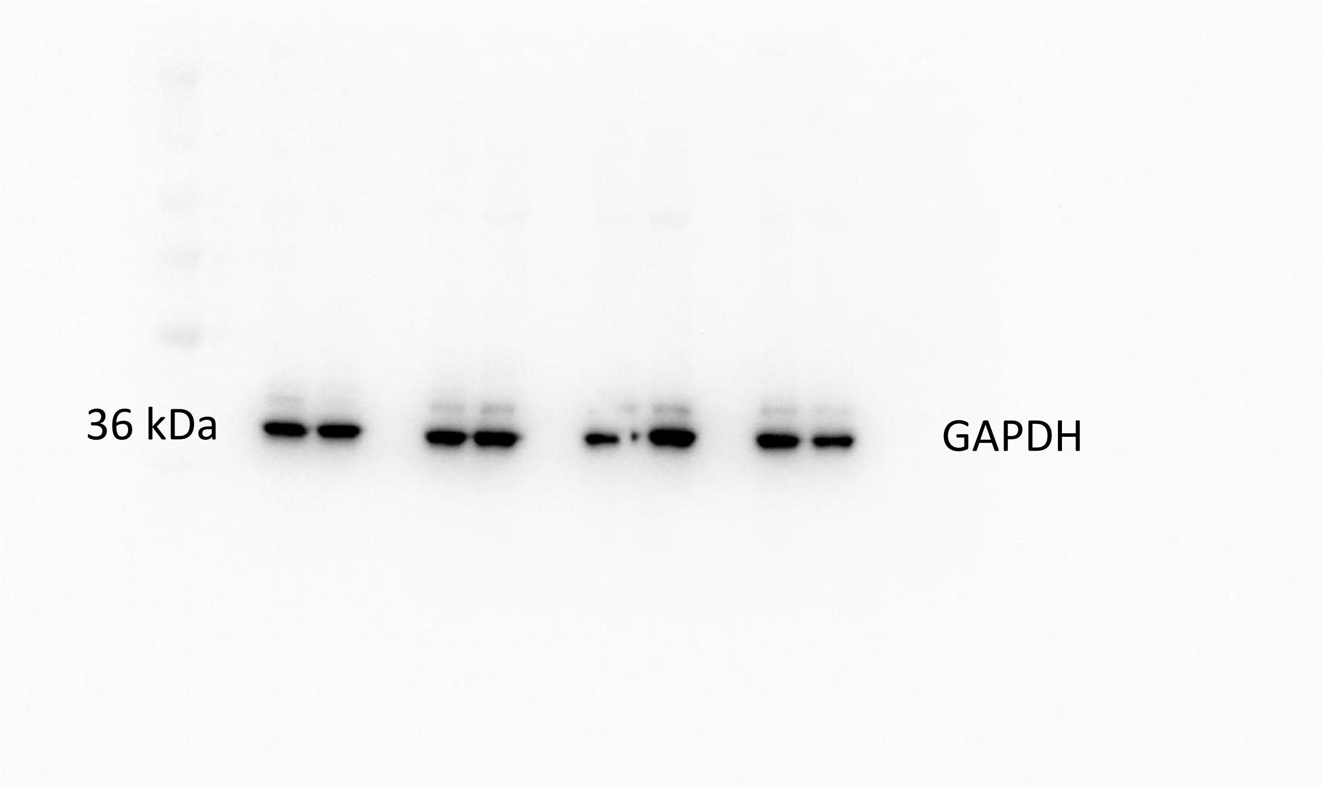

Fig.7 B WB1-3 RRM2

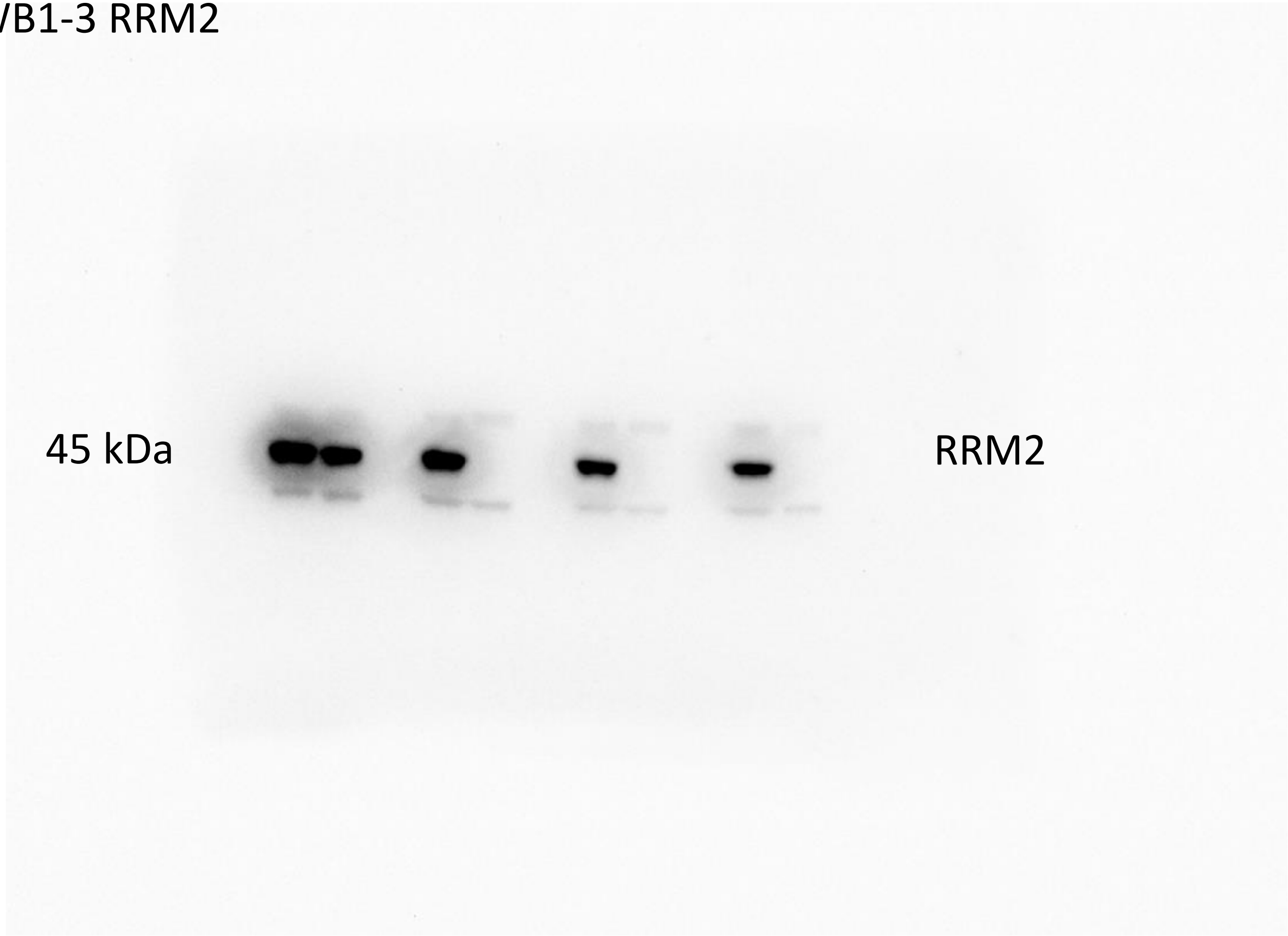

Fig.7 B WB1-3 GAPDH

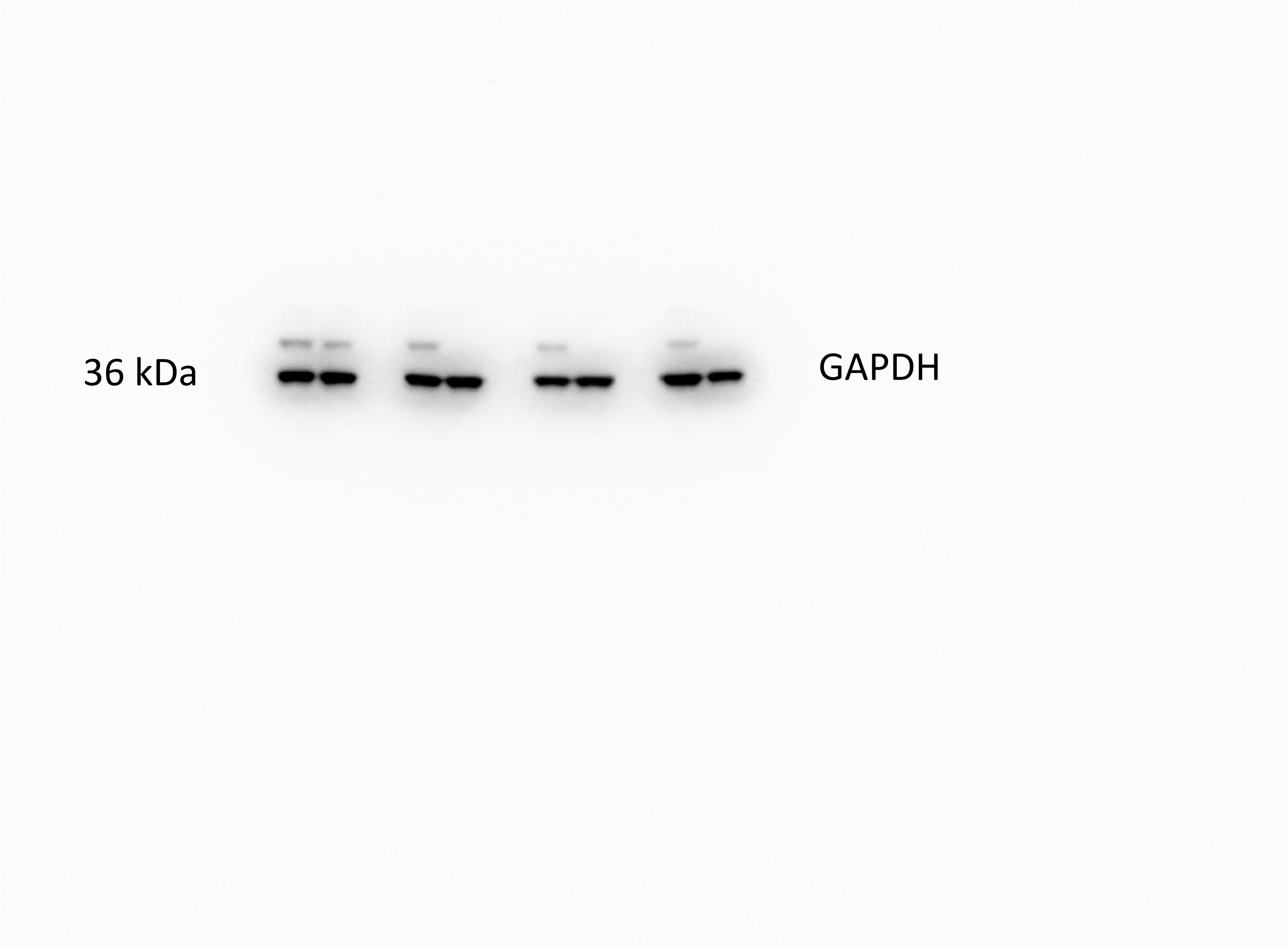

Supplement: Supplementary file 5 — original western blots [file 41420_2026_3105_MOESM5_ESM.pdf]
